# A Comparative Analysis of the Lyve-SET Phylogenomics Pipeline for Genomic Epidemiology of Foodborne Pathogens

**DOI:** 10.3389/fmicb.2017.00375

**Published:** 2017-03-13

**Authors:** Lee S. Katz, Taylor Griswold, Amanda J. Williams-Newkirk, Darlene Wagner, Aaron Petkau, Cameron Sieffert, Gary Van Domselaar, Xiangyu Deng, Heather A. Carleton

**Affiliations:** ^1^Enteric Diseases Laboratory Branch, Centers for Disease Control and PreventionAtlanta, GA, USA; ^2^Center for Food Safety, College of Agricultural and Environmental Sciences, University of GeorgiaGriffin, GA, USA; ^3^Oak Ridge Institute for Science and Education, Oak Ridge Associated UniversitiesOak Ridge, TN, USA; ^4^IHRC, Inc.Atlanta, GA, USA; ^5^National Microbiology Laboratory, Public Health Agency of CanadaWinnipeg, MB, Canada

**Keywords:** SNP pipeline, wgMLST, genomic epidemiology, foodborne, outbreak, bacterial pathogen

## Abstract

Modern epidemiology of foodborne bacterial pathogens in industrialized countries relies increasingly on whole genome sequencing (WGS) techniques. As opposed to profiling techniques such as pulsed-field gel electrophoresis, WGS requires a variety of computational methods. Since 2013, United States agencies responsible for food safety including the CDC, FDA, and USDA, have been performing whole-genome sequencing (WGS) on all *Listeria monocytogenes* found in clinical, food, and environmental samples. Each year, more genomes of other foodborne pathogens such as *Escherichia coli, Campylobacter jejuni*, and *Salmonella enterica* are being sequenced. Comparing thousands of genomes across an entire species requires a fast method with coarse resolution; however, capturing the fine details of highly related isolates requires a computationally heavy and sophisticated algorithm. Most *L. monocytogenes* investigations employing WGS depend on being able to identify an outbreak clade whose inter-genomic distances are less than an empirically determined threshold. When the difference between a few single nucleotide polymorphisms (SNPs) can help distinguish between genomes that are likely outbreak-associated and those that are less likely to be associated, we require a fine-resolution method. To achieve this level of resolution, we have developed Lyve-SET, a high-quality SNP pipeline. We evaluated Lyve-SET by retrospectively investigating 12 outbreak data sets along with four other SNP pipelines that have been used in outbreak investigation or similar scenarios. To compare these pipelines, several distance and phylogeny-based comparison methods were applied, which collectively showed that multiple pipelines were able to identify most outbreak clusters and strains. Currently in the US PulseNet system, whole genome multi-locus sequence typing (wgMLST) is the preferred primary method for foodborne WGS cluster detection and outbreak investigation due to its ability to name standardized genomic profiles, its central database, and its ability to be run in a graphical user interface. However, creating a functional wgMLST scheme requires extended up-front development and subject-matter expertise. When a scheme does not exist or when the highest resolution is needed, SNP analysis is used. Using three *Listeria* outbreak data sets, we demonstrated the concordance between Lyve-SET SNP typing and wgMLST.

**Availability**: Lyve-SET can be found at https://github.com/lskatz/Lyve-SET.

## Introduction

Modern outbreak investigation is enhanced with molecular subtyping evidence. These lines of evidence have been, but are not limited to: pulsed-field gel electrophoresis (PFGE), multiple-locus variable number tandem repeat analysis (MLVA), and multi-locus sequence typing (MLST; MacCannell, 2013). Each of these methods yields specific targets to measure genetic relatedness among pathogens isolated from human cases, animals, foods, or the environment, resulting in evidence for or against their inclusion in a cluster, which in turn aids in epidemiological investigations. In the age of whole genome sequencing (WGS), outbreak investigation is being increasingly supported by phylogenomic methods that are more robust and discriminatory than any aforementioned subtyping method (Jackson et al., 2016). Whether infectious disease outbreaks are caused by single pathogenic clones or by multiple clones, a basic assumption can be made that the epidemiological association between cases can be inferred from the phylogenetic relationships between the case-defining microorganisms. In an outbreak scenario as phylogenetic relatedness increases, the likelihood of epidemiological concordance increases. In other words, phylogeny approximates epidemiology.

There are two dominant methods to create phylogenies for WGS-enhanced outbreak investigations: whole-genome multi-locus sequence typing (wgMLST) and single nucleotide polymorphisms (SNPs). In the wgMLST method for a single genome, as in conventional MLST (Maiden et al., 1998), loci are compared against a database of known alleles and either labeled with a known allele identifier or given a new allele identifier. In MLST and wgMLST, alleles are either the same or different, meaning that any single nucleotide substitution, insertion, or deletion equates to an allele change. With wgMLST, thousands of loci are compared and their distances are used to generate a phylogeny usually with either the unweighted-pair-group-method-with-arithmetic-mean (UPGMA) or neighbor-joining (NJ) algorithm. One implementation of wgMLST is through the BioNumerics software (Applied Maths, Sint-Martens-Latem, Belgium).

In the SNP-based method, single nucleotide changes are used to infer phylogenetic relatedness. This method is implemented in many software packages. Snp-Pipeline has been used for regulatory evidence of *Salmonella enterica* at the Center for Food Safety and Applied Nutrition (CFSAN; Pettengill et al., 2014; Davis et al., 2015). RealPhy has been used to characterize *Clostridium botulinum* outbreaks (Bertels et al., 2014; Shirey et al., 2016). SNVPhyl is used by the National Microbiology Laboratory (NML) of the Public Health Agency of Canada (PHAC) for, among other organisms, *S. enterica* (Bekal et al., 2016). Most SNP-based methods have a common workflow: (1) mapping raw reads onto a reference genome, (2) identifying SNPs, (3) removing lower-quality SNPs, (4) creating a multiple sequence alignment (MSA) from selected SNPs, and (5) inferring a phylogeny from the MSA. When these SNP-based methods remove SNPs with less support, they can be called high-quality SNP-based methods (hqSNP). SNPs with less support can be identified by having few raw reads, by having conflicting allele calls in the raw reads (i.e., low consensus), by occurring in mutation hotspot regions such as phage regions, or for many other reasons. A modification of this typical SNP workflow is implemented in kSNP where nucleotides of odd length *k* (*k*-mers) are extracted from raw reads supplied for the genomes being analyzed (Gardner et al., 2015). Instead of aligning to a reference, the *k*-mers from one genome are compared against the other genome's, where the middle nucleotide can be variable. These variable bases can be extracted into a pseudo-multiple sequence alignment such that a phylogeny can be built. kSNP has been used in describing the population structure of certain foodborne pathogens, e.g., *L. monocytogenes* in cured ham in Italy (Morganti et al., 2015), and in outbreak investigations of other bacterial pathogens, e.g., retrospective analysis of *Legionella pneumophila* (Mercante et al., 2016). These hqSNP pipelines increase the signal-to-noise ratio in favor of a high-quality phylogeny at the risk of removing true but low-quality SNPs.

In 2013, the NML and the Enteric Diseases Laboratory Branch (EDLB) of The Centers for Disease Control and Prevention (CDC) briefly described an initial SNP-based workflow called the SNP Extraction Tool (SET). The initial version of SET was used for the Haiti cholera outbreak of 2010 (Katz et al., 2013). The common code base of SET has since been forked, with the NML branch rebranded as SNVPhyl (Petkau et al., 2016) and the CDC version as Lyve-SET, named after the organisms with which it was first used: *Listeria, Yersinia, Vibrio*, and Enterobacteriaceae. Since 2013, the Centers of Disease Control and Prevention (CDC) has participated in an interagency collaboration to routinely sequence and analyze all clinical and food-related *Listeria monocytogenes* isolates in the US with the eventual goal to replace PFGE (Carleton and Gerner-Smidt, 2016). As WGS data of these isolates are being continuously generated, a phylogenetic framework needs to be constructed and constantly updated to support epidemiological surveillance and outbreak investigation of *L. monocytogenes*. Therefore, upon the onset of the interagency collaboration, we revised and formalized Lyve-SET into a packaged pipeline that suits the needs of bacterial foodborne outbreak investigations. Lyve-SET was refined in the context of *L. monocytogenes* outbreak investigations and continues to be a strong reference tool for *L. monocytogenes* and many other foodborne pathogens such as *S. enterica, Escherichia coli, Yersinia enterocolitica, Cronobacter*, and *Vibrio cholerae*.

Historically, it has been difficult to evaluate and compare SNP pipelines, and an even bigger challenge to compare them to workflows based on other algorithms (e.g., wgMLST). Each of the aforementioned pipelines produces output that can be used for interpreting the relationship between genomes in various forms such as distance matrices, MSAs, and dendrograms; however, they have different underlying algorithms and output formats. For example, each SNP pipeline uses a different read mapper and SNP caller and might produce a different format to describe their SNP calls. In comparing wgMLST and SNP workflows which are wholly different algorithms, one SNP might be located in an intergenic region, yielding zero allelic differences by wgMLST; on the other hand many SNPs might be located on a single gene, yielding the collapse of multiple SNPs into a single allelic difference.

A reasonable approach to pipeline comparison, therefore, might be at the phylogenetic level. A classic comparison method is the Robinson-Foulds metric, sometimes called the symmetric difference metric, where the number of internal branches that exist in one tree but not the other are counted (Robinson and Foulds, 1981). Another metric is Kuhner-Felsenstein, sometimes called “branch score” which is similar to Robinson-Foulds but calculates the Euclidean distance between each branch's length (Kuhner and Felsenstein, 1994). Both Robinson-Foulds and Kuhner-Felsenstein metrics are implemented in the Phylip package in the program treedist (Felsenstein, 1989) and in some programming language libraries such as Bio::Phylo (Vos et al., 2011). Both of these classical metrics rely on unrooted trees, and small differences between two trees can artificially magnify the distance between two trees. A more robust tree metric—the Kendall-Colijn—accounts for both tree topology and branch length (Kendall and Colijn, 2015). The Kendall-Colijn metric compares two rooted trees using Euclidean distances from tip to root with a coefficient λ to give more weight to either topology (λ = 0) or branch length (λ = 1). One more reasonable approach to pipeline comparison is assessing the distance matrices between two workflows. The Mantel test uses a generalized regression approach to identify correlations between two distance matrices (Smouse et al., 1986). Therefore, if the genome distances from one workflow vs. another workflow are consistently higher but correlate well, the Mantel test will yield a high correlation coefficient.

In this article, we describe the Lyve-SET workflow, demonstrate how it can aid in bacterial foodborne outbreak investigations, and propose methods of comparison with other phylogenetic workflows.

## Materials and methods

### Implementation

Lyve-SET is a high quality SNP (hqSNP) pipeline, designed to remove lower-quality SNPs from its analysis and increase phylogenetic signal. Lyve-SET has its origins in the original SET algorithm described in Katz et al. (2013). Major changes in Lyve-SET compared to SET include integrated read cleaning and phage masking, the use of VarScan instead of FreeBayes for SNP calling, improved production of intermediate files in standard formats, and the use of RAxML v8 to infer trees instead of PhyML (Guindon et al., 2010; Garrison and Marth, 2012; Koboldt et al., 2012; Stamatakis, 2014). The source code is available at https://github.com/lskatz/Lyve-SET (v1.1.4f, doi: 10.5281/zenodo.163647).

**Figure 1:**
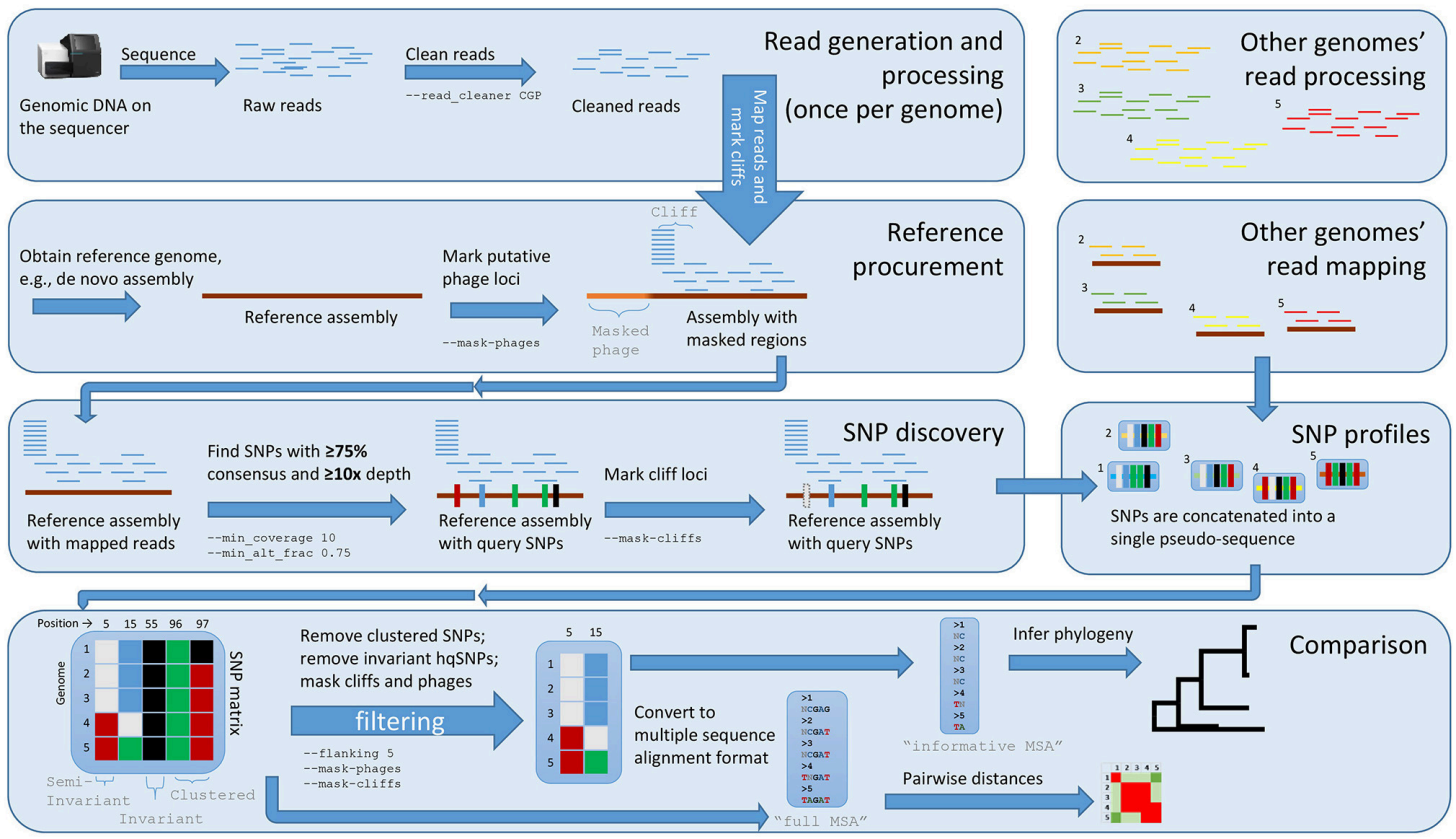
**The Lyve-SET workflow**. Starting from the top left, reads are generated from a single query genome and then compared against a reference genome. Starting from the top right, other genomes are being generated and compared against the reference genome simultaneously. The order is (1) sequence query genome; (2) obtain a reference genome; (3) discover SNPs in a comparison against the reference genome; (4) combine SNP profiles into (5) a SNP matrix. In the bottom portion, the SNP matrix is interrogated for low-quality sites including those that are invariant or semi-invariant (those with masked or reference alleles). The matrix is also interrogated for clustered SNPs, i.e those that appear too close to each other. After the SNP matrix is queried and filtered, Lyve-SET obtains high-quality SNPs which are then used for creating a phylogeny. The larger, unfiltered multiple sequence alignment is used to calculate pairwise distances which can be used in a comparison, e.g., a heat map.

With the default workflow, there is a well-defined audit trail such that it is clear how Lyve-SET was initialized (Table 1) and from where each analysis was derived (i.e., intermediate files are saved). Lyve-SET requires as input a set of raw reads and a phylogenetically related reference genome assembly. Lyve-SET has only been tested with Illumina reads and default settings are optimized for Illumina data, but it can accept FASTQ files from any platform. These steps are depicted in Figure 1.

**Table 1:** **Features of Lyve-SET**.

	**Description**	**Lyve-SET**	**kSNP**	**RealPhy**	**SNP-Pipeline**	**SNVPhyl**
Repeat detection	Detection of repeat elements that could confound SNP results	0^a^	0	0	0	1^a^
Auto-choose reference or reference-free	Independence of a reference genome or a user-defined reference genome to find SNPs	0	1	1	0	0
Removal of distant genomes	Removal of genomes from analysis when they are greater than a certain threshold of SNPs	0	0	0	1	0
Phage detection	Detection and masking of phages	1	0	0	0	0
Cliff detection	Detection and masking of cliffs	1	0	0	0	0
SNP cluster detection	Detection and masking of clustered SNPs	1	0^b^	0	1	1
Read cleaning	Cleaning and trimming of raw reads	1	0	0	0	0
BAM file for each individual genome	Standardized BAM files that describe the locations of mapped reads	1	0	1	1	1
VCF file for each individual genome	Standardized VCF files that describe the locations of SNPs and evidence supporting them	1	1	1	1	1
Pooled VCF file	Standardized VCF file that describes the locations of all SNPs for all genomes in a single file. This file is created with the bcftools merge command	1	0	0	1	0
Fasta alignment of all sites	Standardized fasta file of all sites across the reference genome, whether they are invariant or SNP sites	1	0	1	0	1
Fasta alignment of SNPs	Standardized fasta file of SNP sites	1	1	1	1	1
Standardized tree file	File representing the phylogeny in a standardized format, e.g., Newick	1	1	1	0	1
Settings for different species	Does the pipeline have customizable settings for different species? Lyve-SET has customized settings using the– –presets flag (Table 2)	1	0	0	0	0
Audit trail: repeatability	Displays the path to the SNP pipeline installation and the exact command to repeat the analysis. Lyve-SET provides the command and all explicit and implicit options	1	0	0	1	1
Automated quality control	Reviews the analysis results and describes low-quality results. This quality control can be a review of the length of the multiple sequence alignment, the number of positions masked in each genome, or simply reviewing something minor like the insert length of each genome. Lyve-SET encompasses this quality control step in set_diagnose.pl	1	0	0	1	1

a *Although Lyve-SET does not have repeat detection, it does not allow the short-read mapper to place reads where they map equally well in two locations, i.e., repeat regions. SNVPhyl can perform the same function but also straightforwardly identifies repeat regions in the reference genome*.

b *Although kSNP does not have SNP cluster detection directly, its fundamental algorithm prohibits any SNP from occurring within k-1 bp from each other, where k is the length of the kmer. For example on a kmer value of 5, two SNPs must occur at least 4 bp from each other*.

*Features of Lyve-SET are shown with a comparison of the other SNP pipelines compared in this study. “1” indicates the feature is present; “0” indicates that the feature is absent. A comparison of software-level features, e.g., command-line vs. web interface, has already been performed in Petkau et al. (2016)*.

Although optional, the first recommended step when running Lyve-SET is pre-processing raw sequencing reads. When using the – –read_cleaner option, reads are cleaned with CG-Pipeline (Kislyuk et al., 2010). The default Lyve-SET options for the CG-Pipeline read cleaner are – –min_quality 15 – –min_avg_quality 20 – –bases_to_trim 100 which signifies that each read will be trimmed from the 5′ and 3′ ends up to 100 bp, until a nucleotide has at least a Phred quality of 15. Then, any read with less than an average quality of 20 will be removed. Accordingly, lower-quality reads are removed, trimmed, and/or corrected. Next, phage genes are discovered in the reference genome using BLASTx against the PHAST database with a custom script set_findPhages.pl (Camacho et al., 2009; Zhou et al., 2011). A single transduction within an outbreak can introduce changes in thousands of sites when in reality, it is only a single evolutionary change. For example, this event has been observed in *L. monocytogenes* recovered from Italian cheese products in 2012 (Bergholz et al., 2015). Therefore, phage genes on the reference genome are masked in an optimal Lyve-SET analysis. The masked regions are recorded in a BED-formatted file, and a user can also manually edit this file to exclude any other troublesome regions.

The second step is mapping reads of each genome against the reference assembly by SMALT using the launch_smalt.pl script (Ponstingl and Ning, 2010). To achieve high-quality mapping, each read's match to the reference must be 95% identity or above. The expectation in a single-source outbreak is that there will be very few hqSNPs in a dataset; therefore, this identity threshold should help maintain a high accuracy of read-mappings while removing unrelated and error-prone reads. Additionally, the match must be unambiguous within the reference genome—i.e., it cannot match elsewhere equally well—so that repeat regions are masked. One more filter can be optionally applied to avoid calling SNPs in “cliffs.” A cliff is when the read coverage rises or falls dramatically, possibly due to repeat regions, sequencing anomalies, or other factors. To detect these cliffs, a stand-alone script set_findCliffs.pl was developed. This script creates a linear trend line in window sizes of 10 base pairs (bp). If the slope of coverage is >3 reads per bp or <−3 reads per bp, then the region is masked, and SNPs will not be called at the particular locus in the particular genome.

The third step is SNP-calling from the read alignments of each genome. Lyve-SET employs the mpileup2cns method of VarScan v2.3.7 to find and detect SNPs using the launch_varscan.pl script (Koboldt et al., 2012). In this way, VarScan identifies the nucleotide of the query genome at each position of the reference assembly. Any site that has <75% consensus, fewer than 10 reads, or does not have at least two forward and two reverse reads is masked. In the resulting MSA, these masked sites are identified as “N.” Foodborne bacterial pathogens are haploid and so any SNP should be supported by much more than a 50% consensus. Enforcing at least 10 reads at a site helps ensure that a variant is not a random error on a single or few raw reads. Together, these three thresholds increase support at the SNP-calling stage. However, these values are user-customizable and not hard-coded. In our own investigations, we have modified the settings for many species including *S. enterica, E. coli*, and *L. monocytogenes* (Table 2). These presets were empirically determined with ongoing outbreaks. Over time, they helped the Lyve-SET results agree with known epidemiology, and so we recorded them. These settings are documented in a configuration file and can be invoked with the Lyve-SET – –presets option.

**Table 2:** **Presets for Lyve-SET**.

**Name**	**Settings**
lambda	min_coverage = 4 min_alt_frac = 0.75 mask-phages = 0
vibrio_cholerae	min_coverage = 10 min_alt_frac = 0.75
listeria_monocytogenes	min_coverage = 10 min_alt_frac = 0.75
salmonella_enterica	min_coverage = 20 min_alt_frac = 0.95 allowedFlanking = 5 mask-phages = 1
escherichia_coli	min_coverage = 20 min_alt_frac = 0.95 allowedFlanking = 5 mask-phages = 1
clostridium_botulinum	min_coverage = 10 min_alt_frac = 0.75 allowedFlanking = 5 mask-phages = 1 mask-cliffs = 1

*Some settings that have been empirically determined are in a configuration file in the Lyve-SET package. These settings can be revised by individual users in the file presets.conf. For many species such as Campylobacter jejuni, we have not yet determined the most optimal preset options. However in the future these settings could be added upon or revised following any observations we may make in the due course of outbreak investigations. In each Lyve-SET run, these settings and their values are displayed in the log file, whether or not they were explicitly defined and whether or not the preset configurations were explicitly called*.

The fourth step in Lyve-SET is the creation of a SNP matrix with the mergeVcf.sh and set_processPooledVcf.pl scripts. Files are merged with the command bcftools merge which creates a pooled VCF file. Next, the pooled VCF file is queried with bcftools query to create a tab-delimited matrix consisting entirely of SNPs. If requested, the matrix is also filtered to remove sites with ambiguous nucleotides, invariant sites, and/or clustered SNPs. The resulting matrix or filtered matrix contains only SNPs that pass all filters and therefore contains only hqSNPs. The user may also request annotations for all SNPs if the reference genome is in the GenBank format using SnpEff via the launch_snpEff.pl script (Cingolani et al., 2012).

Lyve-SET's fifth step is to convert the SNP matrix to a FASTA-formatted MSA. Using the script pairwiseDistances.pl on the MSA, Lyve-SET measures pairwise distances which are helpful in approximating relatedness between taxa. Finally, a phylogeny is inferred using RAxML v8 with the FASTA file containing only hqSNPs, which applies a model for ascertainment bias (Stamatakis, 2014).

All Lyve-SET output files and most intermediate files conform to standardized file formats. Therefore, all results can be viewed in other software if necessary.

### Outbreak clusters

Twelve outbreak clusters of four major foodborne pathogens were queried from the PulseNet database (Table 3; Swaminathan et al., 2006). PulseNet is a national laboratory network that tracks the subtypes of bacteria causing foodborne illness cases to detect outbreaks. The inclusion or exclusion of isolates for each outbreak was determined using evidence gathered during outbreak investigations including WGS, molecular subtyping, demographic, and exposure data. Isolates from each outbreak were identified; however, some outbreak isolates were excluded when differing from the main outbreak clade by 200 or more hqSNPs. To place these outbreak genomes in a global context, we queried the NCBI *k*-mer trees from April 2016 (Accessions: PDG000000001.428, PDG000000002.629, PDG000000003.184, PDG000000004.427; Data Sheet 1). Each NCBI *k*-mer tree is generated by the NCBI Pathogen Detection Pipeline and is a dendrogram of all publically available genomes (https://www.ncbi.nlm.nih.gov/pathogens). Briefly, NCBI creates high-quality genome assemblies. The MinHash algorithm is applied to each genome, and a Jaccard distance is calculated between each pair of genomes. Then, NCBI creates a tree based on the Jaccard distances. Closely related genomes are refined into subclades using SNPs found among these related assemblies, and a subtree is created with FastME (Lefort et al., 2015). After locating the outbreak clade, we advanced one to three ancestral nodes to acquire a population of potentially related descendent genomes (Data Sheet 2). True positive (TP) isolates are those identified to be associated with the outbreak by PulseNet; true negative (TN) isolates are not associated with the outbreak. For each bioinformatics pipeline to calculate sensitivity (Sn) and specificity (Sp), we also needed to find misidentified genomes, namely the false positives (FP), and false negatives (FN). Sn is calculated as TP/(TP+FN); Sp is calculated as TN/(TN+FP).

**Table 3:** **List of outbreaks**.

**Outbreak code**	**Species**	**In outbreak^a^**	**References**
1308MDGX6-1	*L. monocytogenes*	39, 7, 0	Chen et al., submitted
1408MLGX6-3WGS	*L. monocytogenes*	19, 64, 1	Jackson et al., 2015; Timme et al., in review
1411MLGX6-1WGS	*L. monocytogenes*	28, 16, 0	CDC, 2015
1504MLEXH-1	*E. coli*	17, 2, 0	Tataryn et al., 2014
1405WAEXK-1	*E. coli*	6, 4, 4	CDC, 2014; Timme et al., in review
1407MNEXD-1	*E. coli*	6, 10, 1	Health MDo, 2014
1203NYJAP-1	*S. enterica*	55, 8, 0	Hoffmann et al., 2016; Timme et al., in review
1409MLJN6-1	*S. enterica*	9, 29, 0	N/A
1410MLJBP-1	*S. enterica*	5, 10, 0	N/A
0810PADBR-1	*C. jejuni*	14, 111, 0	Marler-Clark, 2008; Timme et al., in review
1509VTDBR-1	*C. jejuni*	8, 8, 0	N/A
1602VTDBR-1	*C. jejuni*	6, 10, 0	N/A

a *The number of isolates associated with the outbreak, the number of isolates not associated with the outbreak, and the number of isolates with unknown status. Those with unknown status were not used in calculations for tree sensitivity and specificity*.

*Each outbreak is shown with counts of outbreak-associated and non-outbreak-associated isolates*.

### Pipeline parameters

In the following, “out” is the project output directory. The versions of each of these workflows was the most up to date from all stable versions at the time of this work, and default parameters were used unless otherwise specified. All wrapper scripts used for these SNP pipelines can be found at https://github.com/lskatz/Lyve-SET-paper.

#### Lyve-SET v1.1.4f

The – –presets flag was set according to each taxon (Table 2). In this example, the taxon is listeria_monocytogenes.

launch_set.pl – –numcpus 12 – –read_cleaner CGP – –presets listeria_monocytogenes out

#### KSNP3 v3.0.0

Reference_in.txt contains the reference genome assembly used in the other reference-based methods. Because kSNP is the only SNP pipeline in this study that does not use nucleotide quality scores, the reads were cleaned before running it. CG-Pipeline was used to clean each read set as shown below, where “uncleaned.fastq.gz” is the original interleaved read set, “sampleDir” is the reads directory used by kSNP, and “cleaned.fastq” is the cleaned interleaved reads. The other specified parameters encode that up to 50 bp were trimmed, the other parameters were auto-picked, and broken pairs were not retained.

run_assembly_trimClean.pl -i uncleaned. fastq.gz -o sampleDir/cleaned.fastq – –bases_to_trim 50 – –auto – –nosingletons

kSNP3 -k 31 -annotate reference_in.txt -all_annotations -in in.txt -core -ML -min_frac 0.75 -CPU $NSLOTS -NJ -vcf -outdir out

#### RealPhy v112

REALPHY_v112 out/samples out/out-readLength 250 -ref reference

#### Snp-pipeline v0.5.2

run_snp_pipeline.sh -c out/snppipeline.conf -s $scratch_out/samples -m copy -o out reference.fasta

#### SNVPhyl v1.0

The CLI version of SNVPhyl was run inside of a docker container. Additionally, SNPs were filtered based on density, with the default threshold set to 2 SNPs within a 20 bp window.

snvphyl.py – –deploy-docker – –fastq-dir fastqs/ – –reference-file reference.fasta – –min-coverage 15 – –min-mean-mapping 30 – –alternative-allele-ratio 0.75 – –run-name name – –filter-density-window 20 – –filter-density-threshold 2 – –repeat-minimum-length 150 – –repeat-minimum-pid 90 – –output-dir out

#### BioNumerics v7.5

wgMLST analysis was performed using tools in the graphical user interface of BioNumerics 7.5 (Applied Maths, Sint-Martens-Latem, Belgium). Briefly, alleles were identified by both an assembly-free k-mer based approach using raw reads and assembly-based BLAST approach based on SPAdes v3.5.0 assembled genomes using the wgMLST *L. monocytogenes* database built in BioNumerics 7.5 (Bankevich et al., 2012). This database contains 4804 loci representing 1748 loci from the Institute Pasteur core scheme (Moura et al., 2016) and 3056 loci representing the pan-genome of *L. monocytogenes* identified from publicly available reference sequences. Once all alleles were assigned to each genome, an unweighted-pair-group-method-with-arithmetic-mean (UPGMA) tree was constructed based on all loci among all the genomes.

### Statistical tests

Three categories of pipeline comparisons were performed: the Sn and Sp of outbreak isolates included in the target outbreak clade, tests of tree topology, and tests of variant positions and distances.

#### Comparing trees

If an isolate fell into the same well-supported clade as outbreak isolates (node confidence value ≥70%), it was counted as a positive. Otherwise, it was counted as a negative. Positives that retrospectively agree with the outbreak investigation were counted as TP; otherwise, FN. The target well-supported clade is defined as a having a confidence value >70% and being as complementary as possible for Sn and Sp for each tree. Sn was calculated as TP/(TP+FN). Sp was calculated as TN/(TN+FP). For the following statistical scripts, Perl v5.16.1 and R v3.3.0 were used.

To compare trees, we implemented the Kendall-Colijn and Robinson-Foulds tests (Robinson and Foulds, 1981; Kendall and Colijn, 2015). The Kendall-Colijn test was implemented in the R package Treescape v1.9.17. The background distribution of trees is a set of 10^5^ random trees using the APE package in R (Paradis et al., 2004). Each random tree was created with the R function rtree, with the taxon names shuffled. The query tree was compared against the background distribution and then against the Lyve-SET tree. A Z-test was performed; a *p* < 0.05 indicates that the query tree is closely related to the Lyve-SET tree. The Robinson-Foulds metric, also known as the symmetric difference, was implemented in the Perl package Bio::Phylo and was compared against 10^5^ random trees generated in BioPerl (Stajich et al., 2002; Vos et al., 2011). The query tree, i.e., an observed tree from wgMLST or from a SNP pipeline, was compared against the random distribution and against the Lyve-SET tree. A Z-test was performed to compare the distances against the random distribution and the distance vs. Lyve-SET. A significant *p*-value (α < 0.05) indicates that the query tree is more closely related to the Lyve-SET tree topology than would be expected by chance. Given that low-confidence nodes would not be considered during an outbreak investigation, we removed low-confidence nodes (bootstrap support <70%), potentially creating multifurcating trees, before performing the Kendall-Colijn test. From this transformation, 47 out of 63 trees became multifurcating for this comparison. Only one Lyve-SET tree, the three wgMLST trees, and 12 RealPhy trees remained binary. The Robinson-Foulds test does not tolerate multifurcation; therefore low-confidence nodes were not removed for those tests. Unless otherwise indicated, all trees were midpoint-rooted. All statistical scripts used in this study are available at https://github.com/lskatz/Lyve-SET-paper.

#### Comparing distances and SNP locations

To compare genetic distances, we plotted each pairwise distance between genomes into a scatter plot, with the x-axis representing Lyve-SET SNPs and the y-axis representing the distance calculated from the other pipeline. This produced one scatter plot per outbreak dataset. Additionally, we used linear regression analysis on each dataset to create a trend line with a slope indicative of calculated distance per Lyve-SET SNP and an *R*^2^ value indicative of goodness-of-fit. We combined all datasets into graphs of each of the four species in this study. Because Lyve-SET is mainly used for outbreak datasets, we also produced scatter plots which only included outbreak-associated genomes such that we could limit the influence of non-outbreak-associated isolates. Jackson et al. (2016) reported an empirical 50-hqSNP distance between outbreak isolates. We observed similar maximum thresholds for all 12 outbreaks in this study for each species. Some distances in the outbreak-only scatter plots were outliers with a clear separation between <50 and >100 on the distance axis (Data Sheet 3); therefore in the context of analyses comparing only within outbreak-associated isolates, distances > 100 from non-Lyve-SET pipelines were removed.

We also assessed the correlation between the pairwise distance matrices directly using the Mantel test implemented in the R package Vegan v2.4.0 using the Spearman correlation and 1000 permutations (Mantel, 1967; Oksanen et al., 2007). Each query was compared against Lyve-SET.

To compare SNP positions, the set of SNPs from Lyve-SET was used as reference even though no one pipeline can predict with 100% confidence the correct locations of all SNPs. If a query SNP agreed with the position of the Lyve-SET SNP, it was considered as a TP; if the pipeline excluded a position as a SNP that Lyve-SET excluded, then it was a TN. Sn and Sp were calculated as in the test for Sn/Sp of outbreak isolates.

## Results

### Evaluation of SNP pipelines using outbreak data sets

In general, all SNP pipelines in the comparison ascribe outbreak isolates to the outbreak clade with 100% Sn (Table 4, Data Sheet 4). The one exception is that Snp-Pipeline misclassified a clade of three isolates in the *Salmonella* 1203NYJAP-1 dataset (Table 3). Additionally in most instances, the pipelines have 100% Sp as well. Notably for *C. jejuni*, all pipelines yielded 100% Sn and Sp; for *L. monocytogenes* and *E. coli*, all pipelines but one yielded 100%. The *S. enterica* outbreak data caused some difficulty with less-than-perfect Sn and Sp scores for all six pipelines. For four outbreaks associated with *L. monocytogenes, E. coli*, and *S. enterica*, kSNP yielded <100% Sp meaning that some isolates not associated with the outbreak were found in the outbreak clade. Additionally the trees of each pipeline were compared against Lyve-SET (Data Sheets 1, 2). The Robinson Foulds test reported *p* < 0.05 with the exception of the kSNP trees for outbreaks 1405WAEXK-1 (*E. coli, p* < 0.625) and 1410MLJBP-1 (*S. enterica, p* < 0.674). However, according to the Kendall-Colijn test for topology (when λ = 0), at least one tree per pipeline yields a *p*-value > 0.05.

**Table 4:** **Summary of 12 pipeline comparisons**.

	**Lyve-SET**	**kSNP**	**RealPhy**	**Snp-Pipeline**	**SNVPhyl**	**wgMLST**
Tree sensitivity (Sn)^a^	100.0%	100.0%	100.0%	100.0%	100.0%	100.0%
Tree specificity (Sp)^a^	100.0%	90.2%	100.0%	100.0%	100.0%	100.0%
Average of Sn and Sp	100.0%	95.1%	100.0%	100.0%	100.0%	100.0%
Kendall-Colijn (λ = 0)^b^	–	1.26E-02	7.51E-03	9.28E-03	9.15E-02	1.00E-04
Robinson-Foulds^b^	–	3.16E-69	6.79E-40	5.39E-74	9.61E-49	1.55E-147
Mantel	–	0.60	0.77	0.77	0.79	0.74
SNP ratio^c^^,^^d^	–	0.53, 0.78	0.97, 0.84	1.61, 1.75	0.67, 0.84	0.69, 0.72
Goodness-of-fit (*R*^2^)^d^	–	0.46, 0.42	0.7, 0.75	0.77, 0.3	0.83, 0.68	0.75, 0.72
Genome analyzed^e^	25.9%	0.1%	84.8%	0.3%	82.1%	88.2%

a Average percentage from 11 outbreaks. The S. enterica outbreak 1203NYJAP-1 was removed as an outlier because all pipelines except wgMLST produced errors with grouping outbreak vs. non-outbreak isolates. Therefore this dataset was removed from the Sn and Sp calculations as an outlier.

b *Geometric mean*.

c *Number of SNPs per Lyve-SET SNP, averaged across 12 outbreaks. For wgMLST, this is the number of alleles per Lyve-SET SNP*.

d *The average for 12 outbreaks. First value is for all data points; second value is for distances between only outbreak-associated genomes*.

e *The average for 12 outbreaks. Percentage of the reference genome included for analysis. For wgMLST, the average percentage was calculated by obtaining each GenBank-formatted file with annotated wgMLST loci and calculating the breadth of coverage for all loci*.

*More information can be found in Data Sheets 3, 4*.

**Figure 2:**
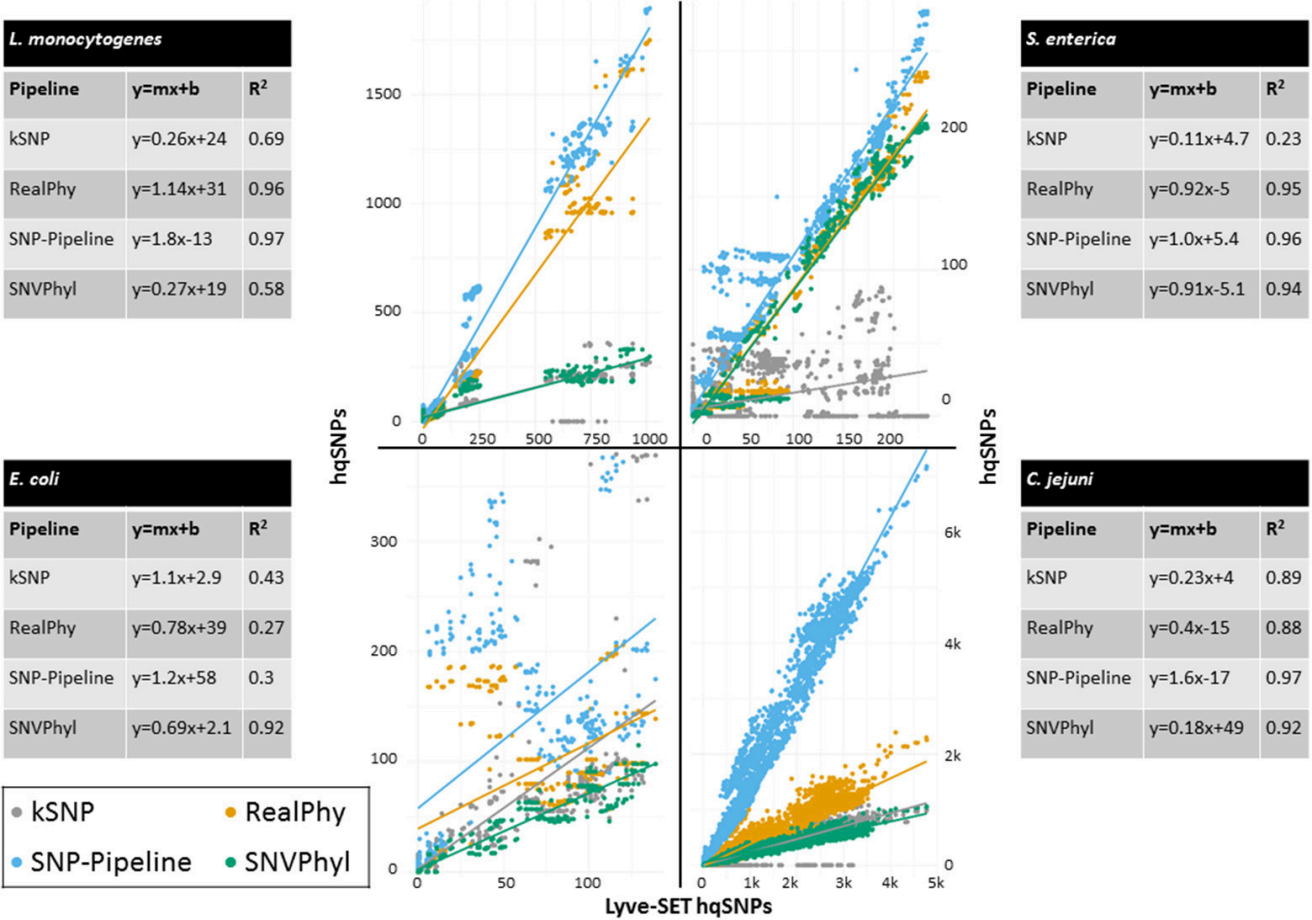
**Scatterplot of all pairwise distances**. Regression analysis of all pipelines compared with Lyve-SET. Outbreaks are shown in clockwise order from the top-left as those caused by *L. monocytogenes, S. enterica, C. jejuni*, and *E. coli*. Pairwise distances between genomes are plotted for Lyve-SET (x-axis) and other pipelines (y-axis). For each species, three outbreaks have been combined into one scatterplot. A trend line was calculated using regression analysis, and a y = mx+b formula is displayed accordingly with the goodness-of-fit (*R*^2^) value. The y = mx+b formula describes the slope of the trendline where m is the number of hqSNPs per Lyve-SET hqSNP and b is the number of hqSNPs when there are no Lyve-SET hqSNPs. All four pipelines are compared against Lyve-SET, and each panel is a different one of the four species.

The regression analyses show that other SNP pipelines correlate strongly with Lyve-SET (Figure 2). The correlation coefficients from RealPhy and Snp-Pipeline are consistently > 0.8 for outbreaks caused by *L. monocytogenes, S. enterica*, and *C. jejuni*; SNVPhyl correlates with >0.8 for *S. enterica, E. coli* and *C. jejuni*. Only SNVPhyl has a high correlation with Lyve-SET distances for *E. coli* outbreaks (*R*^2^ = 0.92). Overall except for *C. jejuni* outbreaks (*R*^2^ = 0.89), kSNP has low correlation with Lyve-SET (*R*^2^ = 0.69, 0.23, 0.43). For many of the organism-specific outbreaks tested, viewing a correlation between outbreak-only isolates was difficult because, the range of Lyve-SET SNPs is very low (Figure S1). For the *L. monocytogenes* and *E. coli* regression analyses whose Lyve-SET SNPs range 0–43 and 0–16, respectively, only RealPhy and SNVPhyl consistently have a correlation coefficient >0.8. In the *S. enterica* and *C. jejuni* analyses whose Lyve-SET distances are small, most distances from other pipelines are also small. However, there are a significant number of data points from kSNP and Snp-Pipeline in the *S. enterica* analysis whose values for Lyve-SET are zero or one, and whose distance values are >10. Additionally there are many data points in the *C. jejuni* scatter plot whose Lyve-SET distances are <3 and whose Snp-Pipeline distances are >10.

### Comparison between hqSNP and wgMLST

**Figure 3:**
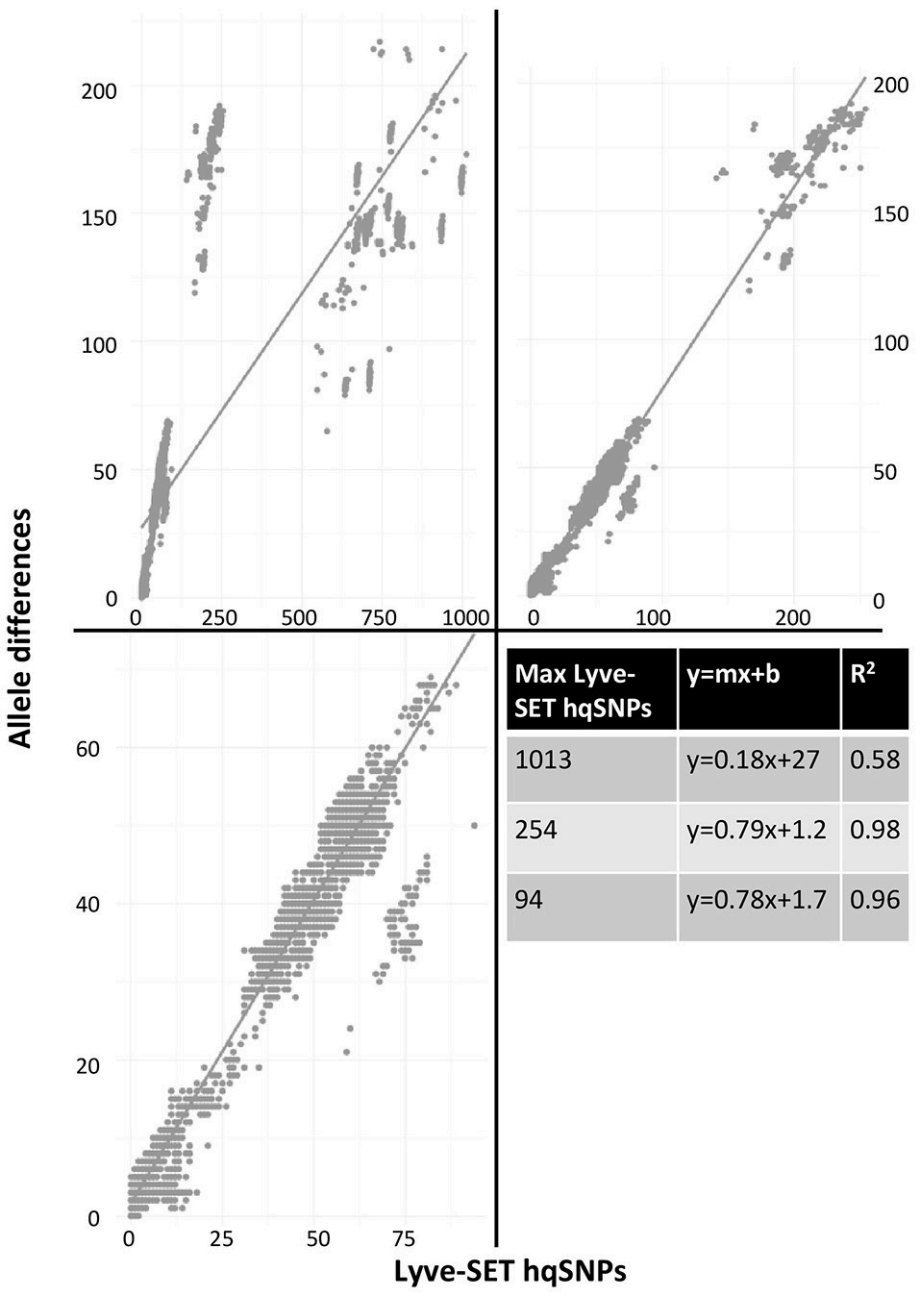
**Scatterplot of wgMLST against Lyve-SET**. As in Figure 2, a scatterplot was generated using all allelic distances from wgMLST and SNP distances from Lyve-SET, but only for the three *L. monocytogenes* outbreak clusters. The top-left plot shows all pairwise distances; the top-right limits the data points to those with <255 SNPs; the bottom-left limits the data points to those <100. For this analysis, in cluster 1408MLGX6-3WGS, PNUSAL001994 was removed as an outlier because most of its data points are zero hqSNPs in contrast to >30 alleles.

As a result of the increased utility of wgMLST for outbreak surveillance (Jackson et al., 2016), an important question is how well allelic distances compare with hqSNP distances. The only well-validated wgMLST scheme at the time of this analysis was for *L monocytogenes*; therefore, hqSNP and wgMLST comparison was performed using the *L. monocytogenes* data sets (Table 5; Moura et al., 2016). For pairwise distances found in all isolates (Figure 3, panel 1), the correlation coefficient is 0.58. However, when viewing outbreak-only distances (Figure 3, panel 3), the correlation coefficient jumps to 0.96. Visually, there are three distinct clusters of pairwise distances for *L. monocytogenes*; therefore, we performed a third regression analysis with Lyve-SET hqSNPs <255 (Figure 3, panel 2). The correlation is highest in this analysis with *R*^2^ = 0.98 and a slope of 0.79 allelic differences per Lyve-SET hqSNP.

**Table 5:** **wgMLST compared against Lyve-SET for outbreaks of *L. monocytogenes***.

	**1308MDGX6-1**	**1408MLGX6-3WGS**	**1411MLGX6-1WGS**
**PHYLOGENETIC COMPARISONS**			
*p*_Kendall–Colijn_ (λ = 0)	1E-999	1E-999	1E-4
*p*_Robinson–Foulds_	5.53E-108	1.76E-263	3.79E-71
**GENOMIC DISTANCE COMPARISONS**			
Mantel *R*^2^	0.74	0.73	0.75
Correlation coefficient	0.64	0.73	0.70
Trend line *R*^2^	0.64	0.77	0.84

The discrepancy between large and small distances and their correlations is most likely due to a large variance in the number of hqSNPs per locus. That is to say, if there are many hqSNPs, it is more likely that a single locus contains many hqSNPs and also likely that many loci contain zero or one hqSNP. To test this hypothesis, we counted the number of hqSNPs that intersected each locus in the reference genome of each of the three outbreak datasets. Fourteen percent of all intragenic hqSNPs shared a locus with at least one other hqSNP (Data Sheet 5).

## Discussion

We built a whole-genome SNP phylogenomics pipeline called Lyve-SET to aid in epidemiological investigations. The design of Lyve-SET was optimized for these investigations. Several features were incorporated to help retain high-quality SNPs, discard low-support SNPs, and generate highly reliable phylogenies (Table 1). For example, Lyve-SET has the ability to mask “cliffs,” regions where sequencing coverage significantly increases or decreases in a short genomic range. A cliff can be indicative of a repeat region that causes aggregation of short sequencing reads during read mapping. Similarly, other user-defined regions in a BED-formatted file can be supplied to mask unwanted sequences from SNP calling. One example is that, although phages can be useful for typing in their own right (Chen and Knabel, 2008), phage sequences should be removed from a SNP analysis because they often display different rates of mutation than bacterial core genomes. If phages appear to contribute to phylogenetic noise in an investigation, Lyve-SET can provide phage sequence identification with a script set_findPhages.pl which is based on a BLAST search against the PHAST database (Zhou et al., 2011). Another way for Lyve-SET to detect troublesome regions is to discard clustered SNPs. For most organisms, this option is preset to 5 bp, such that only one SNP per 5 bp passes the filter. Much like MLST, discarding clustered SNPs reduces noise introduced by horizontal gene transfer. This flanking distance hypothetically should approximate the average recombination cassette length (Vos and Didelot, 2009), but empirically we have found that having a low flanking distance, e.g., 5 bp, is sufficient. There are preset options to customize parameters of each Lyve-SET run for specific organisms (Table 2). For example, a 20x coverage cutoff is used for *S. enterica* while a 10x coverage cutoff is used for *L. monocytogenes*. In addition to aforementioned features, Lyve-SET, like other SNP pipelines, employs a set of commonly used SNP quality filters such as a percent consensus, a minimum specific coverage threshold, and a requirement of both forward and reverse reads. That is to say, each SNP must be supported by both forward and reverse reads, must have at least a certain number of reads covering each SNP, and must have a certain percentage of reads that agree with the base call. These filters are not only applied to each SNP but also to each position in the genome. Therefore, a SNP should be called for any genome position with a homologous locus in the reference genome, provided that it is not masked and passes all filters. As opposed to most other SNP pipelines, Lyve-SET calls all invariant positions in addition to SNPs in order to perform a rigorous comparison. Therefore in positions where a percentage of genomes have a variant site, all genomes with variant and invariant nucleotide calls can be appropriately compared using various models of evolution. All of these features and filters make Lyve-SET a high-quality SNP pipeline that results in a high-confidence phylogeny, which is often required for outbreak investigations.

Lyve-SET provides a detailed provenance for its outputs including the original Lyve-SET invocation and well-defined intermediate files (Table 1). All intermediate files are in standard file formats (e.g., BAM, VCF) and can be easily inspected with popular third-party tools (Li et al., 2009; Danecek et al., 2011; Rodelsperger et al., 2011; Milne et al., 2013; Tamura et al., 2013). In addition to these intermediate files, the output directory has multiple standardized and detailed files (e.g., FASTA, Newick, VCF). These too can be inspected with popular third-party tools (Danecek et al., 2011; Rodelsperger et al., 2011; Milne et al., 2013; Tamura et al., 2013).

Four other SNP pipelines including kSNP3, RealPhy, Snp-Pipeline, and SNVPhyl were chosen to analyze the same data sets along with Lyve-SET. Each of these pipelines has a history of application to outbreak investigation. In general, all the SNP pipelines evaluated in this study performed well by identifying outbreak isolates in each outbreak clade, yielding >99.5% sensitivity for all pipelines. Except for a few exceptions, all pipelines appropriately excluded non-outbreak-associated isolates. Most notably, the *S. enterica* outbreak 1203NYJAP-1 yielded conflicting results for all pipelines in varying degrees. RealPhy and kSNP identified three and five isolates, respectively, that fit into the outbreak clade for this outbreak. Snp-Pipeline excluded three isolates from the outbreak, reducing its Sn. SNVPhyl produced a star phylogeny, making it difficult to distinguish outbreak from non-outbreak. Lyve-SET included a non-outbreak-associated isolate. The outbreak 1203NYJAP-1 was the sole outbreak that reduced the specificity for Lyve-SET. In all other outbreaks, Lyve-SET correctly classified outbreak vs. non-outbreak isolates 100% of the time.

Due to the increasing utility of wgMLST and the likely co-existence of wgMLST and SNP analyses in surveillance and outbreak investigation of foodborne pathogens, we investigated the concordance of the two methods using three *L. monocytogenes* datasets. By gauging pairwise allelic distances among isolates, we found that the two methods were most consistent with each other when the number of Lyve-SET hqSNPs between any two genomes was <255. The discrepancy between the two methods grew as isolates under study became more divergent. This divergence is most likely due to multiple hits per gene, where one MLST locus could comprise multiple hqSNPs. Therefore if the diversity of the outbreak surpasses the sensitivity of the SNP pipeline and if a wgMLST scheme is available, then a wgMLST approach is more appropriate for an outbreak investigation.

The methodology and datasets reported in this study can help evaluate different pipelines. Due to the non-standard or missing intermediate files of some pipelines and even some output files, it is difficult to compare their results. We recommend that SNP pipelines provide standardized intermediate and output files such as VCF. It is impractical to compare non-standard file formats; fortunately, all pipelines evaluated in this study output a standard Newick tree file and either a FASTA or VCF file which can be used to determine genome distance. We have demonstrated some methods for comparing trees including (1) examining whether individual pipelines could identify outbreak-associated isolates consistent with previous investigation, and (2) whether two trees were significantly similar to each other using the Kendall-Colijn and Robinson-Foulds metrics. We have identified several methods for comparing genome distances. Most notably, the regression analysis has helped identify whether genome distances correlate well between two pipelines and if so, it supplies an equation (y = mx+b) that describes how much distance in one pipeline is in another pipeline.

SNP analysis for epidemiological investigations is becoming more common and is a powerful technique (Bertels et al., 2014; Pettengill et al., 2014; Morganti et al., 2015; Bekal et al., 2016; Jackson et al., 2016; Mercante et al., 2016; Shirey et al., 2016). The methods for analysis for each SNP pipeline have many nuances and are difficult to encompass into a standardized workflow. We have created Lyve-SET to incorporate the many steps of SNP calling into a complete pipeline. Therefore we present Lyve-SET to the community.

## Author contributions

Creation of the Lyve-SET software: LK. Selection of datasets: LK. Writing the manuscript: LK, XD, and HC. Comparison of Lyve-SET: LK. Testing Lyve-SET and bug discovery: LK, TG, DW, and HC. Literature search and expertise on phylogenetic and SNP matrix comparisons: AW. Co-authors of the original SET algorithm: AP, CS, GV, and LK. Running and maintaining SNVPhyl: AP, CS, and GV. Running wgMLST through BioNumerics: HC.

## Funding

This work was made possible through support from the Advanced Molecular Detection (AMD) Initiative at the Centers for Disease Control and Prevention. SNVPhyl development was funded by the Public Health Agency of Canada (PHAC), the Canadian Federal Government Genomics Research and Development Initiative (GRDI) Interdepartmental Shared Priority Project on Food and Water Safety, and Genome BC. The funding agencies had no role in study design, data collection and analysis, decision to publish, or preparation of the manuscript.

### Conflict of interest statement

The authors declare that the research was conducted in the absence of any commercial or financial relationships that could be construed as a potential conflict of interest.

## Supplementary material

Figure S1 **Scatterplot of all pairwise distances, restricted to outbreak isolates vs. outbreak isolates**. The data from Figure 2 was filtered to only data points that represent only outbreak isolates vs. outbreak isolates. All non-outbreak isolates have been removed. Due to the low numbers of data points and narrow ranges of SNPs, some trend lines are less reliable.

Data Sheet 1 **All NCBI trees**. Datasets were originally obtained using PulseNet outbreak codes. However, to identify likely phylogenetic relatives to each outbreak, we used trees from NCBI consisting of all of a single species. There are four trees used in this study. See Section Materials and Methods, Outbreak clusters, for more details.

Data Sheet 2 **All genomes in this study**. Genomes are grouped according to the outbreak code. Outbreak or non-outbreak status is shown under “event,” and a suggested reference genome is given. If a genome's event status is unknown, it is given a value of −1.

Data Sheet 3 **All visual results for tree and SNP comparisons**. Visualizations from Data Sheet 4 and other comparisons are displayed here.

Data Sheet 4 **All metrics results for tree and SNP comparisons**. All metrics that were determined from comparison tests are displayed in this file according to outbreak and pipeline. The last tab shows how Table 4 was determined.

Data Sheet 5 **hqSNPs per wgMLST locus**. MLST-annotated GenBank files were obtained from BioNumerics from each of the three *L. monocytogenes* outbreak datasets. Lyve-SET was re-run using these as reference genome assemblies. The hqSNPs in each sample was compared against the coordinates of the wgMLST loci. The number of hqSNPs per locus was counted per genome, and these counts were aggregated across outbreaks.
