# Supplementary material for: A Comparative Analysis of the Lyve-SET Phylogenomics Pipeline for Genomic Epidemiology of Foodborne Pathogens
Source: Front Microbiol. 2017 Mar 13;8:375. doi: 10.3389/fmicb.2017.00375 (PMC5346554; doi:10.3389/fmicb.2017.00375)
Supplement: Figure S1 — Scatterplot of all pairwise distances, restricted to outbreak isolates vs. outbreak isolates. The data from Figure 2 was filtered to only data points that represent only outbreak isolates vs. outbreak isolates. All non-outbreak isolates have been removed. Due to the low numbers of data points and narrow ranges of SNPs, some trend lines are less reliable. [file Image1.pdf]

L. monocytogenes		
**Pipeline**	**y=mx+b**	**R^2**
kSNP	y=0.86x-0.49	0.87
RealPhy	y=0.72x+0.24	0.89
SNP-Pipeline	y=1.0x+8.0	0.50
SNVPhyl	y=0.69x-0.063	0.88

S. enterica		
**Pipeline**	**y=mx+b**	**R^2**
kSNP	y=0.29x+2.1	0.0011
RealPhy	y=0.33x+0.0080	0.28
SNP-Pipeline	y=0.64x+0.98	0.048
SNVPhyl	y=0.18x+0.0052	0.14

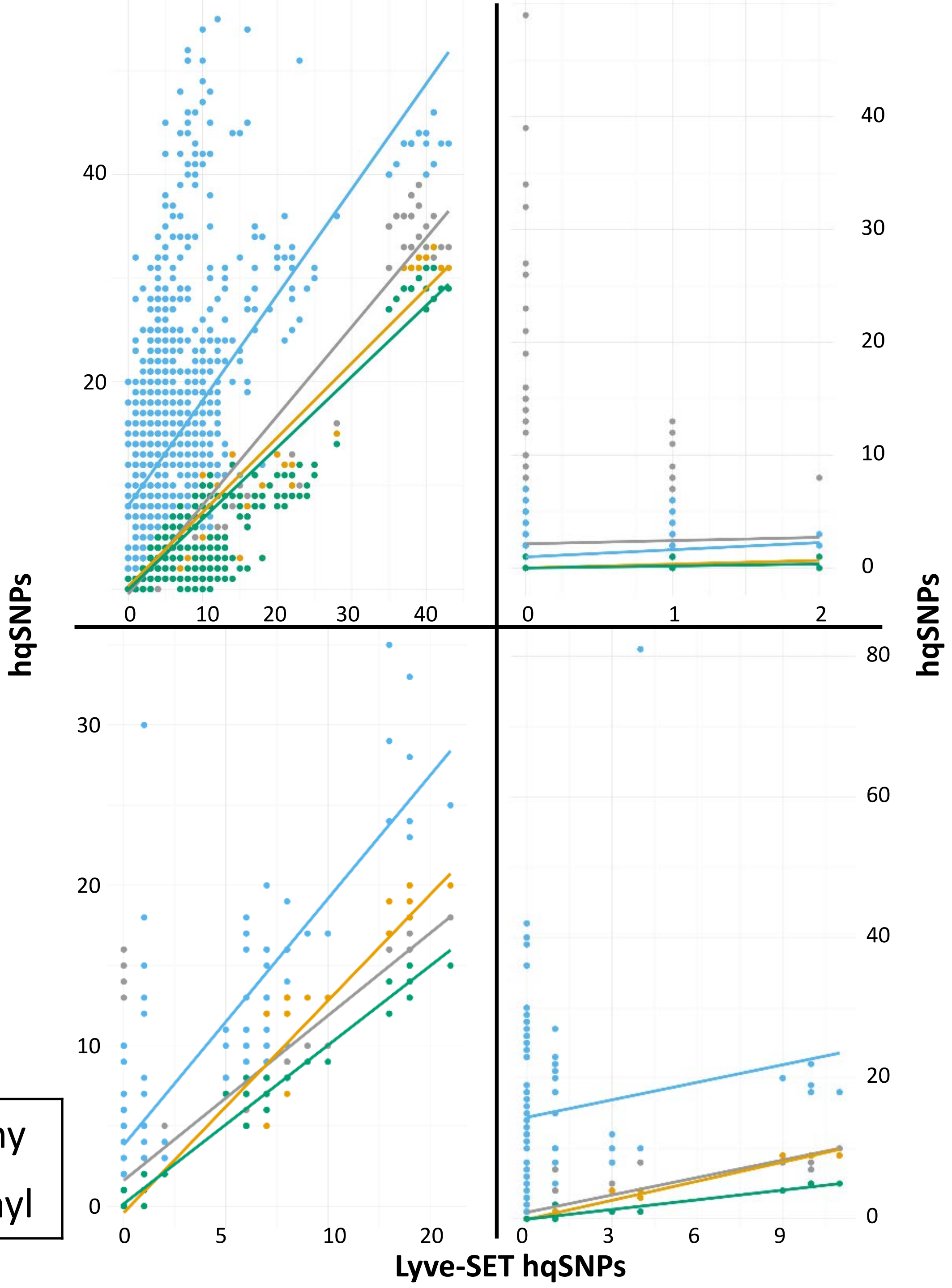

E. coli		
**Pipeline**	**y=mx+b**	**R^2**
kSNP	y=1.0x+1.6	0.71
RealPhy	y=1.3x-0.41	0.94
SNP-Pipeline	y=1.5x+3.8	0.70
SNVPhyl	y=0.99x+0.15	0.98

C. jejuni		
**Pipeline**	**y=mx+b**	**R^2**
kSNP	y=0.83x+0.90	0.64
RealPhy	y=0.91x-0.097	0.96
SNP-Pipeline	y=0.83x+14	0.022
SNVPhyl	y=0.46x-0.067	0.93
