# Supplementary material for: A Comparative Analysis of the Lyve-SET Phylogenomics Pipeline for Genomic Epidemiology of Foodborne Pathogens
Source: Front Microbiol. 2017 Mar 13;8:375. doi: 10.3389/fmicb.2017.00375 (PMC5346554; doi:10.3389/fmicb.2017.00375)
Supplement: Data Sheet 3 — All visual results for tree and SNP comparisons. Visualizations from Data Sheet 4 and other comparisons are displayed here. [file DataSheet3.zip › Presentation 3.PPTX]

## Slide 1
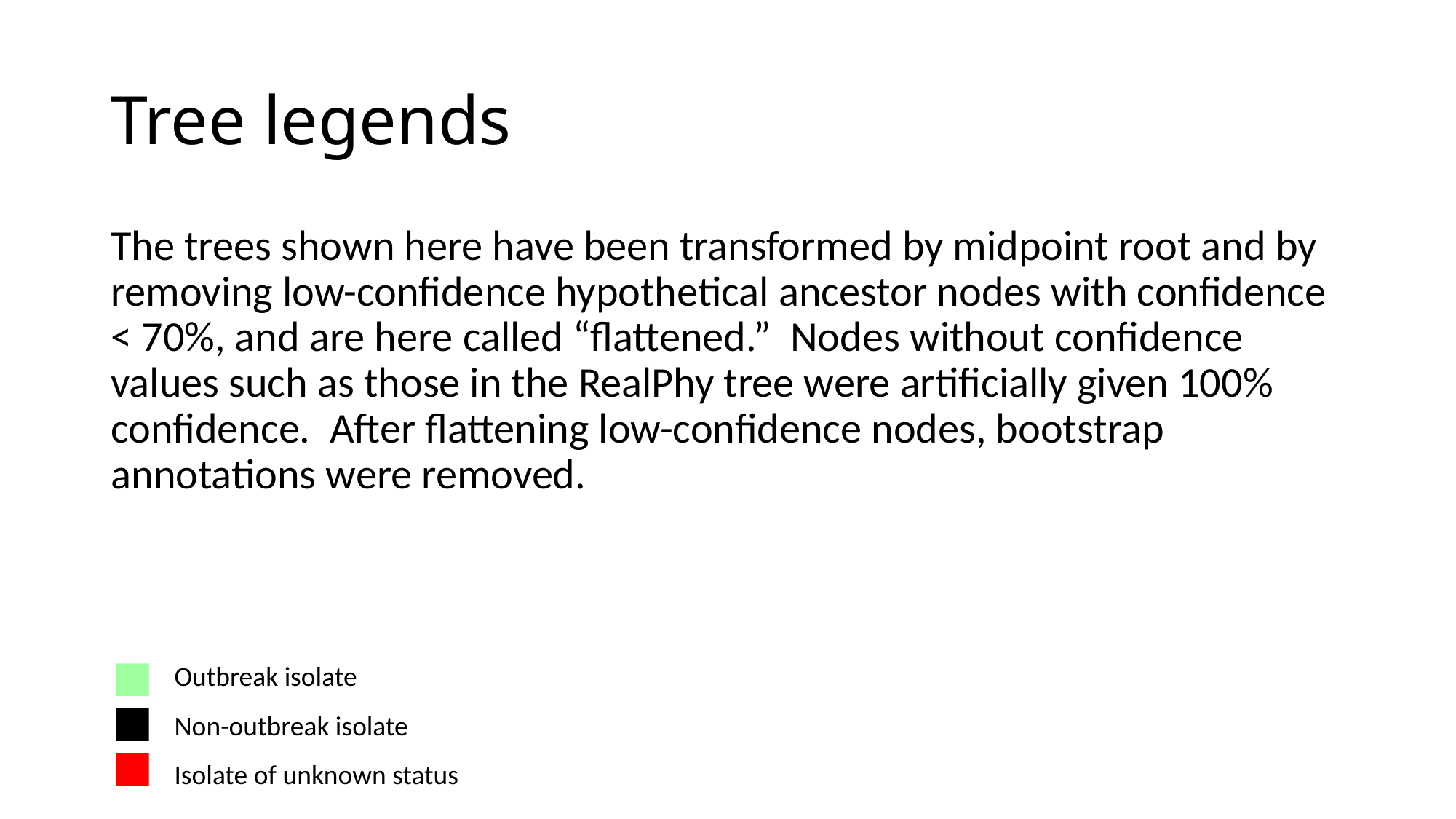

# Tree legends
The trees shown here have been transformed by midpoint root and by removing low-confidence hypothetical ancestor nodes with confidence < 70%, and are here called “flattened.” Nodes without confidence values such as those in the RealPhy tree were artificially given 100% confidence. After flattening low-confidence nodes, bootstrap annotations were removed.
Outbreak isolate
Non-outbreak isolate
Isolate of unknown status

## Slide 2
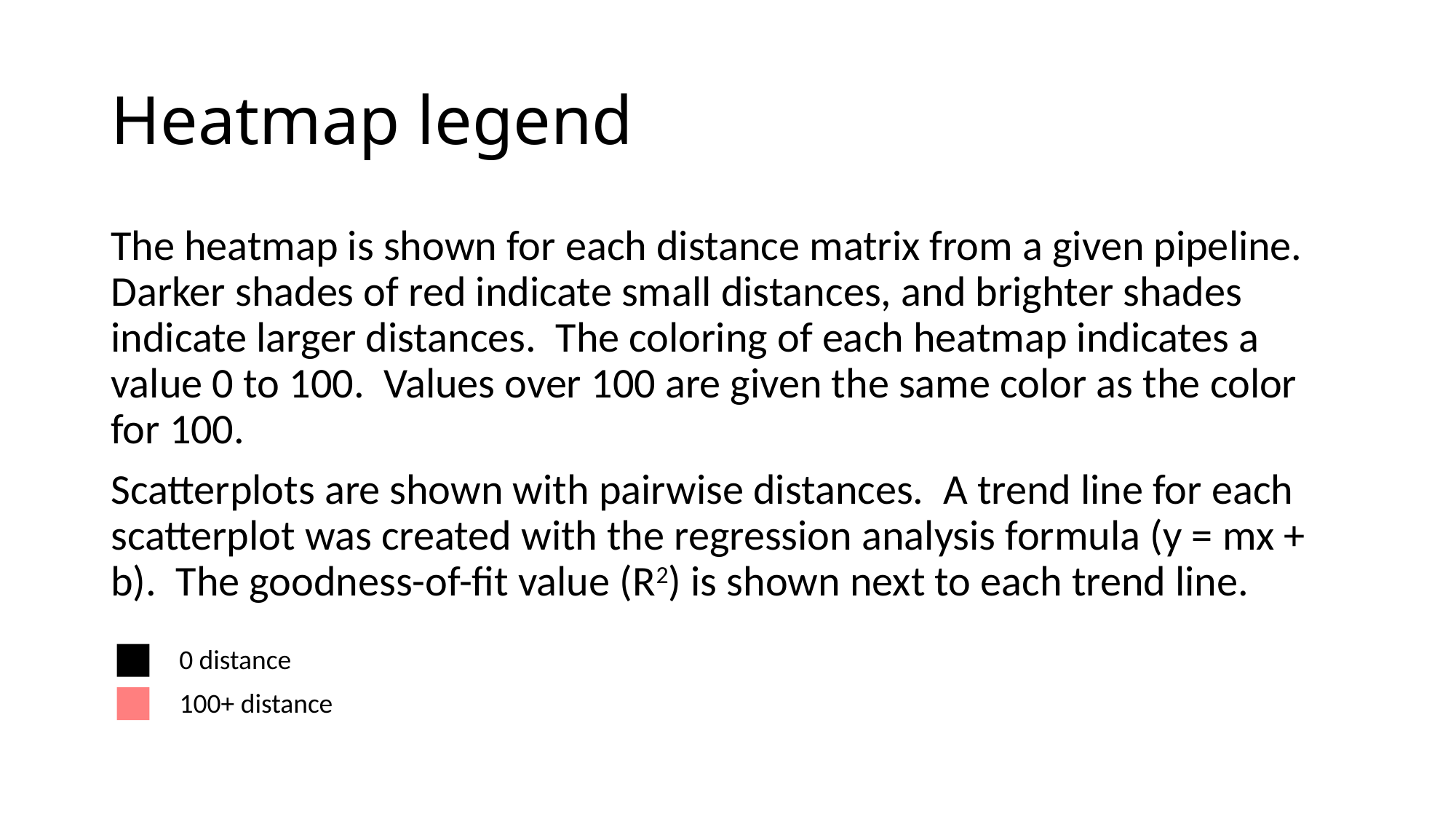

# Heatmap legend
The heatmap is shown for each distance matrix from a given pipeline. Darker shades of red indicate small distances, and brighter shades indicate larger distances. The coloring of each heatmap indicates a value 0 to 100. Values over 100 are given the same color as the color for 100.
Scatterplots are shown with pairwise distances. A trend line for each scatterplot was created with the regression analysis formula (y = mx + b). The goodness-of-fit value (R2) is shown next to each trend line.
0 distance
100+ distance

## Slide 3
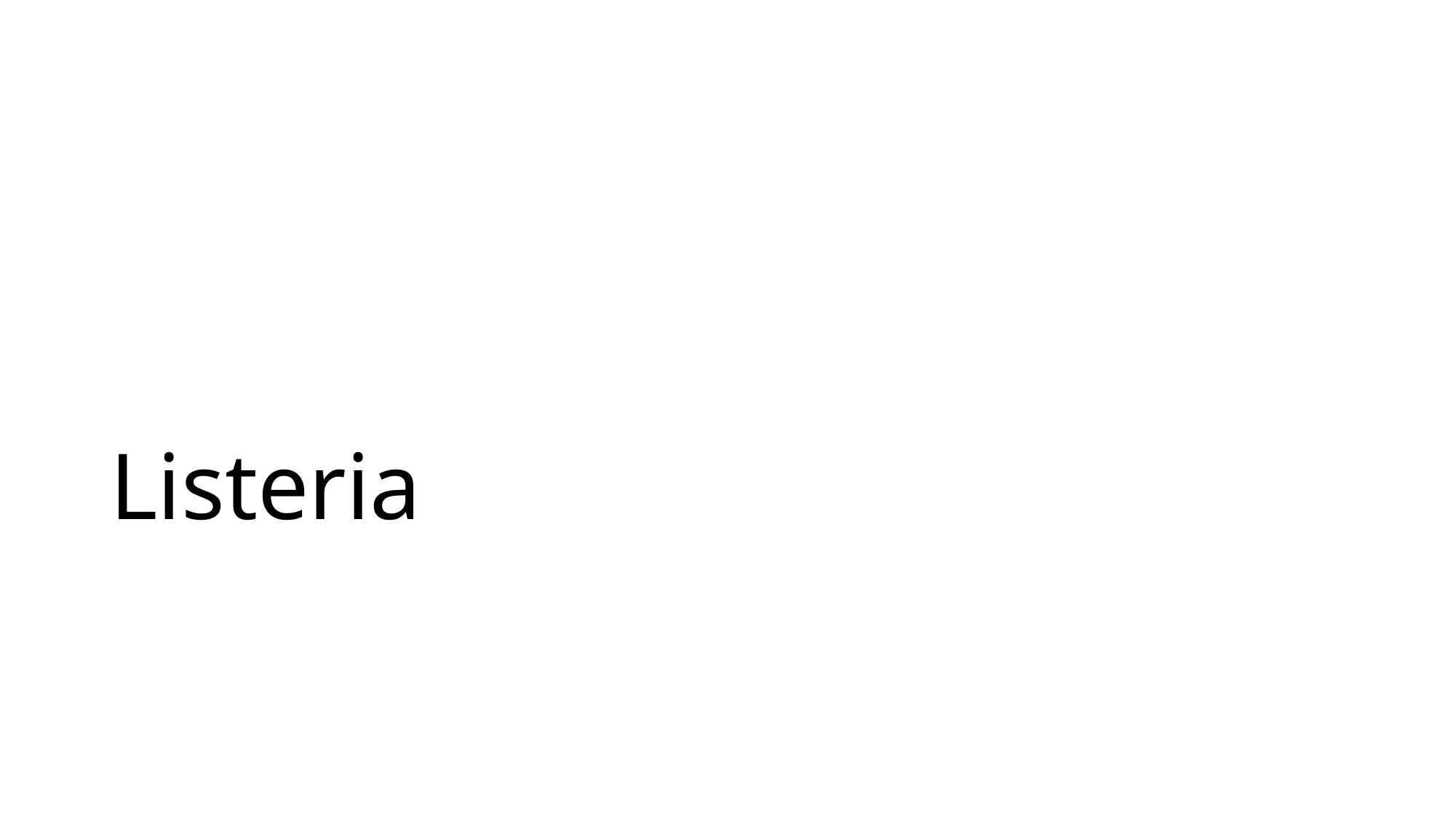

# Listeria

## Slide 4
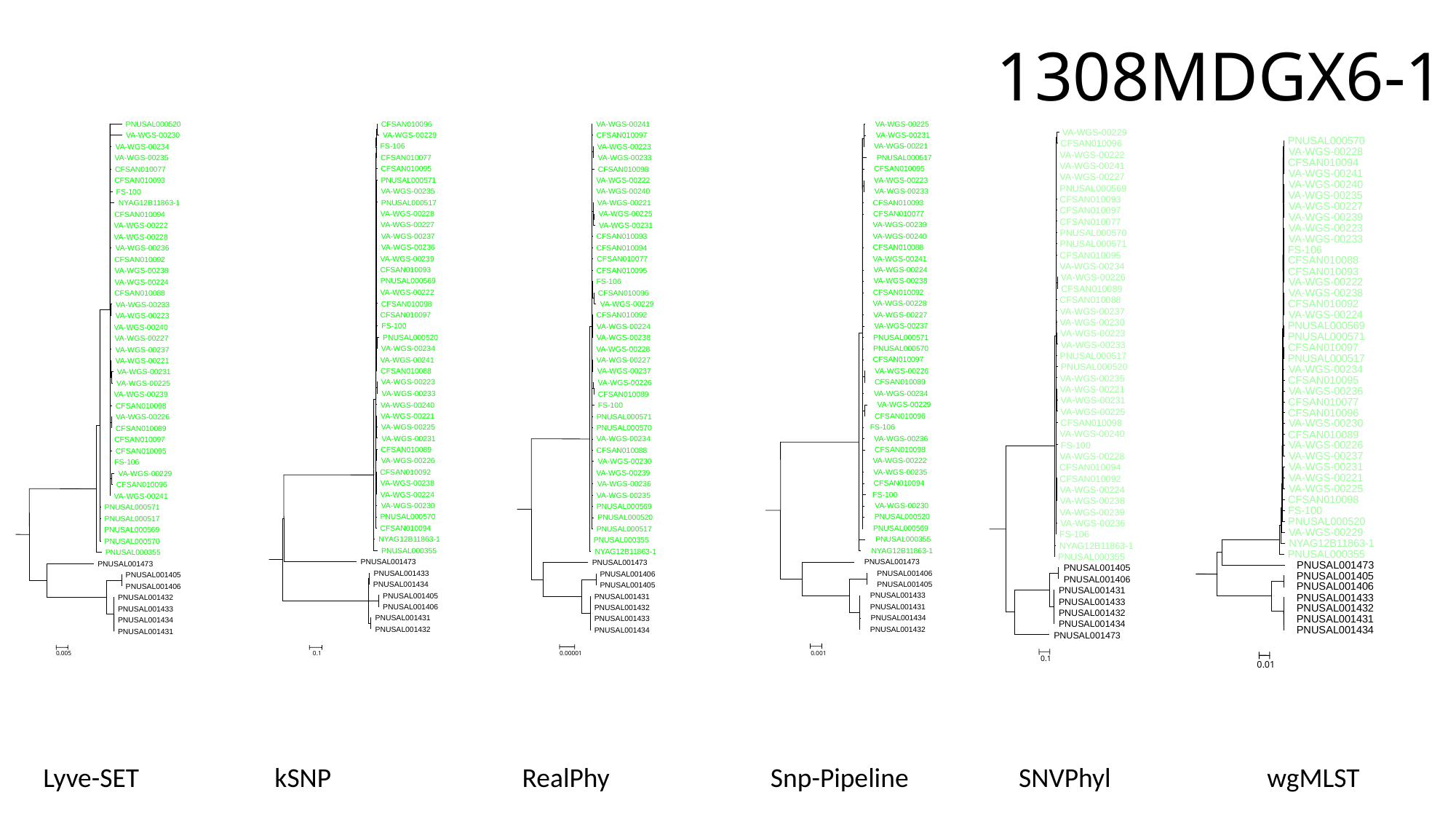

# 1308MDGX6-1
 PNUSAL000520
 VA-WGS-00230
 VA-WGS-00234
 VA-WGS-00235
 CFSAN010077
 CFSAN010093
 FS-100
 NYAG12B11863-1
 CFSAN010094
 VA-WGS-00222
 VA-WGS-00228
 VA-WGS-00236
 CFSAN010092
 VA-WGS-00238
 VA-WGS-00224
 CFSAN010088
 VA-WGS-00233
 VA-WGS-00223
 VA-WGS-00240
 VA-WGS-00227
 VA-WGS-00237
 VA-WGS-00221
 VA-WGS-00231
 VA-WGS-00225
 VA-WGS-00239
 CFSAN010098
 VA-WGS-00226
 CFSAN010089
 CFSAN010097
 CFSAN010095
 FS-106
 VA-WGS-00229
 CFSAN010096
 VA-WGS-00241
 PNUSAL000571
 PNUSAL000517
 PNUSAL000569
 PNUSAL000570
 PNUSAL000355
 PNUSAL001473
 PNUSAL001405
 PNUSAL001406
 PNUSAL001432
 PNUSAL001433
 PNUSAL001434
 PNUSAL001431
0.005
 CFSAN010096
 VA-WGS-00229
 FS-106
 CFSAN010077
 CFSAN010095
 PNUSAL000571
 VA-WGS-00235
 PNUSAL000517
 VA-WGS-00228
 VA-WGS-00227
 VA-WGS-00237
 VA-WGS-00236
 VA-WGS-00239
 CFSAN010093
 PNUSAL000569
 VA-WGS-00222
 CFSAN010098
 CFSAN010097
 FS-100
 PNUSAL000520
 VA-WGS-00234
 VA-WGS-00241
 CFSAN010088
 VA-WGS-00223
 VA-WGS-00233
 VA-WGS-00240
 VA-WGS-00221
 VA-WGS-00225
 VA-WGS-00231
 CFSAN010089
 VA-WGS-00226
 CFSAN010092
 VA-WGS-00238
 VA-WGS-00224
 VA-WGS-00230
 PNUSAL000570
 CFSAN010094
 NYAG12B11863-1
 PNUSAL000355
 PNUSAL001473
 PNUSAL001433
 PNUSAL001434
 PNUSAL001405
 PNUSAL001406
 PNUSAL001431
 PNUSAL001432
0.1
 VA-WGS-00241
 CFSAN010097
 VA-WGS-00223
 VA-WGS-00233
 CFSAN010098
 VA-WGS-00222
 VA-WGS-00240
 VA-WGS-00221
 VA-WGS-00225
 VA-WGS-00231
 CFSAN010093
 CFSAN010094
 CFSAN010077
 CFSAN010095
 FS-106
 CFSAN010096
 VA-WGS-00229
 CFSAN010092
 VA-WGS-00224
 VA-WGS-00238
 VA-WGS-00228
 VA-WGS-00227
 VA-WGS-00237
 VA-WGS-00226
 CFSAN010089
 FS-100
 PNUSAL000571
 PNUSAL000570
 VA-WGS-00234
 CFSAN010088
 VA-WGS-00230
 VA-WGS-00239
 VA-WGS-00236
 VA-WGS-00235
 PNUSAL000569
 PNUSAL000520
 PNUSAL000517
 PNUSAL000355
 NYAG12B11863-1
 PNUSAL001473
 PNUSAL001406
 PNUSAL001405
 PNUSAL001431
 PNUSAL001432
 PNUSAL001433
 PNUSAL001434
0.00001
 VA-WGS-00225
 VA-WGS-00231
 VA-WGS-00221
 PNUSAL000517
 CFSAN010095
 VA-WGS-00223
 VA-WGS-00233
 CFSAN010093
 CFSAN010077
 VA-WGS-00239
 VA-WGS-00240
 CFSAN010088
 VA-WGS-00241
 VA-WGS-00224
 VA-WGS-00238
 CFSAN010092
 VA-WGS-00228
 VA-WGS-00227
 VA-WGS-00237
 PNUSAL000571
 PNUSAL000570
 CFSAN010097
 VA-WGS-00226
 CFSAN010089
 VA-WGS-00234
 VA-WGS-00229
 CFSAN010096
 FS-106
 VA-WGS-00236
 CFSAN010098
 VA-WGS-00222
 VA-WGS-00235
 CFSAN010094
 FS-100
 VA-WGS-00230
 PNUSAL000520
 PNUSAL000569
 PNUSAL000355
 NYAG12B11863-1
 PNUSAL001473
 PNUSAL001406
 PNUSAL001405
 PNUSAL001433
 PNUSAL001431
 PNUSAL001434
 PNUSAL001432
0.001
 VA-WGS-00229
 PNUSAL000570
 CFSAN010096
 VA-WGS-00228
 VA-WGS-00222
 CFSAN010094
 VA-WGS-00241
 VA-WGS-00241
 VA-WGS-00227
 VA-WGS-00240
 PNUSAL000569
 VA-WGS-00235
 CFSAN010093
 VA-WGS-00227
 CFSAN010097
 VA-WGS-00239
 CFSAN010077
 VA-WGS-00223
 PNUSAL000570
 VA-WGS-00233
 PNUSAL000571
 FS-106
 CFSAN010095
 CFSAN010088
 VA-WGS-00234
 CFSAN010093
 VA-WGS-00226
 VA-WGS-00222
 CFSAN010089
 VA-WGS-00238
 CFSAN010088
 CFSAN010092
 VA-WGS-00237
 VA-WGS-00224
 VA-WGS-00230
 PNUSAL000569
 VA-WGS-00223
 PNUSAL000571
 VA-WGS-00233
 CFSAN010097
 PNUSAL000517
 PNUSAL000517
 PNUSAL000520
 VA-WGS-00234
 VA-WGS-00235
 CFSAN010095
 VA-WGS-00221
 VA-WGS-00236
 VA-WGS-00231
 CFSAN010077
 VA-WGS-00225
 CFSAN010096
 VA-WGS-00230
 CFSAN010098
 CFSAN010089
 VA-WGS-00240
 VA-WGS-00226
 FS-100
 VA-WGS-00237
 VA-WGS-00228
 VA-WGS-00231
 CFSAN010094
 VA-WGS-00221
 CFSAN010092
 VA-WGS-00225
 VA-WGS-00224
 CFSAN010098
 VA-WGS-00238
 FS-100
 VA-WGS-00239
 PNUSAL000520
 VA-WGS-00236
 VA-WGS-00229
 FS-106
 NYAG12B11863-1
 NYAG12B11863-1
 PNUSAL000355
 PNUSAL000355
 PNUSAL001473
 PNUSAL001405
 PNUSAL001405
 PNUSAL001406
 PNUSAL001406
 PNUSAL001431
 PNUSAL001433
 PNUSAL001433
 PNUSAL001432
 PNUSAL001432
 PNUSAL001431
 PNUSAL001434
 PNUSAL001434
 PNUSAL001473
0.1
0.01
Lyve-SET
kSNP
RealPhy
Snp-Pipeline
SNVPhyl
wgMLST

## Slide 5
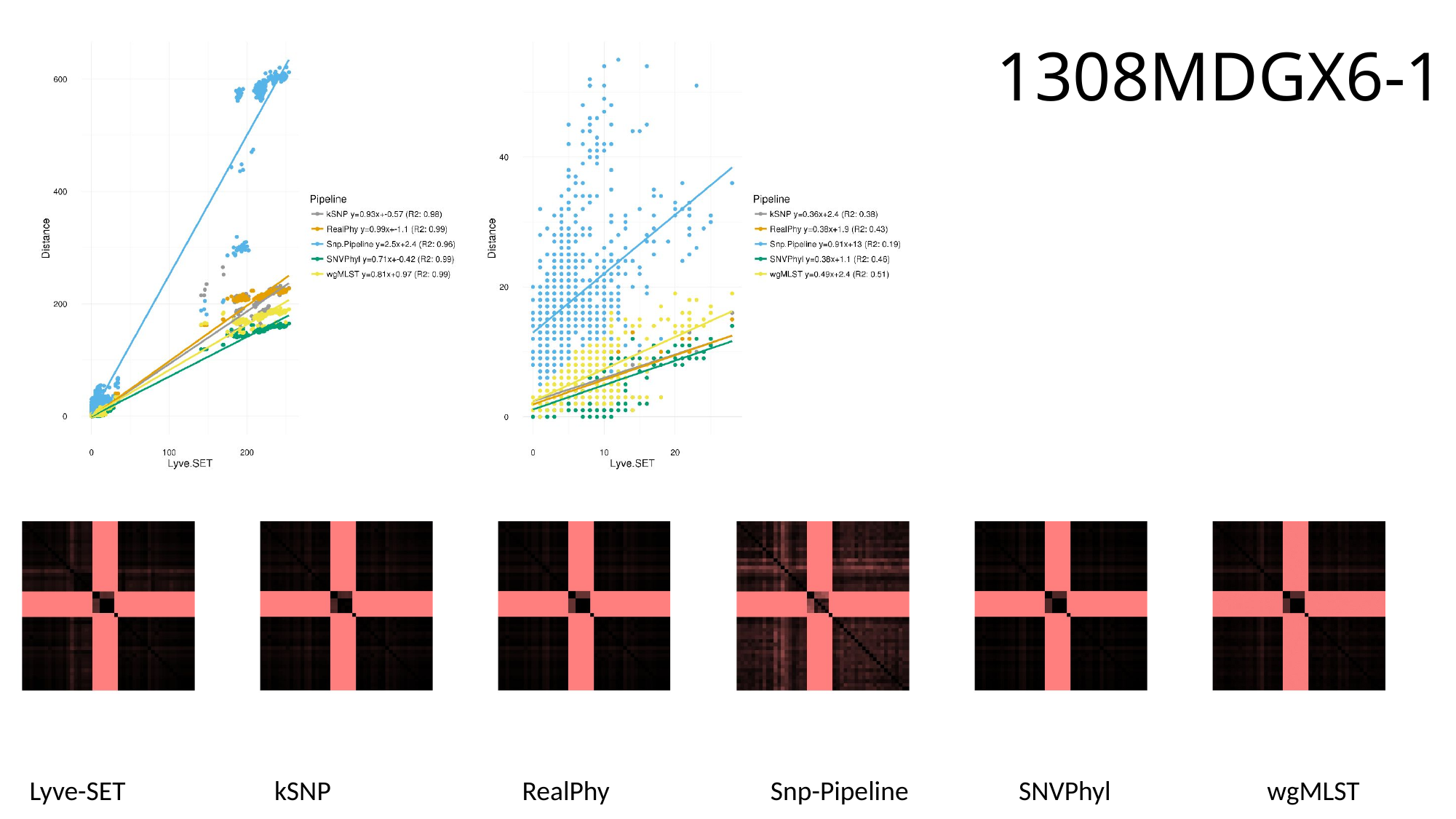

# 1308MDGX6-1
Lyve-SET
kSNP
RealPhy
Snp-Pipeline
SNVPhyl
wgMLST

## Slide 6
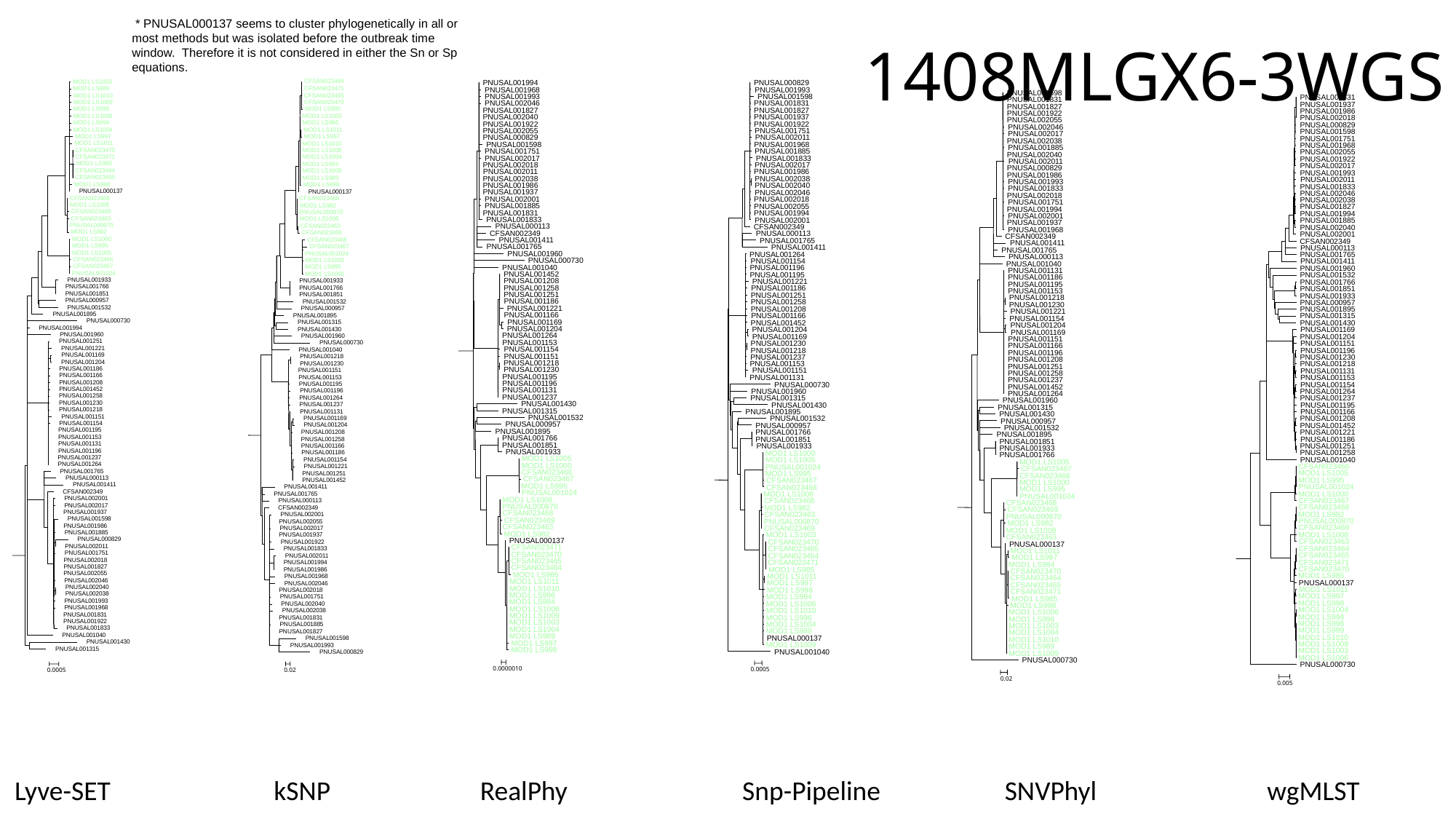

# 1408MLGX6-3WGS
 * PNUSAL000137 seems to cluster phylogenetically in all or most methods but was isolated before the outbreak time window. Therefore it is not considered in either the Sn or Sp equations.
 MOD1 LS1003
 MOD1 LS989
 MOD1 LS1010
 MOD1 LS1009
 MOD1 LS996
 MOD1 LS1006
 MOD1 LS994
 MOD1 LS1004
 MOD1 LS997
 MOD1 LS1011
 CFSAN023470
 CFSAN023471
 MOD1 LS985
 CFSAN023464
 CFSAN023465
 MOD1 LS998
 PNUSAL000137
 CFSAN023468
 MOD1 LS1008
 CFSAN023469
 CFSAN023463
 PNUSAL000870
 MOD1 LS982
 MOD1 LS1000
 MOD1 LS995
 MOD1 LS1005
 CFSAN023466
 CFSAN023467
 PNUSAL001024
 PNUSAL001933
 PNUSAL001766
 PNUSAL001851
 PNUSAL000957
 PNUSAL001532
 PNUSAL001895
 PNUSAL000730
 PNUSAL001994
 PNUSAL001960
 PNUSAL001251
 PNUSAL001221
 PNUSAL001169
 PNUSAL001204
 PNUSAL001186
 PNUSAL001166
 PNUSAL001208
 PNUSAL001452
 PNUSAL001258
 PNUSAL001230
 PNUSAL001218
 PNUSAL001151
 PNUSAL001154
 PNUSAL001195
 PNUSAL001153
 PNUSAL001131
 PNUSAL001196
 PNUSAL001237
 PNUSAL001264
 PNUSAL001765
 PNUSAL000113
 PNUSAL001411
 CFSAN002349
 PNUSAL002001
 PNUSAL002017
 PNUSAL001937
 PNUSAL001598
 PNUSAL001986
 PNUSAL001885
 PNUSAL000829
 PNUSAL002011
 PNUSAL001751
 PNUSAL002018
 PNUSAL001827
 PNUSAL002055
 PNUSAL002046
 PNUSAL002040
 PNUSAL002038
 PNUSAL001993
 PNUSAL001968
 PNUSAL001831
 PNUSAL001922
 PNUSAL001833
 PNUSAL001040
 PNUSAL001430
 PNUSAL001315
0.0005
 CFSAN023464
 CFSAN023471
 CFSAN023465
 CFSAN023470
 MOD1 LS985
 MOD1 LS1003
 MOD1 LS996
 MOD1 LS1011
 MOD1 LS997
 MOD1 LS1010
 MOD1 LS1006
 MOD1 LS1004
 MOD1 LS994
 MOD1 LS1009
 MOD1 LS989
 MOD1 LS998
 PNUSAL000137
 CFSAN023468
 MOD1 LS982
 PNUSAL000870
 MOD1 LS1008
 CFSAN023463
 CFSAN023469
 CFSAN023466
 CFSAN023467
 PNUSAL001024
 MOD1 LS1005
 MOD1 LS995
 MOD1 LS1000
 PNUSAL001933
 PNUSAL001766
 PNUSAL001851
 PNUSAL001532
 PNUSAL000957
 PNUSAL001895
 PNUSAL001315
 PNUSAL001430
 PNUSAL001960
 PNUSAL000730
 PNUSAL001040
 PNUSAL001218
 PNUSAL001230
 PNUSAL001151
 PNUSAL001153
 PNUSAL001195
 PNUSAL001196
 PNUSAL001264
 PNUSAL001237
 PNUSAL001131
 PNUSAL001169
 PNUSAL001204
 PNUSAL001208
 PNUSAL001258
 PNUSAL001166
 PNUSAL001186
 PNUSAL001154
 PNUSAL001221
 PNUSAL001251
 PNUSAL001452
 PNUSAL001411
 PNUSAL001765
 PNUSAL000113
 CFSAN002349
 PNUSAL002001
 PNUSAL002055
 PNUSAL002017
 PNUSAL001937
 PNUSAL001922
 PNUSAL001833
 PNUSAL002011
 PNUSAL001994
 PNUSAL001986
 PNUSAL001968
 PNUSAL002046
 PNUSAL002018
 PNUSAL001751
 PNUSAL002040
 PNUSAL002038
 PNUSAL001831
 PNUSAL001885
 PNUSAL001827
 PNUSAL001598
 PNUSAL001993
 PNUSAL000829
0.02
 PNUSAL001994
 PNUSAL001968
 PNUSAL001993
 PNUSAL002046
 PNUSAL001827
 PNUSAL002040
 PNUSAL001922
 PNUSAL002055
 PNUSAL000829
 PNUSAL001598
 PNUSAL001751
 PNUSAL002017
 PNUSAL002018
 PNUSAL002011
 PNUSAL002038
 PNUSAL001986
 PNUSAL001937
 PNUSAL002001
 PNUSAL001885
 PNUSAL001831
 PNUSAL001833
 PNUSAL000113
 CFSAN002349
 PNUSAL001411
 PNUSAL001765
 PNUSAL001960
 PNUSAL000730
 PNUSAL001040
 PNUSAL001452
 PNUSAL001208
 PNUSAL001258
 PNUSAL001251
 PNUSAL001186
 PNUSAL001221
 PNUSAL001166
 PNUSAL001169
 PNUSAL001204
 PNUSAL001264
 PNUSAL001153
 PNUSAL001154
 PNUSAL001151
 PNUSAL001218
 PNUSAL001230
 PNUSAL001195
 PNUSAL001196
 PNUSAL001131
 PNUSAL001237
 PNUSAL001430
 PNUSAL001315
 PNUSAL001532
 PNUSAL000957
 PNUSAL001895
 PNUSAL001766
 PNUSAL001851
 PNUSAL001933
 MOD1 LS1005
 MOD1 LS1000
 CFSAN023466
 CFSAN023467
 MOD1 LS995
 PNUSAL001024
 MOD1 LS1008
 PNUSAL000870
 CFSAN023468
 CFSAN023469
 CFSAN023463
 MOD1 LS982
 PNUSAL000137
 CFSAN023471
 CFSAN023470
 CFSAN023465
 CFSAN023464
 MOD1 LS985
 MOD1 LS1011
 MOD1 LS1010
 MOD1 LS996
 MOD1 LS994
 MOD1 LS1006
 MOD1 LS1009
 MOD1 LS1003
 MOD1 LS1004
 MOD1 LS989
 MOD1 LS997
 MOD1 LS998
0.0000010
 PNUSAL000829
 PNUSAL001993
 PNUSAL001598
 PNUSAL001831
 PNUSAL001827
 PNUSAL001937
 PNUSAL001922
 PNUSAL001751
 PNUSAL002011
 PNUSAL001968
 PNUSAL001885
 PNUSAL001833
 PNUSAL002017
 PNUSAL001986
 PNUSAL002038
 PNUSAL002040
 PNUSAL002046
 PNUSAL002018
 PNUSAL002055
 PNUSAL001994
 PNUSAL002001
 CFSAN002349
 PNUSAL000113
 PNUSAL001765
 PNUSAL001411
 PNUSAL001264
 PNUSAL001154
 PNUSAL001196
 PNUSAL001195
 PNUSAL001221
 PNUSAL001186
 PNUSAL001251
 PNUSAL001258
 PNUSAL001208
 PNUSAL001166
 PNUSAL001452
 PNUSAL001204
 PNUSAL001169
 PNUSAL001230
 PNUSAL001218
 PNUSAL001237
 PNUSAL001153
 PNUSAL001151
 PNUSAL001131
 PNUSAL000730
 PNUSAL001960
 PNUSAL001315
 PNUSAL001430
 PNUSAL001895
 PNUSAL001532
 PNUSAL000957
 PNUSAL001766
 PNUSAL001851
 PNUSAL001933
 MOD1 LS1000
 MOD1 LS1005
 PNUSAL001024
 MOD1 LS995
 CFSAN023467
 CFSAN023466
 MOD1 LS1008
 CFSAN023468
 MOD1 LS982
 CFSAN023463
 PNUSAL000870
 CFSAN023469
 MOD1 LS1003
 CFSAN023470
 CFSAN023465
 CFSAN023464
 CFSAN023471
 MOD1 LS985
 MOD1 LS1011
 MOD1 LS997
 MOD1 LS998
 MOD1 LS994
 MOD1 LS1006
 MOD1 LS1010
 MOD1 LS996
 MOD1 LS1004
 MOD1 LS989
 PNUSAL000137
 MOD1 LS1009
 PNUSAL001040
0.0005
 PNUSAL001598
 PNUSAL001831
 PNUSAL001831
 PNUSAL001937
 PNUSAL001827
 PNUSAL001986
 PNUSAL001922
 PNUSAL002018
 PNUSAL002055
 PNUSAL000829
 PNUSAL002046
 PNUSAL001598
 PNUSAL002017
 PNUSAL001751
 PNUSAL002038
 PNUSAL001968
 PNUSAL001885
 PNUSAL002055
 PNUSAL002040
 PNUSAL001922
 PNUSAL002011
 PNUSAL002017
 PNUSAL000829
 PNUSAL001993
 PNUSAL001986
 PNUSAL002011
 PNUSAL001993
 PNUSAL001833
 PNUSAL001833
 PNUSAL002046
 PNUSAL002018
 PNUSAL002038
 PNUSAL001751
 PNUSAL001827
 PNUSAL001994
 PNUSAL001994
 PNUSAL002001
 PNUSAL001885
 PNUSAL001937
 PNUSAL002040
 PNUSAL001968
 PNUSAL002001
 CFSAN002349
 CFSAN002349
 PNUSAL001411
 PNUSAL000113
 PNUSAL001765
 PNUSAL001765
 PNUSAL000113
 PNUSAL001411
 PNUSAL001040
 PNUSAL001960
 PNUSAL001131
 PNUSAL001532
 PNUSAL001186
 PNUSAL001766
 PNUSAL001195
 PNUSAL001851
 PNUSAL001153
 PNUSAL001933
 PNUSAL001218
 PNUSAL000957
 PNUSAL001230
 PNUSAL001895
 PNUSAL001221
 PNUSAL001315
 PNUSAL001154
 PNUSAL001430
 PNUSAL001204
 PNUSAL001169
 PNUSAL001169
 PNUSAL001204
 PNUSAL001151
 PNUSAL001151
 PNUSAL001166
 PNUSAL001196
 PNUSAL001196
 PNUSAL001230
 PNUSAL001208
 PNUSAL001218
 PNUSAL001251
 PNUSAL001131
 PNUSAL001258
 PNUSAL001153
 PNUSAL001237
 PNUSAL001154
 PNUSAL001452
 PNUSAL001264
 PNUSAL001264
 PNUSAL001237
 PNUSAL001960
 PNUSAL001195
 PNUSAL001315
 PNUSAL001166
 PNUSAL001430
 PNUSAL001208
 PNUSAL000957
 PNUSAL001452
 PNUSAL001532
 PNUSAL001221
 PNUSAL001895
 PNUSAL001186
 PNUSAL001851
 PNUSAL001251
 PNUSAL001933
 PNUSAL001258
 PNUSAL001766
 PNUSAL001040
 MOD1 LS1005
 CFSAN023466
 CFSAN023467
 MOD1 LS1005
 CFSAN023466
 MOD1 LS995
 MOD1 LS1000
 PNUSAL001024
 MOD1 LS995
 MOD1 LS1000
 PNUSAL001024
 CFSAN023467
 CFSAN023468
 CFSAN023468
 CFSAN023469
 MOD1 LS982
 PNUSAL000870
 PNUSAL000870
 MOD1 LS982
 CFSAN023469
 MOD1 LS1008
 MOD1 LS1008
 CFSAN023463
 CFSAN023463
 PNUSAL000137
 CFSAN023464
 MOD1 LS1011
 CFSAN023465
 MOD1 LS997
 CFSAN023471
 MOD1 LS994
 CFSAN023470
 CFSAN023470
 MOD1 LS985
 CFSAN023464
 PNUSAL000137
 CFSAN023465
 MOD1 LS1011
 CFSAN023471
 MOD1 LS997
 MOD1 LS985
 MOD1 LS998
 MOD1 LS998
 MOD1 LS1004
 MOD1 LS1006
 MOD1 LS994
 MOD1 LS996
 MOD1 LS996
 MOD1 LS1003
 MOD1 LS989
 MOD1 LS1004
 MOD1 LS1010
 MOD1 LS1010
 MOD1 LS1009
 MOD1 LS989
 MOD1 LS1003
 MOD1 LS1009
 MOD1 LS1006
 PNUSAL000730
 PNUSAL000730
0.02
0.005
Lyve-SET
kSNP
RealPhy
Snp-Pipeline
SNVPhyl
wgMLST

## Slide 7
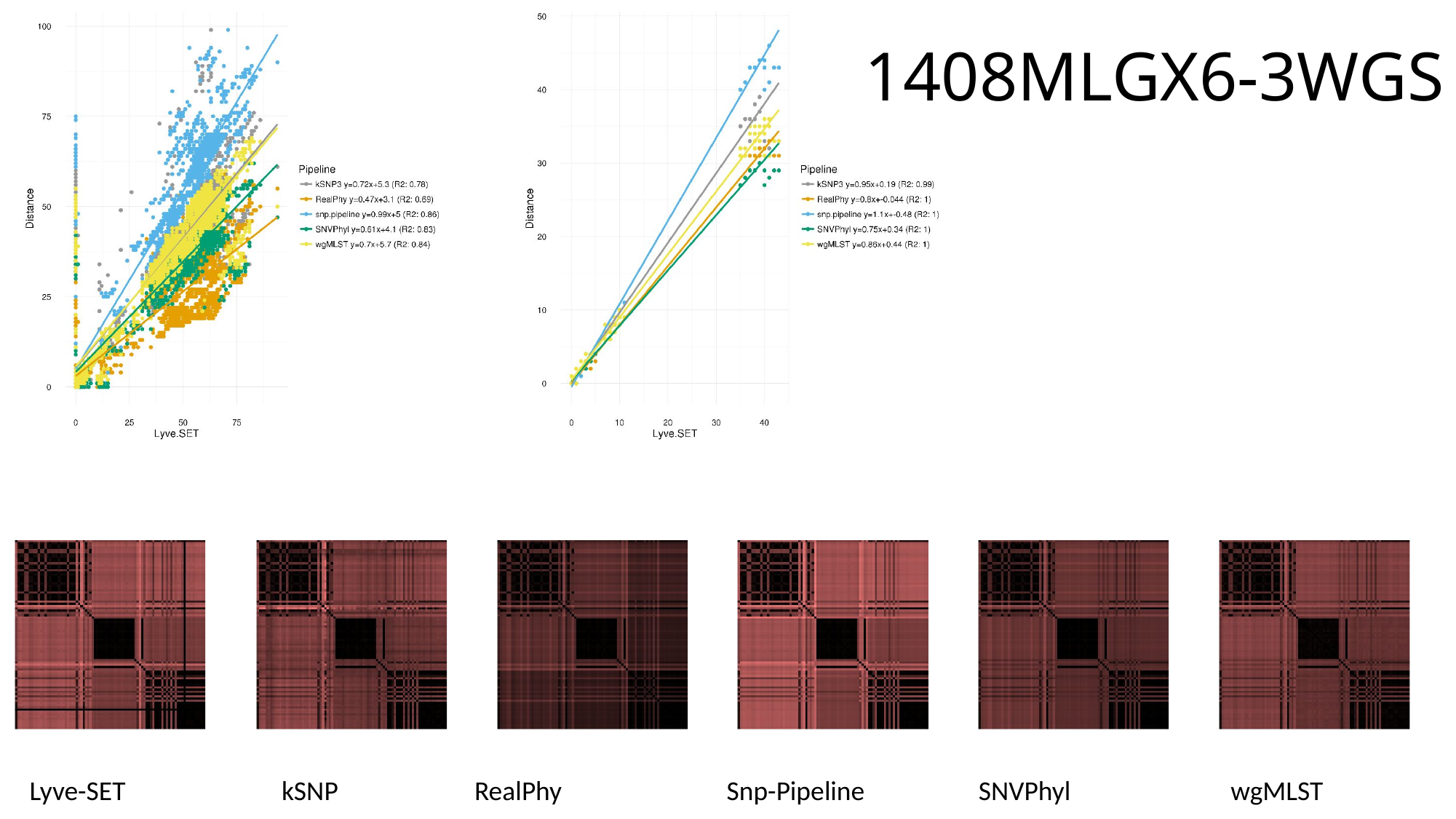

# 1408MLGX6-3WGS
Lyve-SET
kSNP
RealPhy
Snp-Pipeline
SNVPhyl
wgMLST

## Slide 8
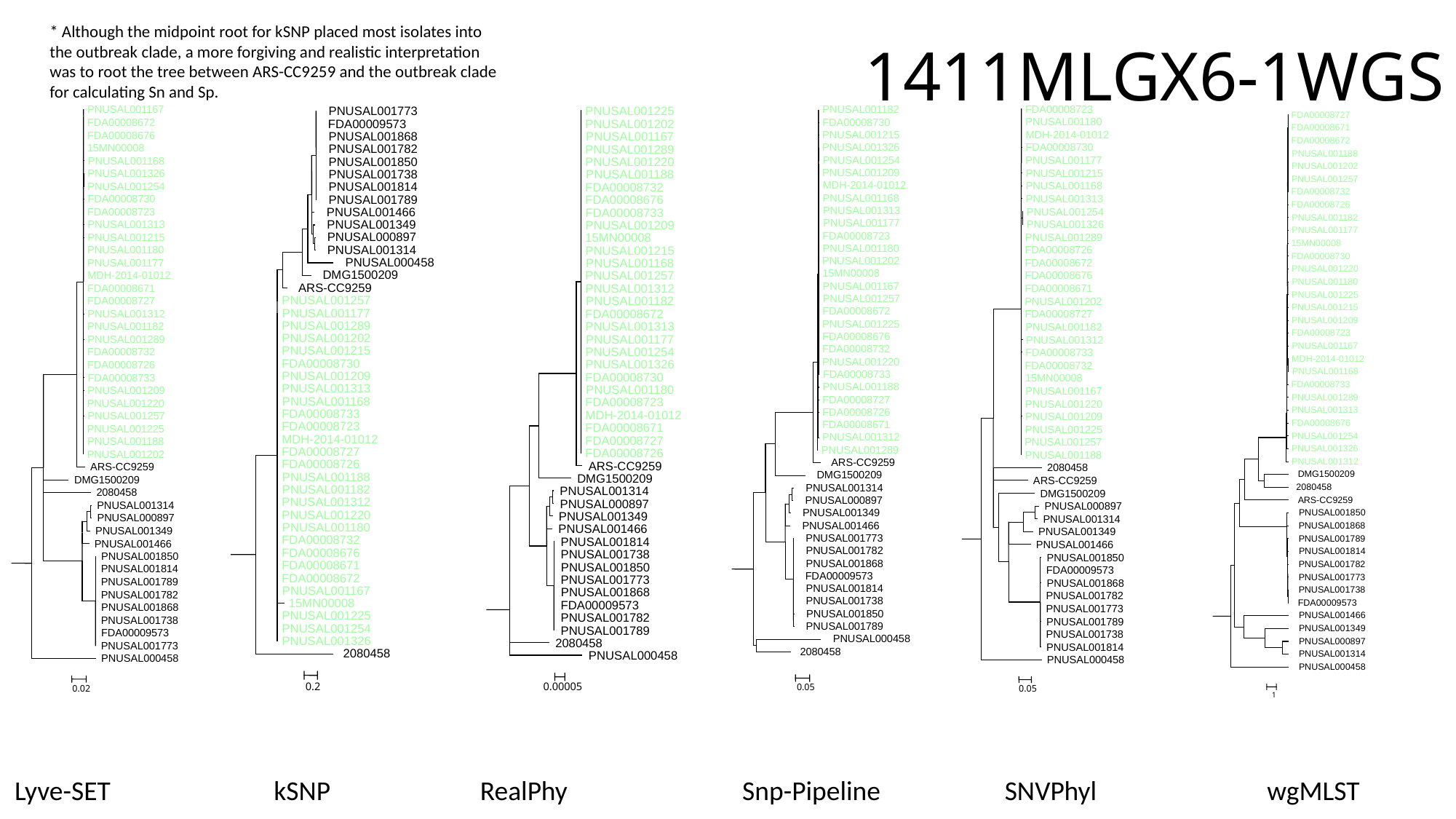

# 1411MLGX6-1WGS
* Although the midpoint root for kSNP placed most isolates into the outbreak clade, a more forgiving and realistic interpretation was to root the tree between ARS-CC9259 and the outbreak clade for calculating Sn and Sp.
 FDA00008723
 PNUSAL001180
 MDH-2014-01012
 FDA00008730
 PNUSAL001177
 PNUSAL001215
 PNUSAL001168
 PNUSAL001313
 PNUSAL001254
 PNUSAL001326
 PNUSAL001289
 FDA00008726
 FDA00008672
 FDA00008676
 FDA00008671
 PNUSAL001202
 FDA00008727
 PNUSAL001182
 PNUSAL001312
 FDA00008733
 FDA00008732
 15MN00008
 PNUSAL001167
 PNUSAL001220
 PNUSAL001209
 PNUSAL001225
 PNUSAL001257
 PNUSAL001188
 2080458
 ARS-CC9259
 DMG1500209
 PNUSAL000897
 PNUSAL001314
 PNUSAL001349
 PNUSAL001466
 PNUSAL001850
 FDA00009573
 PNUSAL001868
 PNUSAL001782
 PNUSAL001773
 PNUSAL001789
 PNUSAL001738
 PNUSAL001814
 PNUSAL000458
0.05
 PNUSAL001167
 FDA00008672
 FDA00008676
 15MN00008
 PNUSAL001168
 PNUSAL001326
 PNUSAL001254
 FDA00008730
 FDA00008723
 PNUSAL001313
 PNUSAL001215
 PNUSAL001180
 PNUSAL001177
 MDH-2014-01012
 FDA00008671
 FDA00008727
 PNUSAL001312
 PNUSAL001182
 PNUSAL001289
 FDA00008732
 FDA00008726
 FDA00008733
 PNUSAL001209
 PNUSAL001220
 PNUSAL001257
 PNUSAL001225
 PNUSAL001188
 PNUSAL001202
 ARS-CC9259
 DMG1500209
 2080458
 PNUSAL001314
 PNUSAL000897
 PNUSAL001349
 PNUSAL001466
 PNUSAL001850
 PNUSAL001814
 PNUSAL001789
 PNUSAL001782
 PNUSAL001868
 PNUSAL001738
 FDA00009573
 PNUSAL001773
 PNUSAL000458
0.02
 PNUSAL001773
 FDA00009573
 PNUSAL001868
 PNUSAL001782
 PNUSAL001850
 PNUSAL001738
 PNUSAL001814
 PNUSAL001789
 PNUSAL001466
 PNUSAL001349
 PNUSAL000897
 PNUSAL001314
 PNUSAL000458
 DMG1500209
 ARS-CC9259
 PNUSAL001257
 PNUSAL001177
 PNUSAL001289
 PNUSAL001202
 PNUSAL001215
 FDA00008730
 PNUSAL001209
 PNUSAL001313
 PNUSAL001168
 FDA00008733
 FDA00008723
 MDH-2014-01012
 FDA00008727
 FDA00008726
 PNUSAL001188
 PNUSAL001182
 PNUSAL001312
 PNUSAL001220
 PNUSAL001180
 FDA00008732
 FDA00008676
 FDA00008671
 FDA00008672
 PNUSAL001167
 15MN00008
 PNUSAL001225
 PNUSAL001254
 PNUSAL001326
 2080458
0.2
 PNUSAL001225
 PNUSAL001202
 PNUSAL001167
 PNUSAL001289
 PNUSAL001220
 PNUSAL001188
 FDA00008732
 FDA00008676
 FDA00008733
 PNUSAL001209
 15MN00008
 PNUSAL001215
 PNUSAL001168
 PNUSAL001257
 PNUSAL001312
 PNUSAL001182
 FDA00008672
 PNUSAL001313
 PNUSAL001177
 PNUSAL001254
 PNUSAL001326
 FDA00008730
 PNUSAL001180
 FDA00008723
 MDH-2014-01012
 FDA00008671
 FDA00008727
 FDA00008726
 ARS-CC9259
 DMG1500209
 PNUSAL001314
 PNUSAL000897
 PNUSAL001349
 PNUSAL001466
 PNUSAL001814
 PNUSAL001738
 PNUSAL001850
 PNUSAL001773
 PNUSAL001868
 FDA00009573
 PNUSAL001782
 PNUSAL001789
 2080458
 PNUSAL000458
0.00005
 PNUSAL001182
 FDA00008730
 PNUSAL001215
 PNUSAL001326
 PNUSAL001254
 PNUSAL001209
 MDH-2014-01012
 PNUSAL001168
 PNUSAL001313
 PNUSAL001177
 FDA00008723
 PNUSAL001180
 PNUSAL001202
 15MN00008
 PNUSAL001167
 PNUSAL001257
 FDA00008672
 PNUSAL001225
 FDA00008676
 FDA00008732
 PNUSAL001220
 FDA00008733
 PNUSAL001188
 FDA00008727
 FDA00008726
 FDA00008671
 PNUSAL001312
 PNUSAL001289
 ARS-CC9259
 DMG1500209
 PNUSAL001314
 PNUSAL000897
 PNUSAL001349
 PNUSAL001466
 PNUSAL001773
 PNUSAL001782
 PNUSAL001868
 FDA00009573
 PNUSAL001814
 PNUSAL001738
 PNUSAL001850
 PNUSAL001789
 PNUSAL000458
 2080458
0.05
 FDA00008727
 FDA00008671
 FDA00008672
 PNUSAL001188
 PNUSAL001202
 PNUSAL001257
 FDA00008732
 FDA00008726
 PNUSAL001182
 PNUSAL001177
 15MN00008
 FDA00008730
 PNUSAL001220
 PNUSAL001180
 PNUSAL001225
 PNUSAL001215
 PNUSAL001209
 FDA00008723
 PNUSAL001167
 MDH-2014-01012
 PNUSAL001168
 FDA00008733
 PNUSAL001289
 PNUSAL001313
 FDA00008676
 PNUSAL001254
 PNUSAL001326
 PNUSAL001312
 DMG1500209
 2080458
 ARS-CC9259
 PNUSAL001850
 PNUSAL001868
 PNUSAL001789
 PNUSAL001814
 PNUSAL001782
 PNUSAL001773
 PNUSAL001738
 FDA00009573
 PNUSAL001466
 PNUSAL001349
 PNUSAL000897
 PNUSAL001314
 PNUSAL000458
1
Lyve-SET
kSNP
RealPhy
Snp-Pipeline
SNVPhyl
wgMLST

## Slide 9
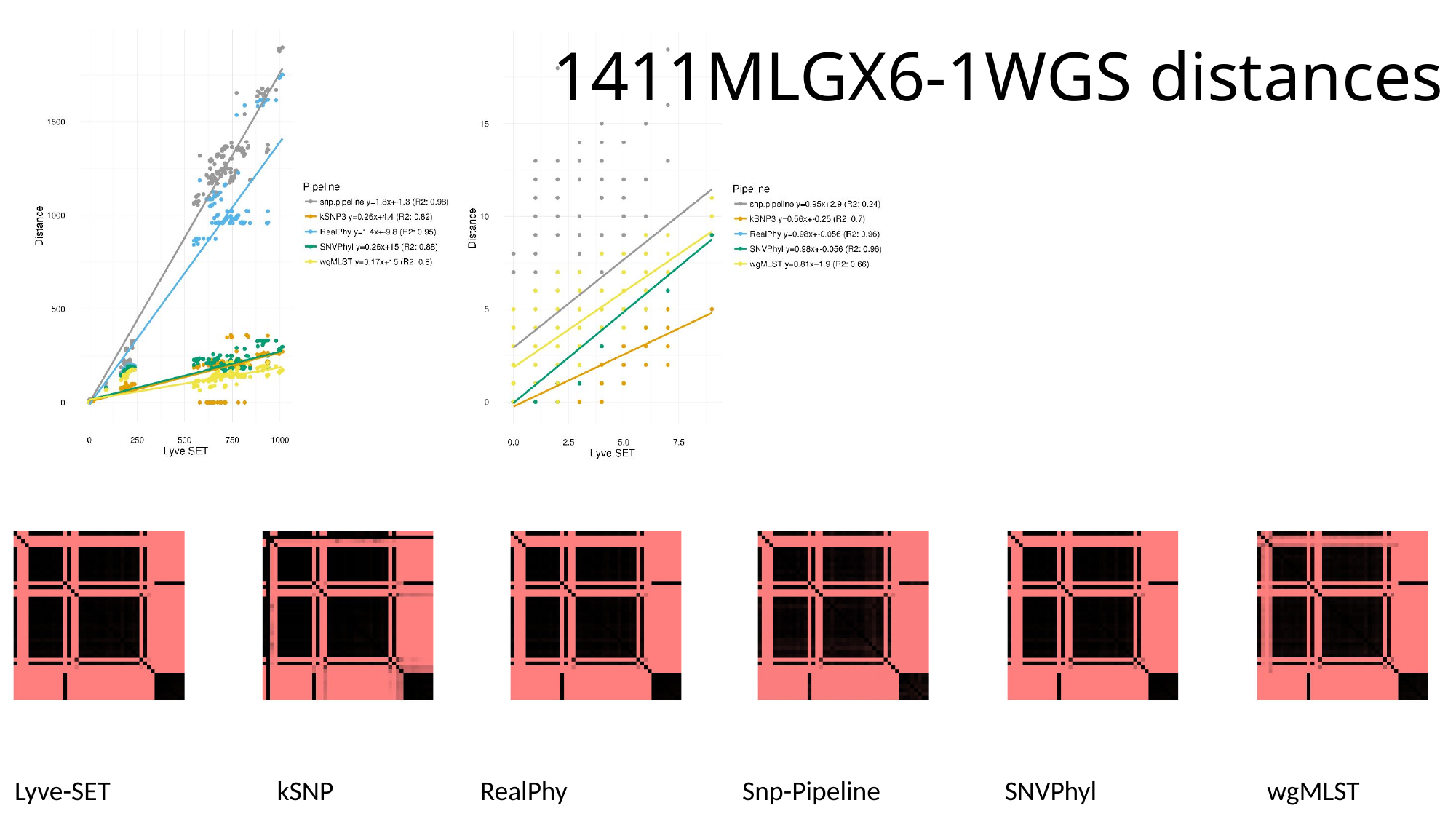

# 1411MLGX6-1WGS distances
Lyve-SET
kSNP
RealPhy
Snp-Pipeline
SNVPhyl
wgMLST

## Slide 10
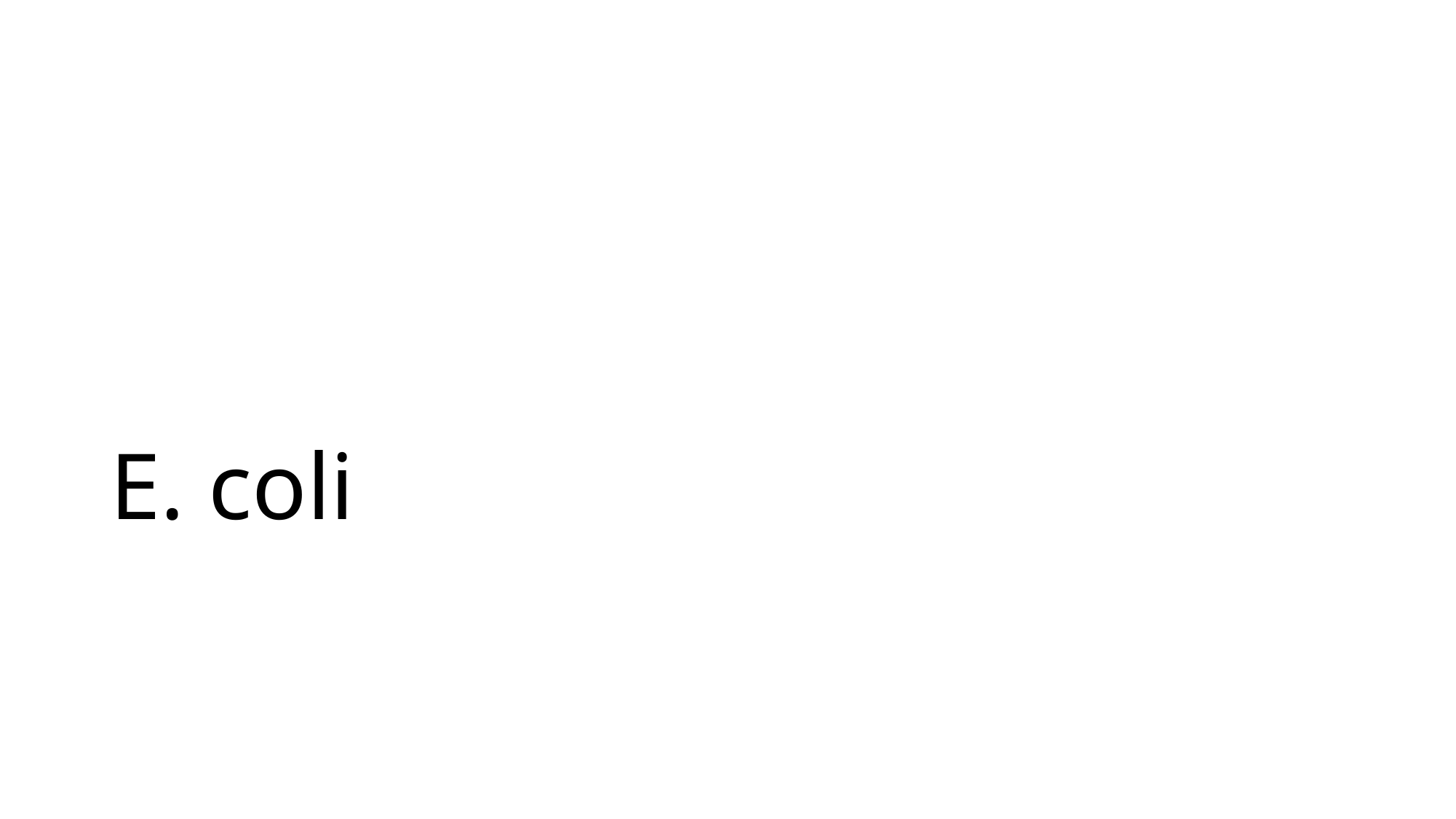

# E. coli

## Slide 11
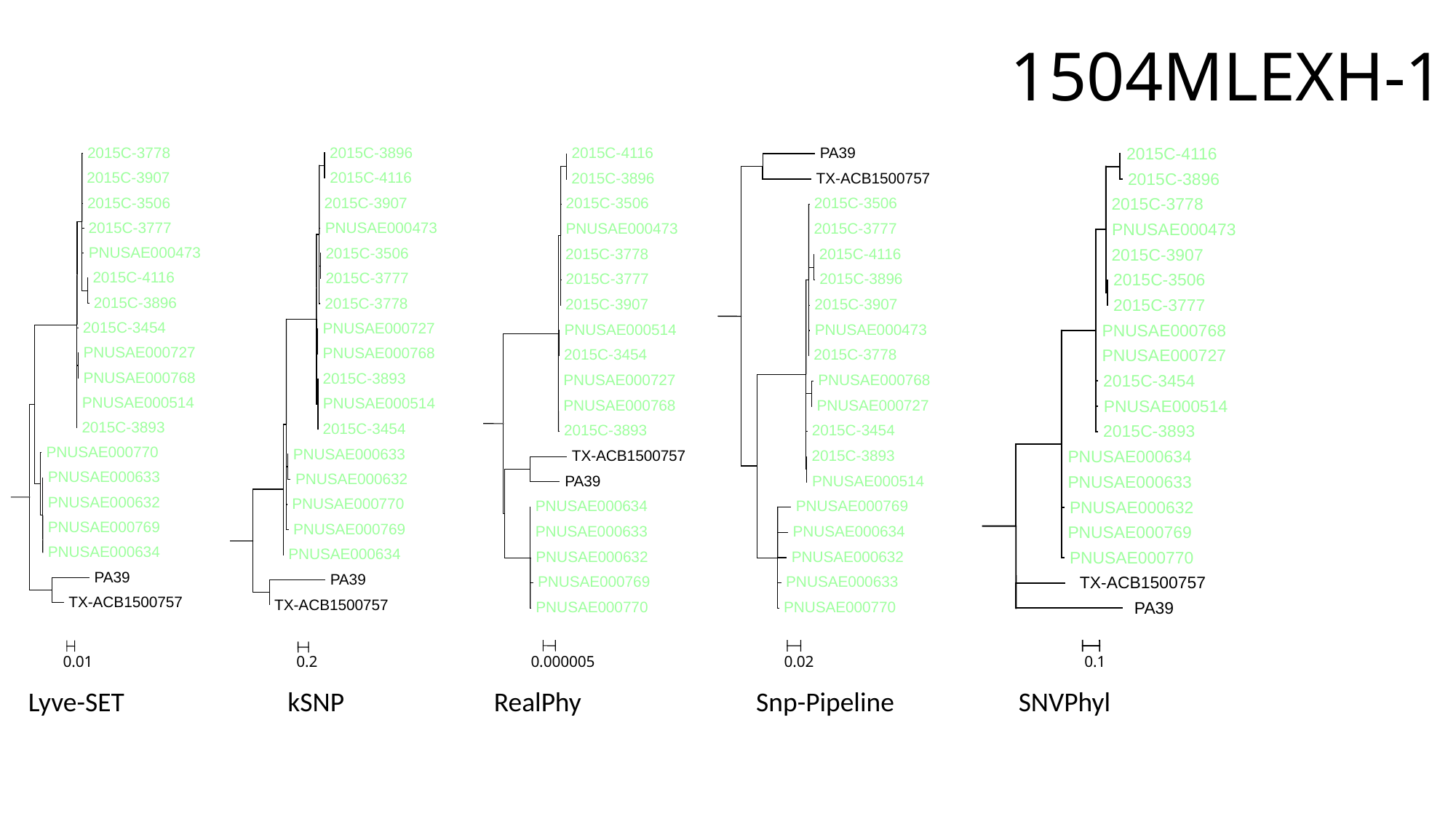

# 1504MLEXH-1
 2015C-3778
 2015C-3907
 2015C-3506
 2015C-3777
 PNUSAE000473
 2015C-4116
 2015C-3896
 2015C-3454
 PNUSAE000727
 PNUSAE000768
 PNUSAE000514
 2015C-3893
 PNUSAE000770
 PNUSAE000633
 PNUSAE000632
 PNUSAE000769
 PNUSAE000634
 PA39
 TX-ACB1500757
0.01
 2015C-3896
 2015C-4116
 2015C-3907
 PNUSAE000473
 2015C-3506
 2015C-3777
 2015C-3778
 PNUSAE000727
 PNUSAE000768
 2015C-3893
 PNUSAE000514
 2015C-3454
 PNUSAE000633
 PNUSAE000632
 PNUSAE000770
 PNUSAE000769
 PNUSAE000634
 PA39
 TX-ACB1500757
0.2
 2015C-4116
 2015C-3896
 2015C-3506
 PNUSAE000473
 2015C-3778
 2015C-3777
 2015C-3907
 PNUSAE000514
 2015C-3454
 PNUSAE000727
 PNUSAE000768
 2015C-3893
 TX-ACB1500757
 PA39
 PNUSAE000634
 PNUSAE000633
 PNUSAE000632
 PNUSAE000769
 PNUSAE000770
0.000005
 PA39
 TX-ACB1500757
 2015C-3506
 2015C-3777
 2015C-4116
 2015C-3896
 2015C-3907
 PNUSAE000473
 2015C-3778
 PNUSAE000768
 PNUSAE000727
 2015C-3454
 2015C-3893
 PNUSAE000514
 PNUSAE000769
 PNUSAE000634
 PNUSAE000632
 PNUSAE000633
 PNUSAE000770
0.02
 2015C-4116
 2015C-3896
 2015C-3778
 PNUSAE000473
 2015C-3907
 2015C-3506
 2015C-3777
 PNUSAE000768
 PNUSAE000727
 2015C-3454
 PNUSAE000514
 2015C-3893
 PNUSAE000634
 PNUSAE000633
 PNUSAE000632
 PNUSAE000769
 PNUSAE000770
 TX-ACB1500757
 PA39
0.1
Lyve-SET
kSNP
RealPhy
Snp-Pipeline
SNVPhyl

## Slide 12
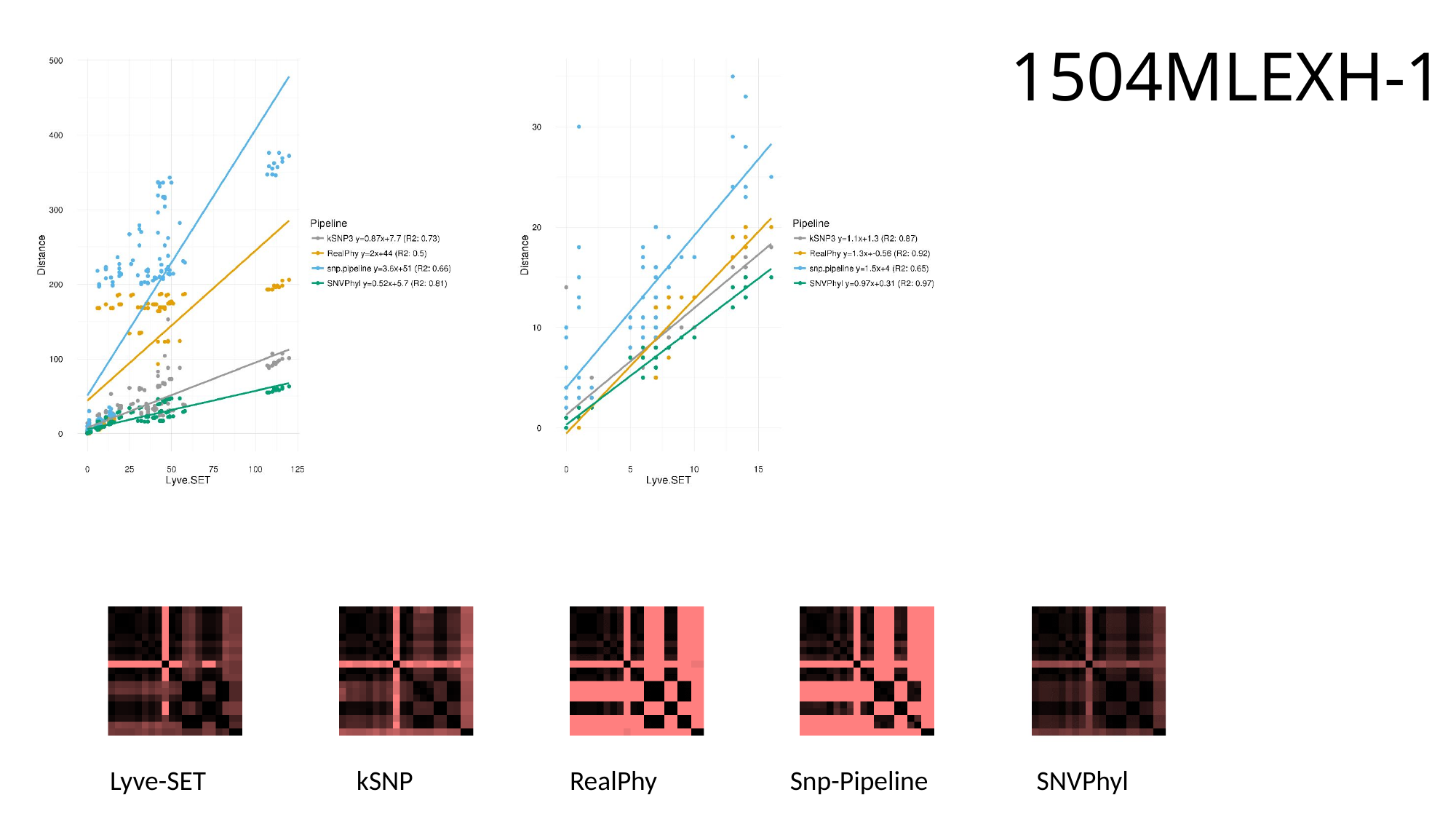

# 1504MLEXH-1
Lyve-SET
kSNP
RealPhy
Snp-Pipeline
SNVPhyl

## Slide 13
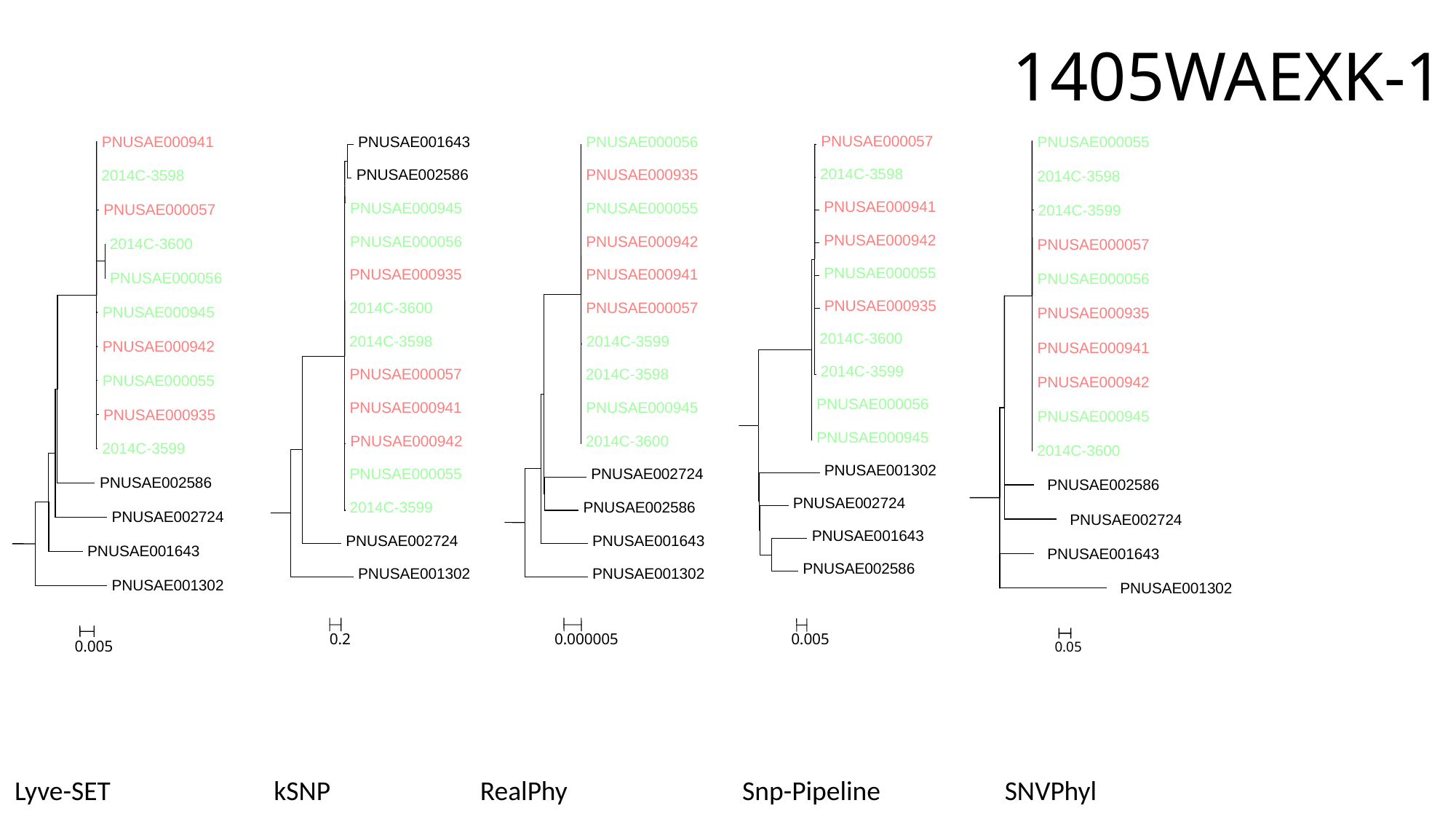

# 1405WAEXK-1
 PNUSAE000941
 2014C-3598
 PNUSAE000057
 2014C-3600
 PNUSAE000056
 PNUSAE000945
 PNUSAE000942
 PNUSAE000055
 PNUSAE000935
 2014C-3599
 PNUSAE002586
 PNUSAE002724
 PNUSAE001643
 PNUSAE001302
0.005
 PNUSAE001643
 PNUSAE002586
 PNUSAE000945
 PNUSAE000056
 PNUSAE000935
 2014C-3600
 2014C-3598
 PNUSAE000057
 PNUSAE000941
 PNUSAE000942
 PNUSAE000055
 2014C-3599
 PNUSAE002724
 PNUSAE001302
0.2
 PNUSAE000056
 PNUSAE000935
 PNUSAE000055
 PNUSAE000942
 PNUSAE000941
 PNUSAE000057
 2014C-3599
 2014C-3598
 PNUSAE000945
 2014C-3600
 PNUSAE002724
 PNUSAE002586
 PNUSAE001643
 PNUSAE001302
0.000005
 PNUSAE000057
 2014C-3598
 PNUSAE000941
 PNUSAE000942
 PNUSAE000055
 PNUSAE000935
 2014C-3600
 2014C-3599
 PNUSAE000056
 PNUSAE000945
 PNUSAE001302
 PNUSAE002724
 PNUSAE001643
 PNUSAE002586
0.005
 PNUSAE000055
 2014C-3598
 2014C-3599
 PNUSAE000057
 PNUSAE000056
 PNUSAE000935
 PNUSAE000941
 PNUSAE000942
 PNUSAE000945
 2014C-3600
 PNUSAE002586
 PNUSAE002724
 PNUSAE001643
 PNUSAE001302
0.05
Lyve-SET
kSNP
RealPhy
Snp-Pipeline
SNVPhyl

## Slide 14
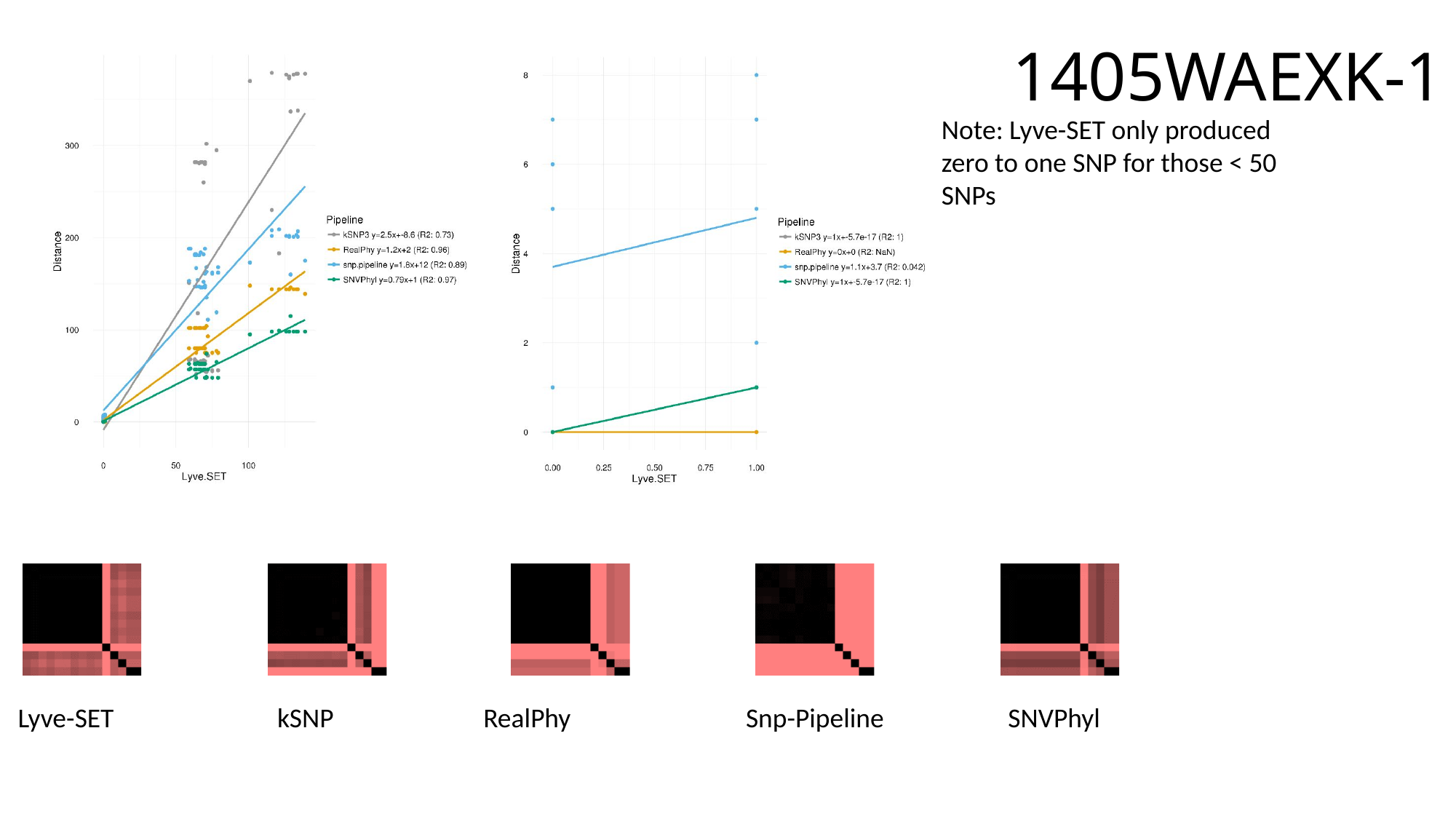

# 1405WAEXK-1
Note: Lyve-SET only produced zero to one SNP for those < 50 SNPs
Lyve-SET
kSNP
RealPhy
Snp-Pipeline
SNVPhyl

## Slide 15
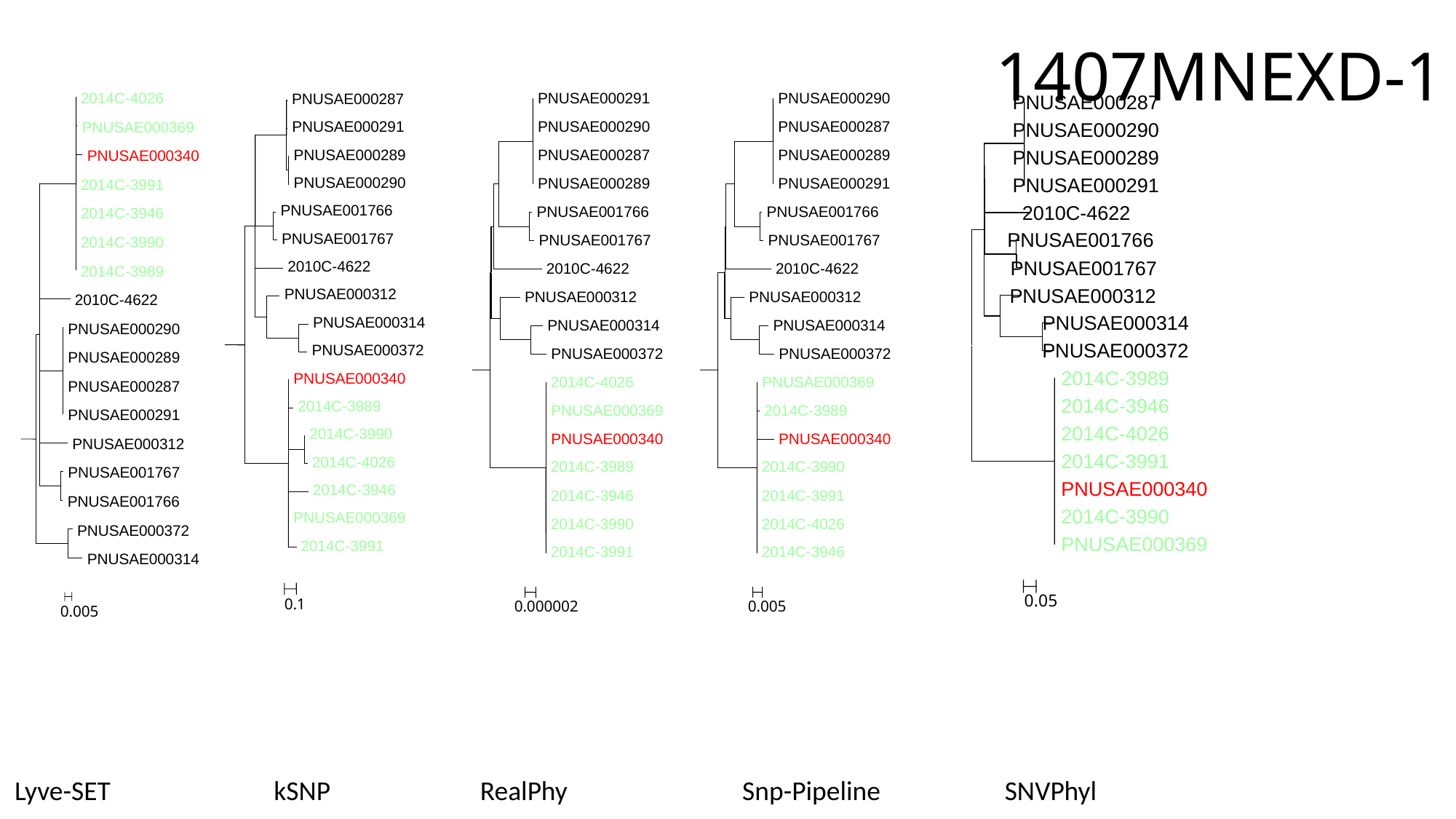

# 1407MNEXD-1
 2014C-4026
 PNUSAE000369
 PNUSAE000340
 2014C-3991
 2014C-3946
 2014C-3990
 2014C-3989
 2010C-4622
 PNUSAE000290
 PNUSAE000289
 PNUSAE000287
 PNUSAE000291
 PNUSAE000312
 PNUSAE001767
 PNUSAE001766
 PNUSAE000372
 PNUSAE000314
0.005
 PNUSAE000287
 PNUSAE000291
 PNUSAE000289
 PNUSAE000290
 PNUSAE001766
 PNUSAE001767
 2010C-4622
 PNUSAE000312
 PNUSAE000314
 PNUSAE000372
 PNUSAE000340
 2014C-3989
 2014C-3990
 2014C-4026
 2014C-3946
 PNUSAE000369
 2014C-3991
0.1
 PNUSAE000291
 PNUSAE000290
 PNUSAE000287
 PNUSAE000289
 PNUSAE001766
 PNUSAE001767
 2010C-4622
 PNUSAE000312
 PNUSAE000314
 PNUSAE000372
 2014C-4026
 PNUSAE000369
 PNUSAE000340
 2014C-3989
 2014C-3946
 2014C-3990
 2014C-3991
0.000002
 PNUSAE000290
 PNUSAE000287
 PNUSAE000289
 PNUSAE000291
 PNUSAE001766
 PNUSAE001767
 2010C-4622
 PNUSAE000312
 PNUSAE000314
 PNUSAE000372
 PNUSAE000369
 2014C-3989
 PNUSAE000340
 2014C-3990
 2014C-3991
 2014C-4026
 2014C-3946
0.005
 PNUSAE000287
 PNUSAE000290
 PNUSAE000289
 PNUSAE000291
 2010C-4622
 PNUSAE001766
 PNUSAE001767
 PNUSAE000312
 PNUSAE000314
 PNUSAE000372
 2014C-3989
 2014C-3946
 2014C-4026
 2014C-3991
 PNUSAE000340
 2014C-3990
 PNUSAE000369
0.05
Lyve-SET
kSNP
RealPhy
Snp-Pipeline
SNVPhyl

## Slide 16
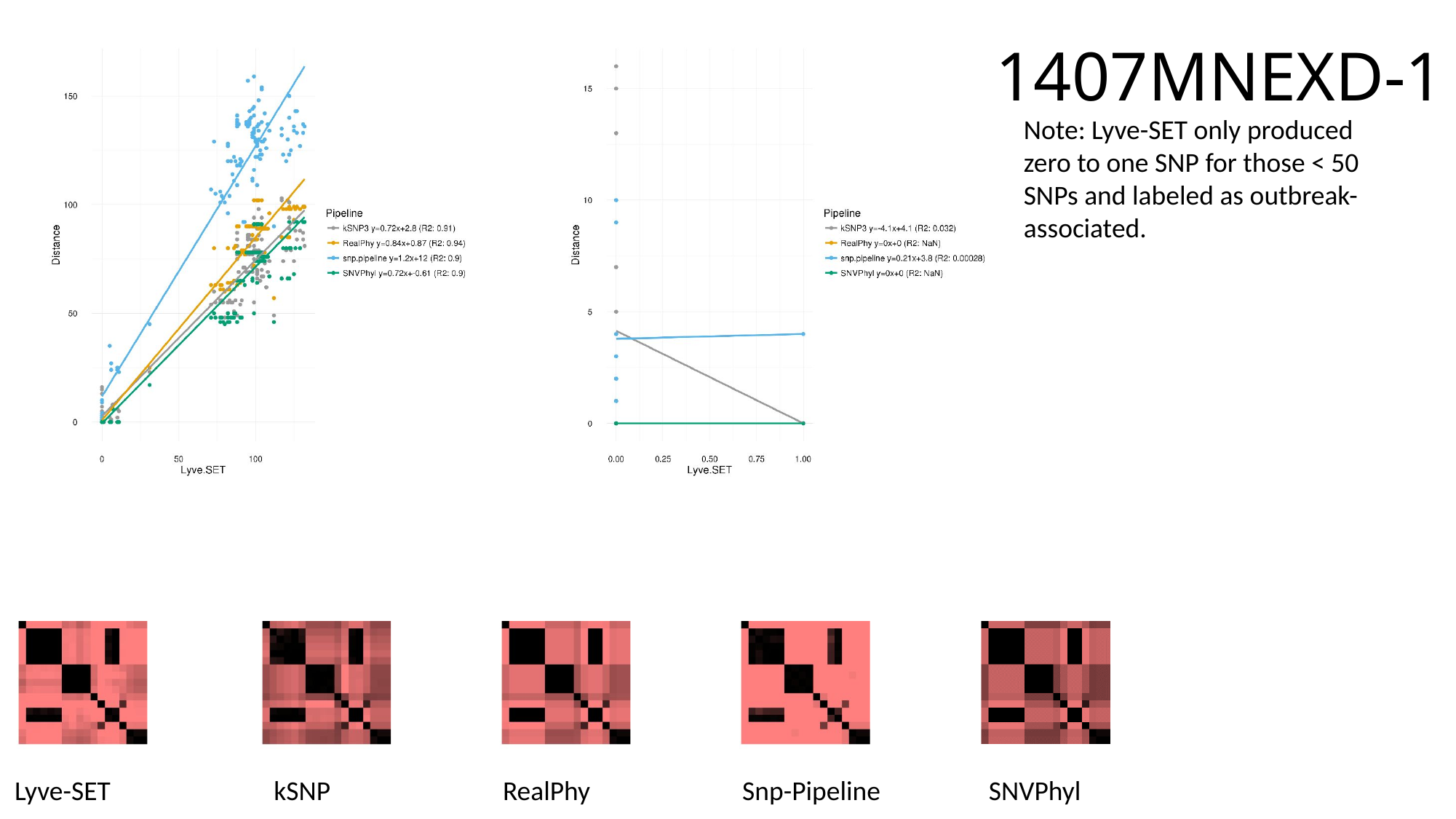

# 1407MNEXD-1
Note: Lyve-SET only produced zero to one SNP for those < 50 SNPs and labeled as outbreak-associated.
Lyve-SET
kSNP
RealPhy
Snp-Pipeline
SNVPhyl

## Slide 17
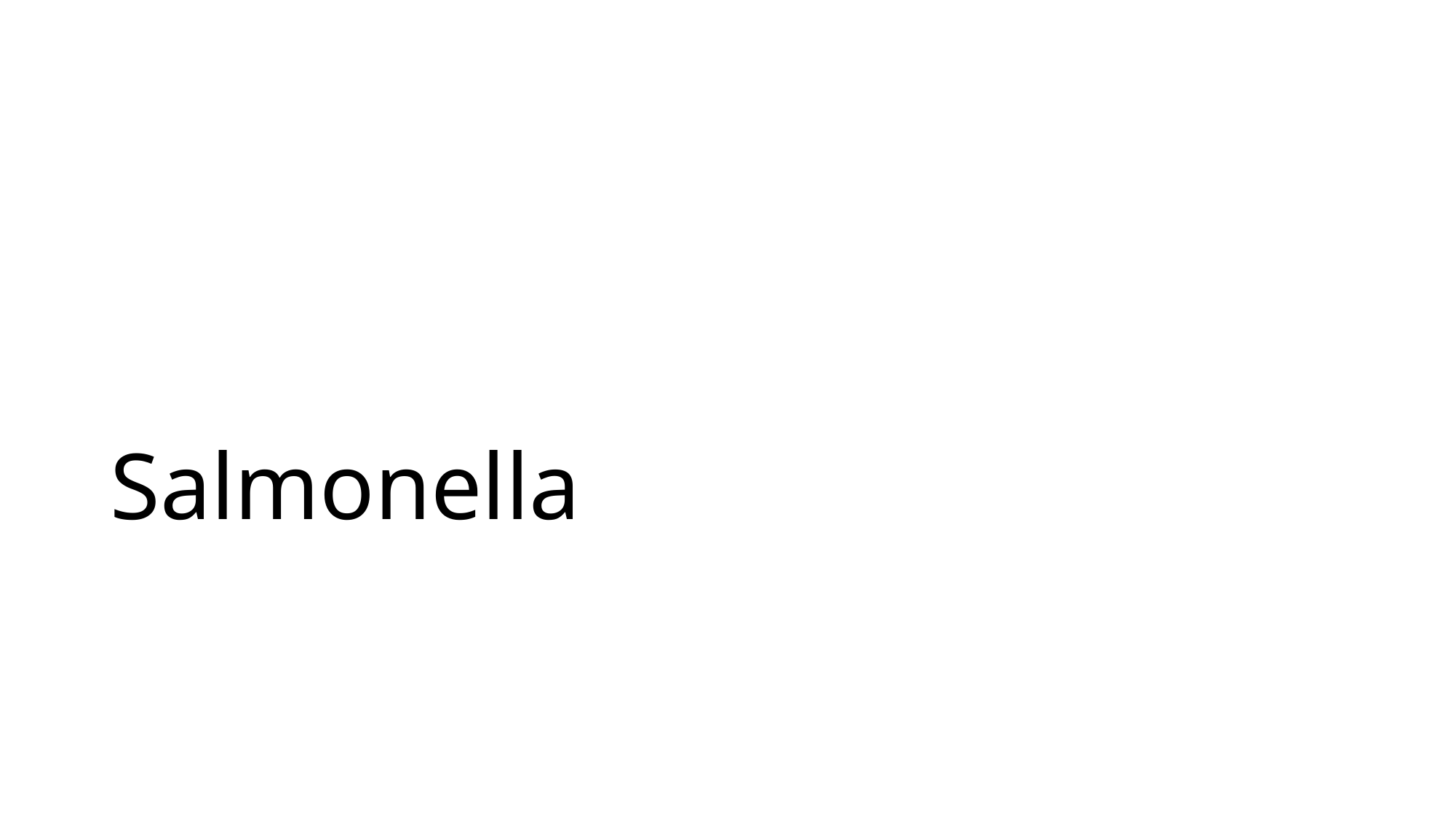

# Salmonella

## Slide 18
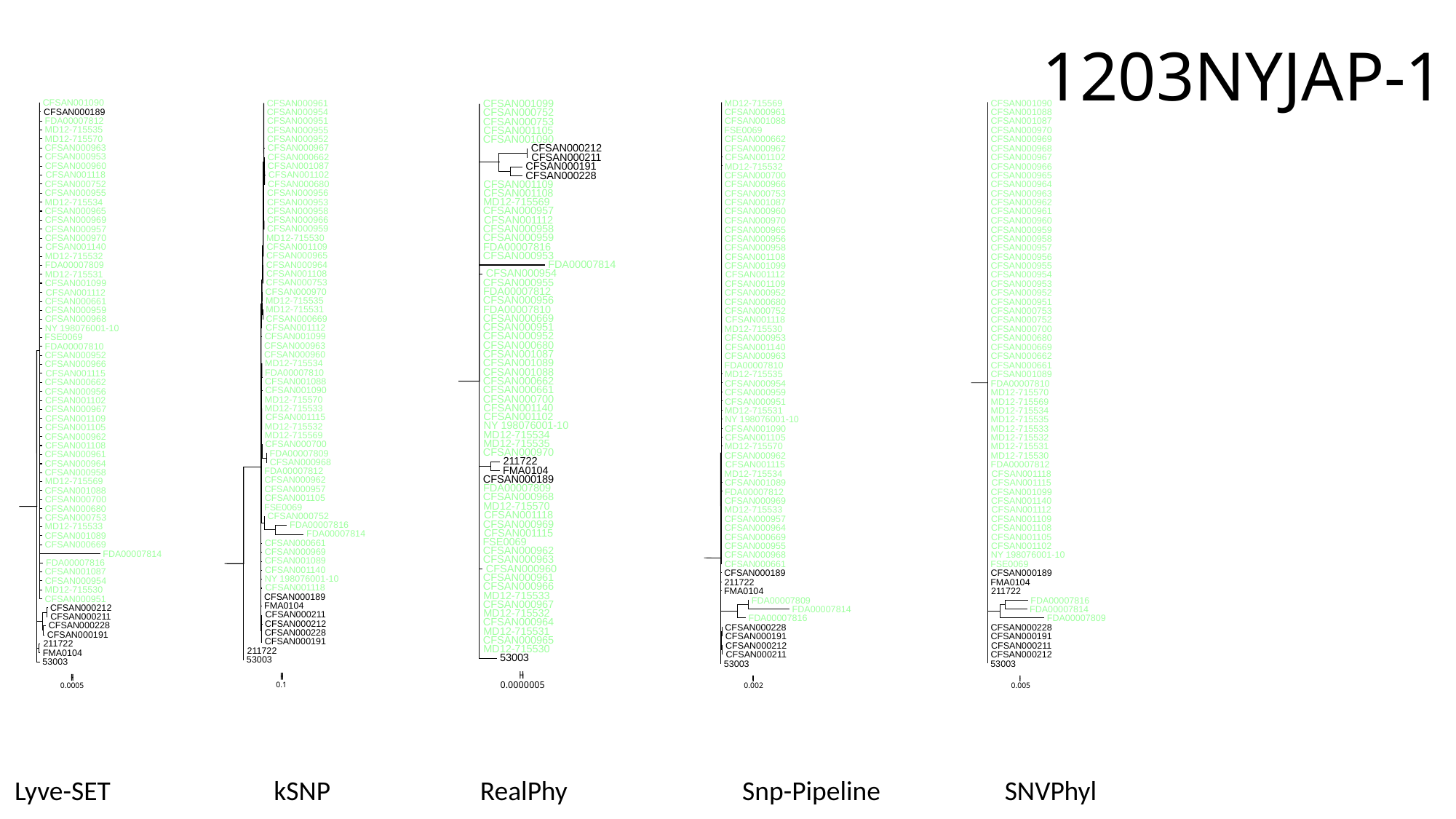

# 1203NYJAP-1
 CFSAN001090
 CFSAN000189
 FDA00007812
 MD12-715535
 MD12-715570
 CFSAN000963
 CFSAN000953
 CFSAN000960
 CFSAN001118
 CFSAN000752
 CFSAN000955
 MD12-715534
 CFSAN000965
 CFSAN000969
 CFSAN000957
 CFSAN000970
 CFSAN001140
 MD12-715532
 FDA00007809
 MD12-715531
 CFSAN001099
 CFSAN001112
 CFSAN000661
 CFSAN000959
 CFSAN000968
 NY 198076001-10
 FSE0069
 FDA00007810
 CFSAN000952
 CFSAN000966
 CFSAN001115
 CFSAN000662
 CFSAN000956
 CFSAN001102
 CFSAN000967
 CFSAN001109
 CFSAN001105
 CFSAN000962
 CFSAN001108
 CFSAN000961
 CFSAN000964
 CFSAN000958
 MD12-715569
 CFSAN001088
 CFSAN000700
 CFSAN000680
 CFSAN000753
 MD12-715533
 CFSAN001089
 CFSAN000669
 FDA00007814
 FDA00007816
 CFSAN001087
 CFSAN000954
 MD12-715530
 CFSAN000951
 CFSAN000212
 CFSAN000211
 CFSAN000228
 CFSAN000191
 211722
 FMA0104
 53003
0.0005
 CFSAN000961
 CFSAN000954
 CFSAN000951
 CFSAN000955
 CFSAN000952
 CFSAN000967
 CFSAN000662
 CFSAN001087
 CFSAN001102
 CFSAN000680
 CFSAN000956
 CFSAN000953
 CFSAN000958
 CFSAN000966
 CFSAN000959
 MD12-715530
 CFSAN001109
 CFSAN000965
 CFSAN000964
 CFSAN001108
 CFSAN000753
 CFSAN000970
 MD12-715535
 MD12-715531
 CFSAN000669
 CFSAN001112
 CFSAN001099
 CFSAN000963
 CFSAN000960
 MD12-715534
 FDA00007810
 CFSAN001088
 CFSAN001090
 MD12-715570
 MD12-715533
 CFSAN001115
 MD12-715532
 MD12-715569
 CFSAN000700
 FDA00007809
 CFSAN000968
 FDA00007812
 CFSAN000962
 CFSAN000957
 CFSAN001105
 FSE0069
 CFSAN000752
 FDA00007816
 FDA00007814
 CFSAN000661
 CFSAN000969
 CFSAN001089
 CFSAN001140
 NY 198076001-10
 CFSAN001118
 CFSAN000189
 FMA0104
 CFSAN000211
 CFSAN000212
 CFSAN000228
 CFSAN000191
 211722
 53003
0.1
 CFSAN001099
 CFSAN000752
 CFSAN000753
 CFSAN001105
 CFSAN001090
 CFSAN000212
 CFSAN000211
 CFSAN000191
 CFSAN000228
 CFSAN001109
 CFSAN001108
 MD12-715569
 CFSAN000957
 CFSAN001112
 CFSAN000958
 CFSAN000959
 FDA00007816
 CFSAN000953
 FDA00007814
 CFSAN000954
 CFSAN000955
 FDA00007812
 CFSAN000956
 FDA00007810
 CFSAN000669
 CFSAN000951
 CFSAN000952
 CFSAN000680
 CFSAN001087
 CFSAN001089
 CFSAN001088
 CFSAN000662
 CFSAN000661
 CFSAN000700
 CFSAN001140
 CFSAN001102
 NY 198076001-10
 MD12-715534
 MD12-715535
 CFSAN000970
 211722
 FMA0104
 CFSAN000189
 FDA00007809
 CFSAN000968
 MD12-715570
 CFSAN001118
 CFSAN000969
 CFSAN001115
 FSE0069
 CFSAN000962
 CFSAN000963
 CFSAN000960
 CFSAN000961
 CFSAN000966
 MD12-715533
 CFSAN000967
 MD12-715532
 CFSAN000964
 MD12-715531
 CFSAN000965
 MD12-715530
 53003
0.0000005
 MD12-715569
 CFSAN000961
 CFSAN001088
 FSE0069
 CFSAN000662
 CFSAN000967
 CFSAN001102
 MD12-715532
 CFSAN000700
 CFSAN000966
 CFSAN000753
 CFSAN001087
 CFSAN000960
 CFSAN000970
 CFSAN000965
 CFSAN000956
 CFSAN000958
 CFSAN001108
 CFSAN001099
 CFSAN001112
 CFSAN001109
 CFSAN000952
 CFSAN000680
 CFSAN000752
 CFSAN001118
 MD12-715530
 CFSAN000953
 CFSAN001140
 CFSAN000963
 FDA00007810
 MD12-715535
 CFSAN000954
 CFSAN000959
 CFSAN000951
 MD12-715531
 NY 198076001-10
 CFSAN001090
 CFSAN001105
 MD12-715570
 CFSAN000962
 CFSAN001115
 MD12-715534
 CFSAN001089
 FDA00007812
 CFSAN000969
 MD12-715533
 CFSAN000957
 CFSAN000964
 CFSAN000669
 CFSAN000955
 CFSAN000968
 CFSAN000661
 CFSAN000189
 211722
 FMA0104
 FDA00007809
 FDA00007814
 FDA00007816
 CFSAN000228
 CFSAN000191
 CFSAN000212
 CFSAN000211
 53003
0.002
 CFSAN001090
 CFSAN001088
 CFSAN001087
 CFSAN000970
 CFSAN000969
 CFSAN000968
 CFSAN000967
 CFSAN000966
 CFSAN000965
 CFSAN000964
 CFSAN000963
 CFSAN000962
 CFSAN000961
 CFSAN000960
 CFSAN000959
 CFSAN000958
 CFSAN000957
 CFSAN000956
 CFSAN000955
 CFSAN000954
 CFSAN000953
 CFSAN000952
 CFSAN000951
 CFSAN000753
 CFSAN000752
 CFSAN000700
 CFSAN000680
 CFSAN000669
 CFSAN000662
 CFSAN000661
 CFSAN001089
 FDA00007810
 MD12-715570
 MD12-715569
 MD12-715534
 MD12-715535
 MD12-715533
 MD12-715532
 MD12-715531
 MD12-715530
 FDA00007812
 CFSAN001118
 CFSAN001115
 CFSAN001099
 CFSAN001140
 CFSAN001112
 CFSAN001109
 CFSAN001108
 CFSAN001105
 CFSAN001102
 NY 198076001-10
 FSE0069
 CFSAN000189
 FMA0104
 211722
 FDA00007816
 FDA00007814
 FDA00007809
 CFSAN000228
 CFSAN000191
 CFSAN000211
 CFSAN000212
 53003
0.005
Lyve-SET
kSNP
RealPhy
Snp-Pipeline
SNVPhyl

## Slide 19
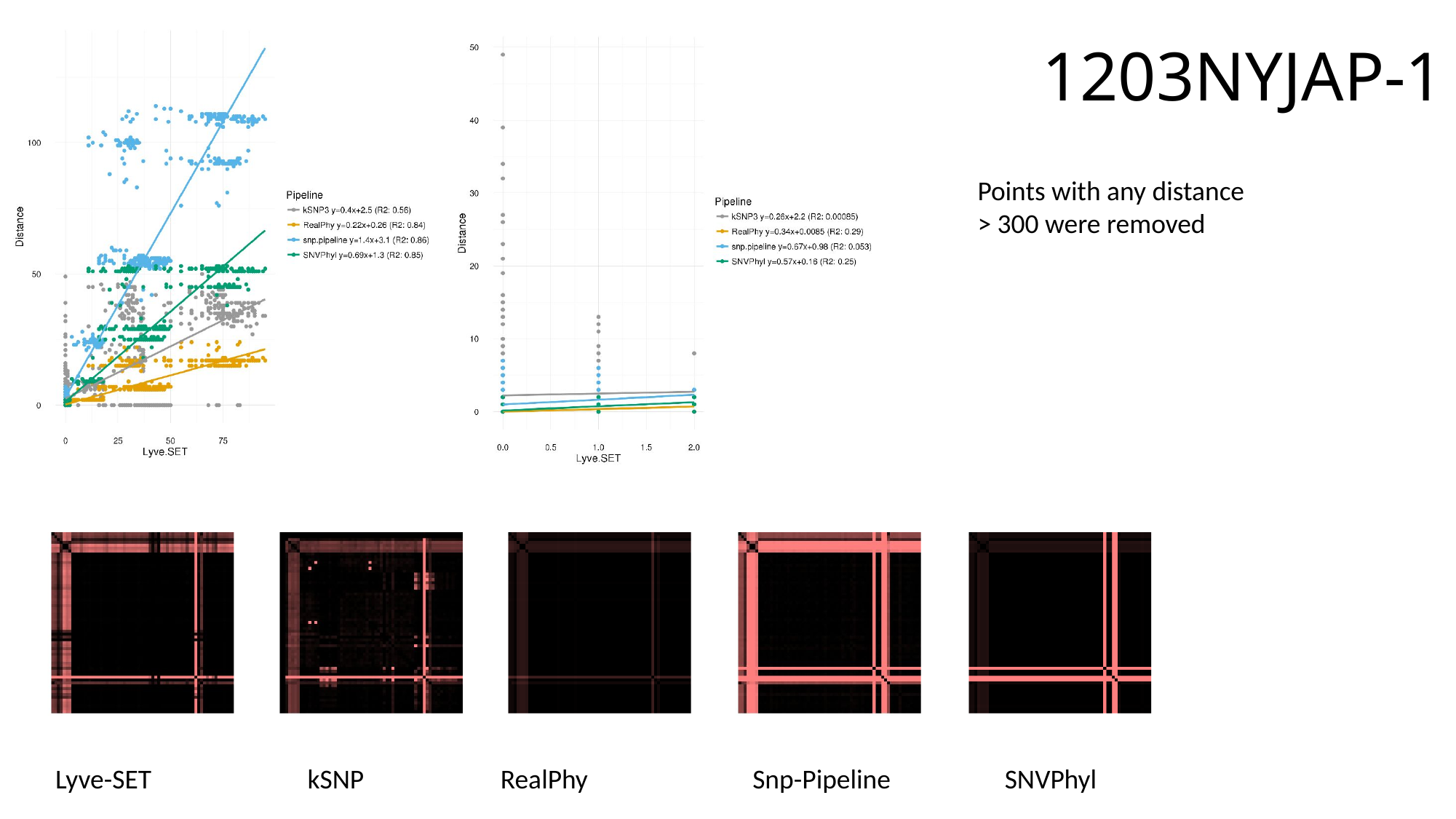

# 1203NYJAP-1
Points with any distance > 300 were removed
Lyve-SET
kSNP
RealPhy
Snp-Pipeline
SNVPhyl

## Slide 20
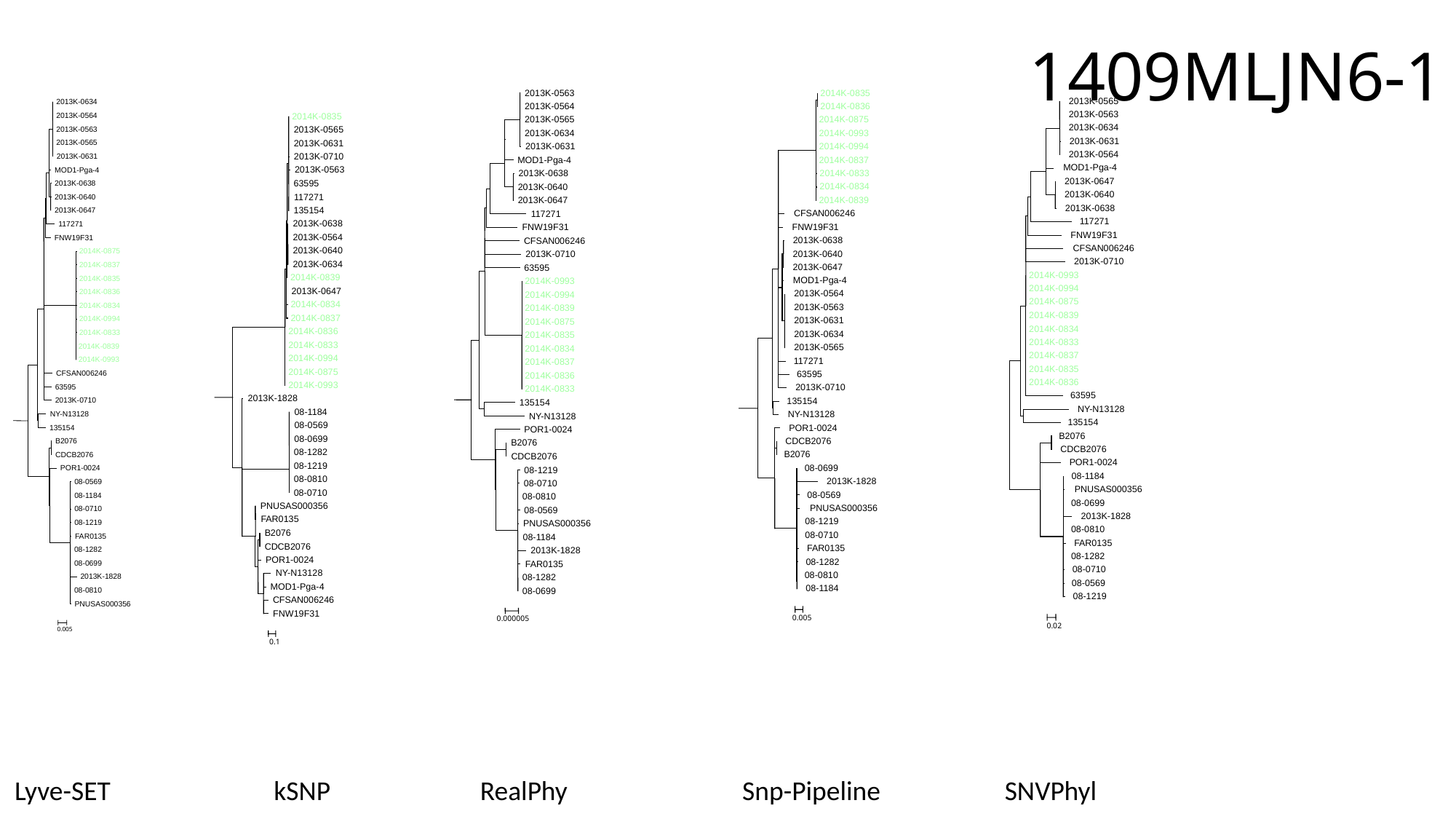

# 1409MLJN6-1
 2013K-0563
 2013K-0564
 2013K-0565
 2013K-0634
 2013K-0631
 MOD1-Pga-4
 2013K-0638
 2013K-0640
 2013K-0647
 117271
 FNW19F31
 CFSAN006246
 2013K-0710
 63595
 2014K-0993
 2014K-0994
 2014K-0839
 2014K-0875
 2014K-0835
 2014K-0834
 2014K-0837
 2014K-0836
 2014K-0833
 135154
 NY-N13128
 POR1-0024
 B2076
 CDCB2076
 08-1219
 08-0710
 08-0810
 08-0569
 PNUSAS000356
 08-1184
 2013K-1828
 FAR0135
 08-1282
 08-0699
0.000005
 2014K-0835
 2014K-0836
 2014K-0875
 2014K-0993
 2014K-0994
 2014K-0837
 2014K-0833
 2014K-0834
 2014K-0839
 CFSAN006246
 FNW19F31
 2013K-0638
 2013K-0640
 2013K-0647
 MOD1-Pga-4
 2013K-0564
 2013K-0563
 2013K-0631
 2013K-0634
 2013K-0565
 117271
 63595
 2013K-0710
 135154
 NY-N13128
 POR1-0024
 CDCB2076
 B2076
 08-0699
 2013K-1828
 08-0569
 PNUSAS000356
 08-1219
 08-0710
 FAR0135
 08-1282
 08-0810
 08-1184
0.005
 2013K-0634
 2013K-0564
 2013K-0563
 2013K-0565
 2013K-0631
 MOD1-Pga-4
 2013K-0638
 2013K-0640
 2013K-0647
 117271
 FNW19F31
 2014K-0875
 2014K-0837
 2014K-0835
 2014K-0836
 2014K-0834
 2014K-0994
 2014K-0833
 2014K-0839
 2014K-0993
 CFSAN006246
 63595
 2013K-0710
 NY-N13128
 135154
 B2076
 CDCB2076
 POR1-0024
 08-0569
 08-1184
 08-0710
 08-1219
 FAR0135
 08-1282
 08-0699
 2013K-1828
 08-0810
 PNUSAS000356
0.005
 2013K-0565
 2014K-0835
 2013K-0565
 2013K-0631
 2013K-0710
 2013K-0563
 63595
 117271
 135154
 2013K-0638
 2013K-0564
 2013K-0640
 2013K-0634
 2014K-0839
 2013K-0647
 2014K-0834
 2014K-0837
 2014K-0836
 2014K-0833
 2014K-0994
 2014K-0875
 2014K-0993
 2013K-1828
 08-1184
 08-0569
 08-0699
 08-1282
 08-1219
 08-0810
 08-0710
 PNUSAS000356
 FAR0135
 B2076
 CDCB2076
 POR1-0024
 NY-N13128
 MOD1-Pga-4
 CFSAN006246
 FNW19F31
0.1
 2013K-0563
 2013K-0634
 2013K-0631
 2013K-0564
 MOD1-Pga-4
 2013K-0647
 2013K-0640
 2013K-0638
 117271
 FNW19F31
 CFSAN006246
 2013K-0710
 2014K-0993
 2014K-0994
 2014K-0875
 2014K-0839
 2014K-0834
 2014K-0833
 2014K-0837
 2014K-0835
 2014K-0836
 63595
 NY-N13128
 135154
 B2076
 CDCB2076
 POR1-0024
 08-1184
 PNUSAS000356
 08-0699
 2013K-1828
 08-0810
 FAR0135
 08-1282
 08-0710
 08-0569
 08-1219
0.02
Lyve-SET
kSNP
RealPhy
Snp-Pipeline
SNVPhyl

## Slide 21
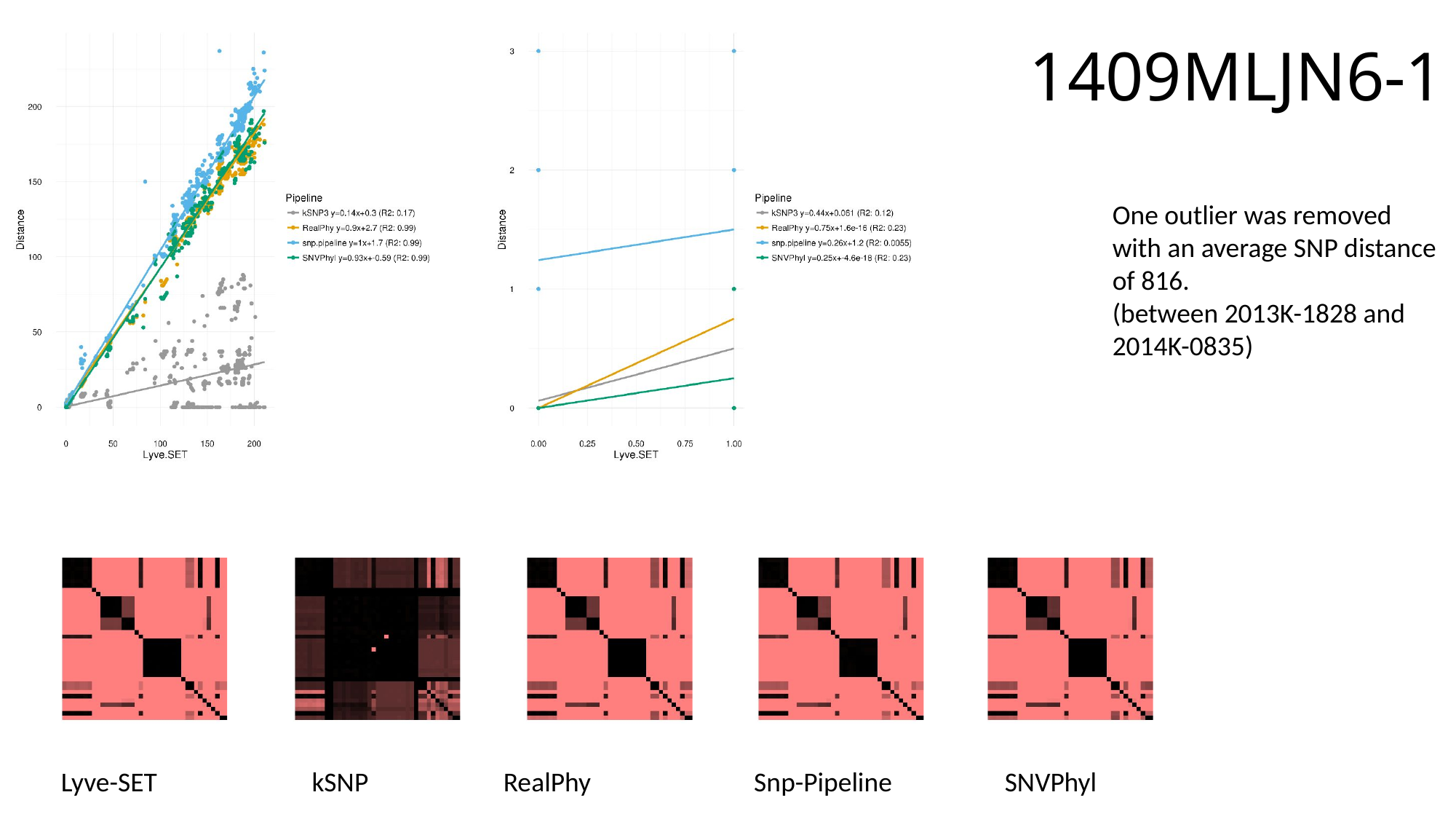

# 1409MLJN6-1
One outlier was removed with an average SNP distance of 816.
(between 2013K-1828 and 2014K-0835)
Lyve-SET
kSNP
RealPhy
Snp-Pipeline
SNVPhyl

## Slide 22
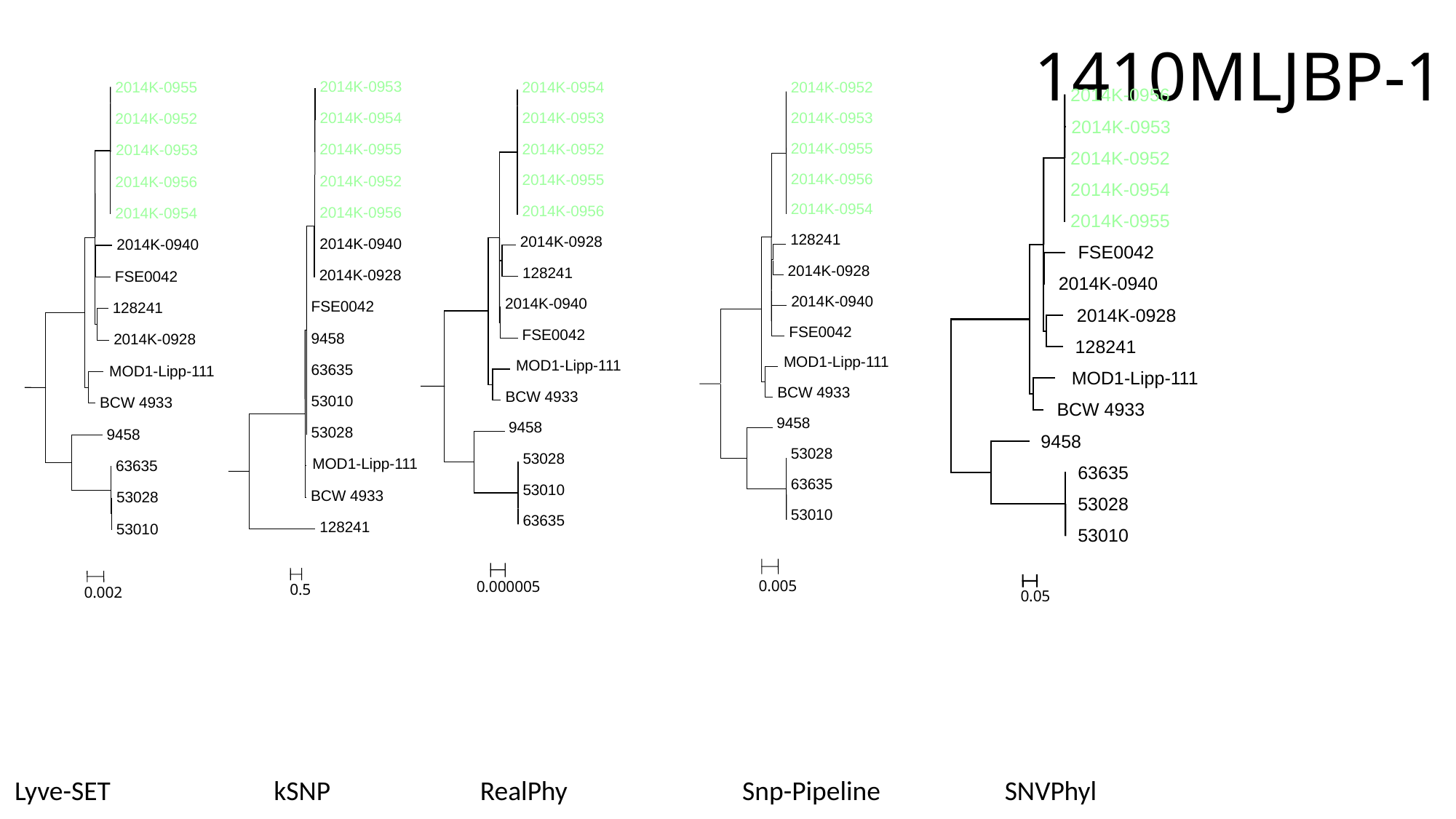

# 1410MLJBP-1
 2014K-0955
 2014K-0952
 2014K-0953
 2014K-0956
 2014K-0954
 2014K-0940
 FSE0042
 128241
 2014K-0928
 MOD1-Lipp-111
 BCW 4933
 9458
 63635
 53028
 53010
0.002
 2014K-0953
 2014K-0954
 2014K-0955
 2014K-0952
 2014K-0956
 2014K-0940
 2014K-0928
 FSE0042
 9458
 63635
 53010
 53028
 MOD1-Lipp-111
 BCW 4933
 128241
0.5
 2014K-0954
 2014K-0953
 2014K-0952
 2014K-0955
 2014K-0956
 2014K-0928
 128241
 2014K-0940
 FSE0042
 MOD1-Lipp-111
 BCW 4933
 9458
 53028
 53010
 63635
0.000005
 2014K-0952
 2014K-0953
 2014K-0955
 2014K-0956
 2014K-0954
 128241
 2014K-0928
 2014K-0940
 FSE0042
 MOD1-Lipp-111
 BCW 4933
 9458
 53028
 63635
 53010
0.005
 2014K-0956
 2014K-0953
 2014K-0952
 2014K-0954
 2014K-0955
 FSE0042
 2014K-0940
 2014K-0928
 128241
 MOD1-Lipp-111
 BCW 4933
 9458
 63635
 53028
 53010
0.05
Lyve-SET
kSNP
RealPhy
Snp-Pipeline
SNVPhyl

## Slide 23
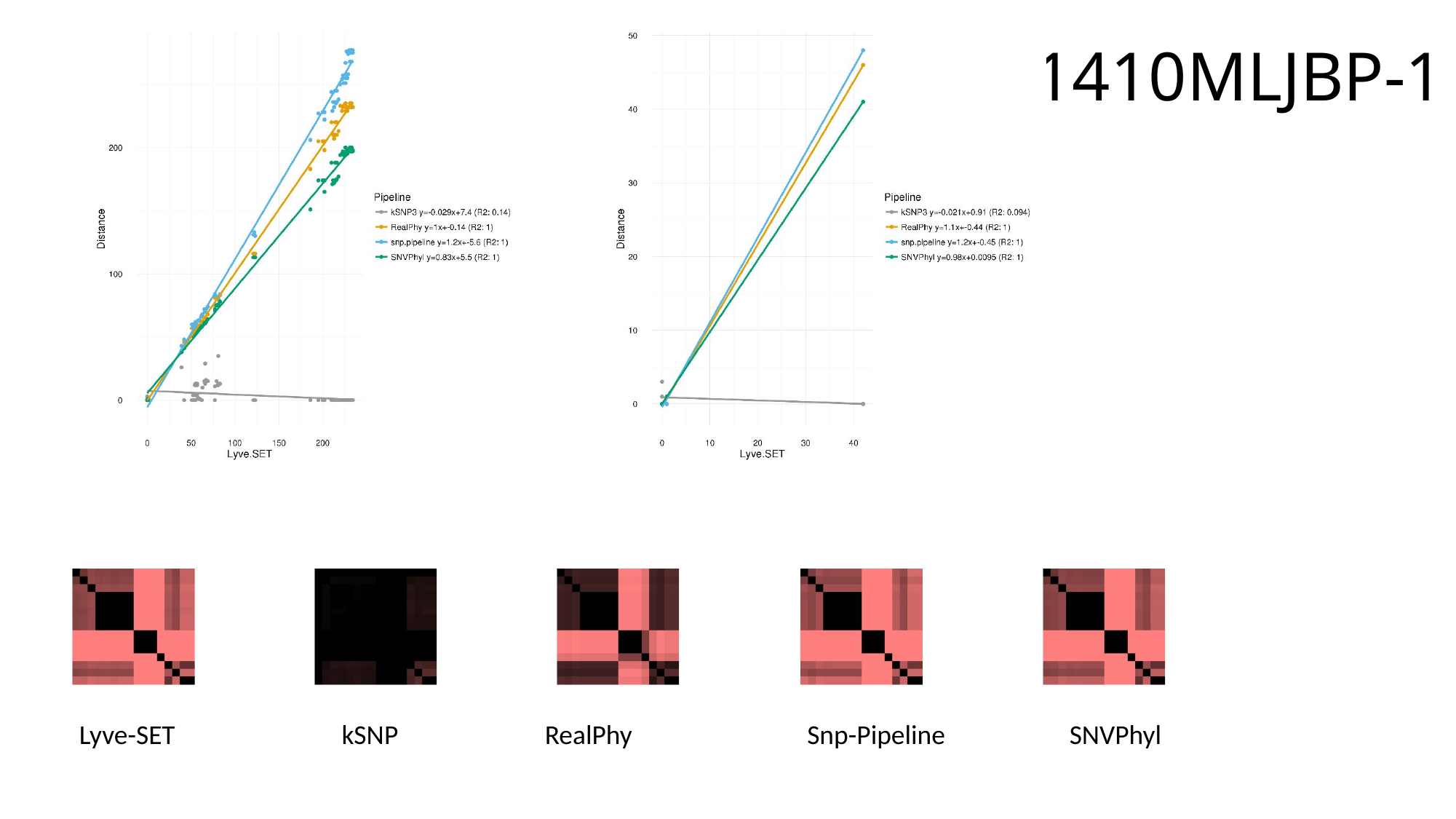

# 1410MLJBP-1
Lyve-SET
kSNP
RealPhy
Snp-Pipeline
SNVPhyl

## Slide 24
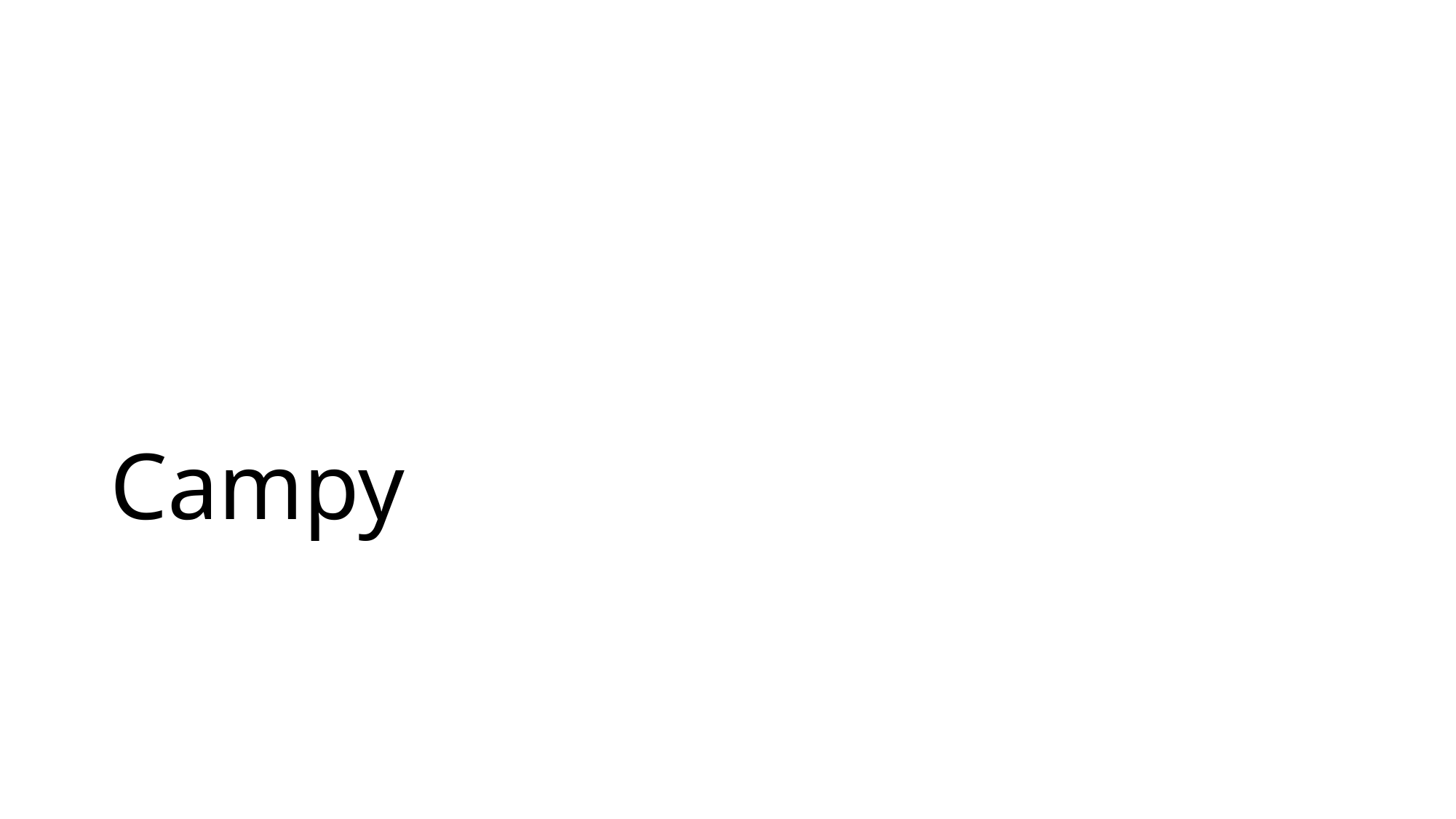

# Campy

## Slide 25
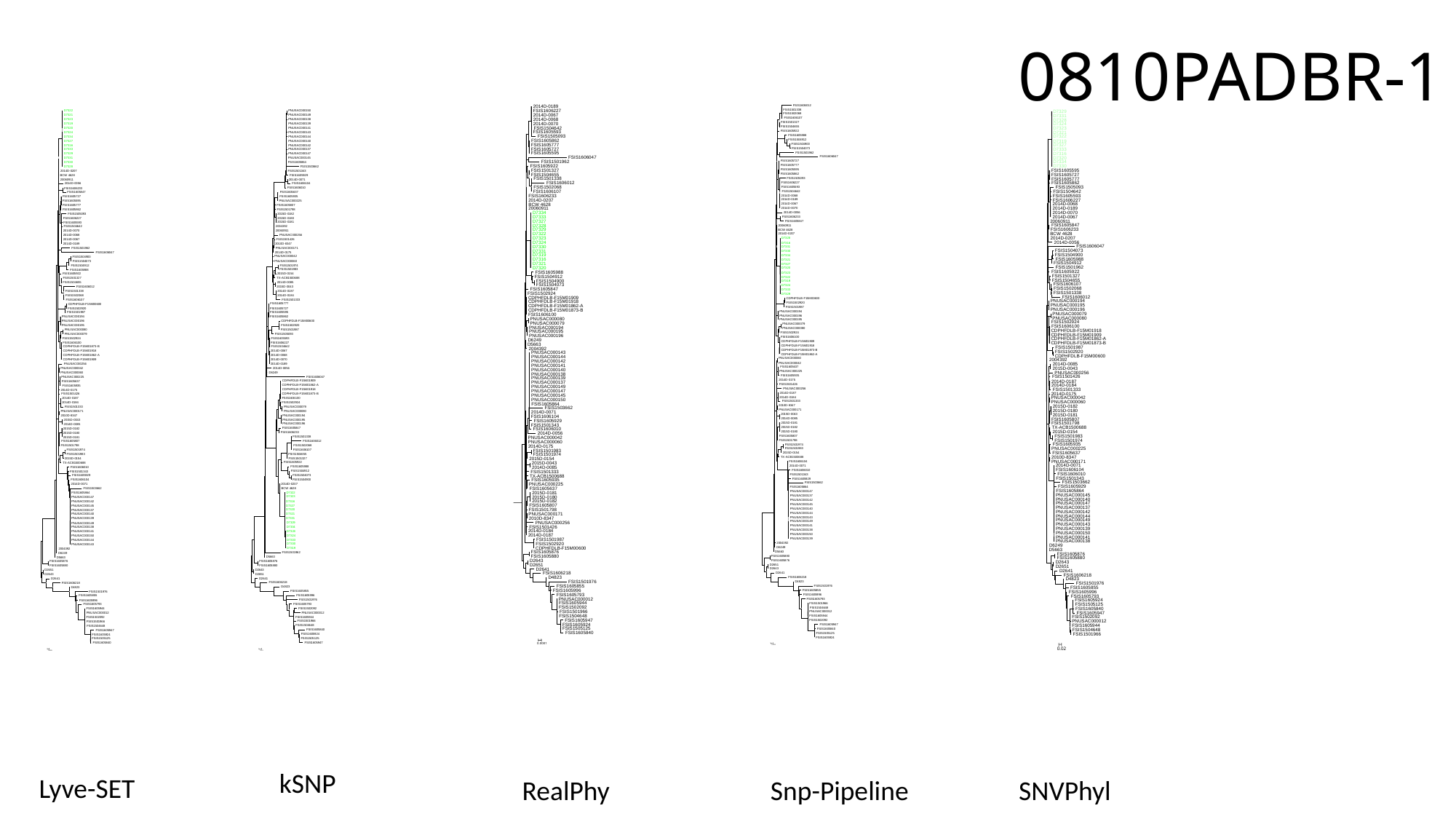

# 0810PADBR-1
 2014D-0189
 FSIS1606227
 2014D-0067
 2014D-0068
 2014D-0070
 FSIS1504642
 FSIS1605593
 FSIS1505093
 FSIS1605862
 FSIS1605777
 FSIS1605727
 FSIS1605595
 FSIS1606047
 FSIS1501962
 FSIS1605922
 FSIS1501327
 FSIS1504655
 FSIS1501338
 FSIS1606012
 FSIS1502068
 FSIS1606107
 FSIS1606233
 2014D-0207
 BCW 4628
 20060911
 D7334
 D7333
 D7327
 D7328
 D7329
 D7322
 D7323
 D7324
 D7330
 D7331
 D7319
 D7316
 D7321
 D7320
 FSIS1605988
 FSIS1504912
 FSIS1504900
 FSIS1504073
 FSIS1605847
 FSIS1502924
 CDPHFDLB-F15M01909
 CDPHFDLB-F15M01918
 CDPHFDLB-F15M01862-A
 CDPHFDLB-F15M01873-B
 FSIS1606100
 PNUSAC000080
 PNUSAC000079
 PNUSAC000194
 PNUSAC000195
 PNUSAC000196
 D6249
 D5663
 2004392
 PNUSAC000143
 PNUSAC000144
 PNUSAC000142
 PNUSAC000141
 PNUSAC000140
 PNUSAC000138
 PNUSAC000139
 PNUSAC000137
 PNUSAC000149
 PNUSAC000147
 PNUSAC000145
 PNUSAC000150
 FSIS1605864
 FSIS1503662
 2014D-0071
 FSIS1606104
 FSIS1605929
 FSIS1501343
 FSIS1606010
 2014D-0056
 PNUSAC000042
 PNUSAC000060
 2014D-0175
 FSIS1501983
 FSIS1501974
 2015D-0154
 2015D-0043
 2014D-0085
 FSIS1501333
 TX-ACB1500688
 FSIS1605935
 PNUSAC000225
 FSIS1605637
 2015D-0181
 2015D-0180
 2015D-0182
 FSIS1605807
 FSIS1501798
 PNUSAC000171
 2010D-8347
 PNUSAC000256
 FSIS1501426
 2014D-0184
 2014D-0187
 FSIS1501987
 FSIS1502920
 CDPHFDLB-F15M00600
 FSIS1605876
 FSIS1605880
 D2643
 D2651
 D2641
 FSIS1606218
 D4823
 FSIS1501976
 FSIS1605855
 FSIS1605996
 FSIS1605793
 PNUSAC000012
 FSIS1605944
 FSIS1502092
 FSIS1501966
 FSIS1504648
 FSIS1605947
 FSIS1605924
 FSIS1505125
 FSIS1605840
0.0001
 FSIS1606012
 FSIS1501338
 FSIS1502068
 FSIS1606107
 FSIS1501327
 FSIS1504655
 FSIS1605922
 FSIS1605988
 FSIS1504912
 FSIS1504900
 FSIS1504073
 FSIS1501962
 FSIS1606047
 FSIS1605727
 FSIS1605777
 FSIS1605595
 FSIS1605862
 FSIS1505093
 FSIS1606227
 FSIS1605593
 FSIS1504642
 2014D-0068
 2014D-0189
 2014D-0067
 2014D-0070
 2014D-0056
 FSIS1606233
 FSIS1605847
 20060911
 BCW 4628
 2014D-0207
 D7328
 D7316
 D7331
 D7330
 D7334
 D7321
 D7327
 D7320
 D7323
 D7322
 D7319
 D7324
 D7333
 D7329
 CDPHFDLB-F15M00600
 FSIS1502920
 FSIS1501987
 PNUSAC000194
 PNUSAC000196
 PNUSAC000195
 PNUSAC000079
 PNUSAC000080
 FSIS1502924
 FSIS1606100
 CDPHFDLB-F15M01909
 CDPHFDLB-F15M01918
 CDPHFDLB-F15M01873-B
 CDPHFDLB-F15M01862-A
 PNUSAC000060
 PNUSAC000042
 FSIS1605637
 PNUSAC000225
 FSIS1605935
 2014D-0175
 FSIS1501426
 PNUSAC000256
 2014D-0187
 2014D-0184
 FSIS1501333
 2010D-8347
 PNUSAC000171
 2015D-0043
 2014D-0085
 2015D-0181
 2015D-0182
 2015D-0180
 FSIS1605807
 FSIS1501798
 FSIS1501974
 FSIS1501983
 2015D-0154
 TX-ACB1500688
 FSIS1606104
 2014D-0071
 FSIS1606010
 FSIS1501343
4
 FSIS1605929
 FSIS1503662
 FSIS1605864
 PNUSAC000147
 PNUSAC000137
 PNUSAC000142
 PNUSAC000145
 PNUSAC000140
 PNUSAC000144
 PNUSAC000143
 PNUSAC000149
 PNUSAC000141
 PNUSAC000138
 PNUSAC000150
 PNUSAC000139
 2004392
 D6249
 D5663
 FSIS1605880
 FSIS1605876
 D2651
 D2643
 D2641
 FSIS1606218
 D4823
 FSIS1501976
 FSIS1605855
 FSIS1605996
 FSIS1605793
 FSIS1501966
 FSIS1504648
 PNUSAC000012
 FSIS1605944
 FSIS1502092
 FSIS1605947
 FSIS1605840
 FSIS1505125
 FSIS1605924
0.005
 D7322
 D7321
 D7323
 D7319
 D7320
 D7324
 D7334
 D7327
 D7316
 D7333
 D7329
 D7331
 D7330
 D7328
 2014D-0207
 BCW 4628
 20060911
 2014D-0056
 FSIS1606233
 FSIS1605847
 FSIS1605727
 FSIS1605595
 FSIS1605777
 FSIS1605862
 FSIS1505093
 FSIS1606227
 FSIS1605593
 FSIS1504642
 2014D-0070
 2014D-0068
 2014D-0067
 2014D-0189
 FSIS1501962
 FSIS1606047
 FSIS1504900
 FSIS1504073
 FSIS1504912
 FSIS1605988
 FSIS1605922
 FSIS1501327
 FSIS1504655
 FSIS1606012
 FSIS1501338
 FSIS1502068
 FSIS1606107
 CDPHFDLB-F15M00600
 FSIS1502920
 FSIS1501987
 PNUSAC000194
 PNUSAC000196
 PNUSAC000195
 PNUSAC000080
 PNUSAC000079
 FSIS1502924
 FSIS1606100
 CDPHFDLB-F15M01873-B
 CDPHFDLB-F15M01918
 CDPHFDLB-F15M01862-A
 CDPHFDLB-F15M01909
 PNUSAC000256
 PNUSAC000042
 PNUSAC000060
 PNUSAC000225
 FSIS1605637
 FSIS1605935
 2014D-0175
 FSIS1501426
 2014D-0187
 2014D-0184
 FSIS1501333
 PNUSAC000171
 2010D-8347
 2015D-0043
 2014D-0085
 2015D-0182
 2015D-0180
 2015D-0181
 FSIS1605807
 FSIS1501798
 FSIS1501974
 FSIS1501983
 2015D-0154
 TX-ACB1500688
 FSIS1606010
 FSIS1501343
 FSIS1605929
 FSIS1606104
 2014D-0071
 FSIS1503662
 FSIS1605864
 PNUSAC000147
 PNUSAC000142
 PNUSAC000145
 PNUSAC000137
 PNUSAC000140
 PNUSAC000139
 PNUSAC000149
 PNUSAC000138
 PNUSAC000141
 PNUSAC000150
 PNUSAC000144
 PNUSAC000143
 2004392
 D6249
 D5663
 FSIS1605876
 FSIS1605880
 D2651
 D2643
 D2641
 FSIS1606218
 D4823
 FSIS1501976
 FSIS1605855
 FSIS1605996
 FSIS1605793
 FSIS1605944
 PNUSAC000012
 FSIS1502092
 FSIS1501966
 FSIS1504648
 FSIS1605947
 FSIS1605924
 FSIS1505125
 FSIS1605840
0.005
 PNUSAC000150
 PNUSAC000149
 PNUSAC000138
 PNUSAC000139
 PNUSAC000141
 PNUSAC000143
 PNUSAC000144
 PNUSAC000140
 PNUSAC000142
 PNUSAC000137
 PNUSAC000147
 PNUSAC000145
 FSIS1605864
 FSIS1503662
 FSIS1501343
 FSIS1605929
 2014D-0071
 FSIS1606104
 FSIS1606010
 FSIS1605637
 FSIS1605935
 PNUSAC000225
 FSIS1605807
 FSIS1501798
 2015D-0182
 2015D-0180
 2015D-0181
 2004392
 20060911
 PNUSAC000256
 FSIS1501426
 2010D-8347
 PNUSAC000171
 2014D-0175
 PNUSAC000042
 PNUSAC000060
 FSIS1501974
 FSIS1501983
 2015D-0154
 TX-ACB1500688
 2014D-0085
 2015D-0043
 2014D-0187
 2014D-0184
 FSIS1501333
 FSIS1605777
 FSIS1605727
 FSIS1605595
 FSIS1605862
 CDPHFDLB-F15M00600
 FSIS1502920
 FSIS1501987
 FSIS1505093
 FSIS1605593
 FSIS1606227
 FSIS1504642
 2014D-0067
 2014D-0068
 2014D-0070
 2014D-0189
 2014D-0056
 D6249
 FSIS1606047
 CDPHFDLB-F15M01909
 CDPHFDLB-F15M01862-A
 CDPHFDLB-F15M01918
 CDPHFDLB-F15M01873-B
 FSIS1606100
 FSIS1502924
 PNUSAC000079
 PNUSAC000080
 PNUSAC000194
 PNUSAC000195
 PNUSAC000196
 FSIS1605847
 FSIS1606233
 FSIS1501338
 FSIS1606012
 FSIS1502068
 FSIS1606107
 FSIS1504655
 FSIS1501327
 FSIS1605922
 FSIS1605988
 FSIS1504912
 FSIS1504073
 FSIS1504900
 2014D-0207
 BCW 4628
 D7322
 D7323
 D7316
 D7327
 D7320
 D7321
 D7331
 D7329
 D7334
 D7328
 D7324
 D7333
 D7330
 D7319
 FSIS1501962
 D5663
 FSIS1605876
 FSIS1605880
 D2643
 D2651
 D2641
 FSIS1606218
 D4823
 FSIS1605855
 FSIS1605996
 FSIS1501976
 FSIS1605793
 FSIS1502092
 PNUSAC000012
 FSIS1605944
 FSIS1501966
 FSIS1504648
 FSIS1605840
 FSIS1605924
 FSIS1505125
 FSIS1605947
0.01
 D7329
 D7331
 D7328
 D7324
 D7323
 D7321
 D7322
 D7319
 D7327
 D7333
 D7316
 D7320
 D7334
 D7330
 FSIS1605595
 FSIS1605727
 FSIS1605777
 FSIS1605862
 FSIS1505093
 FSIS1504642
 FSIS1605593
 FSIS1606227
 2014D-0068
 2014D-0189
 2014D-0070
 2014D-0067
 20060911
 FSIS1605847
 FSIS1606233
 BCW 4628
 2014D-0207
 2014D-0056
 FSIS1606047
 FSIS1504073
 FSIS1504900
 FSIS1605988
 FSIS1504912
 FSIS1501962
 FSIS1605922
 FSIS1501327
 FSIS1504655
 FSIS1606107
 FSIS1502068
 FSIS1501338
 FSIS1606012
 PNUSAC000194
 PNUSAC000195
 PNUSAC000196
 PNUSAC000079
 PNUSAC000080
 FSIS1502924
 FSIS1606100
 CDPHFDLB-F15M01918
 CDPHFDLB-F15M01909
 CDPHFDLB-F15M01862-A
 CDPHFDLB-F15M01873-B
 FSIS1501987
 FSIS1502920
 CDPHFDLB-F15M00600
 2004392
 2014D-0085
 2015D-0043
 PNUSAC000256
 FSIS1501426
 2014D-0187
 2014D-0184
 FSIS1501333
 2014D-0175
 PNUSAC000042
 PNUSAC000060
 2015D-0182
 2015D-0180
 2015D-0181
 FSIS1605807
 FSIS1501798
 TX-ACB1500688
 2015D-0154
 FSIS1501983
 FSIS1501974
 FSIS1605935
 PNUSAC000225
 FSIS1605637
 2010D-8347
 PNUSAC000171
 2014D-0071
 FSIS1606104
 FSIS1606010
 FSIS1501343
 FSIS1503662
 FSIS1605929
 FSIS1605864
 PNUSAC000145
 PNUSAC000140
 PNUSAC000147
 PNUSAC000137
 PNUSAC000142
 PNUSAC000144
 PNUSAC000149
 PNUSAC000143
 PNUSAC000139
 PNUSAC000150
 PNUSAC000141
 PNUSAC000138
 D6249
 D5663
 FSIS1605876
 FSIS1605880
 D2643
 D2651
 D2641
 FSIS1606218
 D4823
 FSIS1501976
 FSIS1605855
 FSIS1605996
 FSIS1605793
 FSIS1605924
 FSIS1505125
 FSIS1605840
 FSIS1605947
 FSIS1502092
 PNUSAC000012
 FSIS1605944
 FSIS1504648
 FSIS1501966
0.02
kSNP
Lyve-SET
RealPhy
Snp-Pipeline
SNVPhyl

## Slide 26
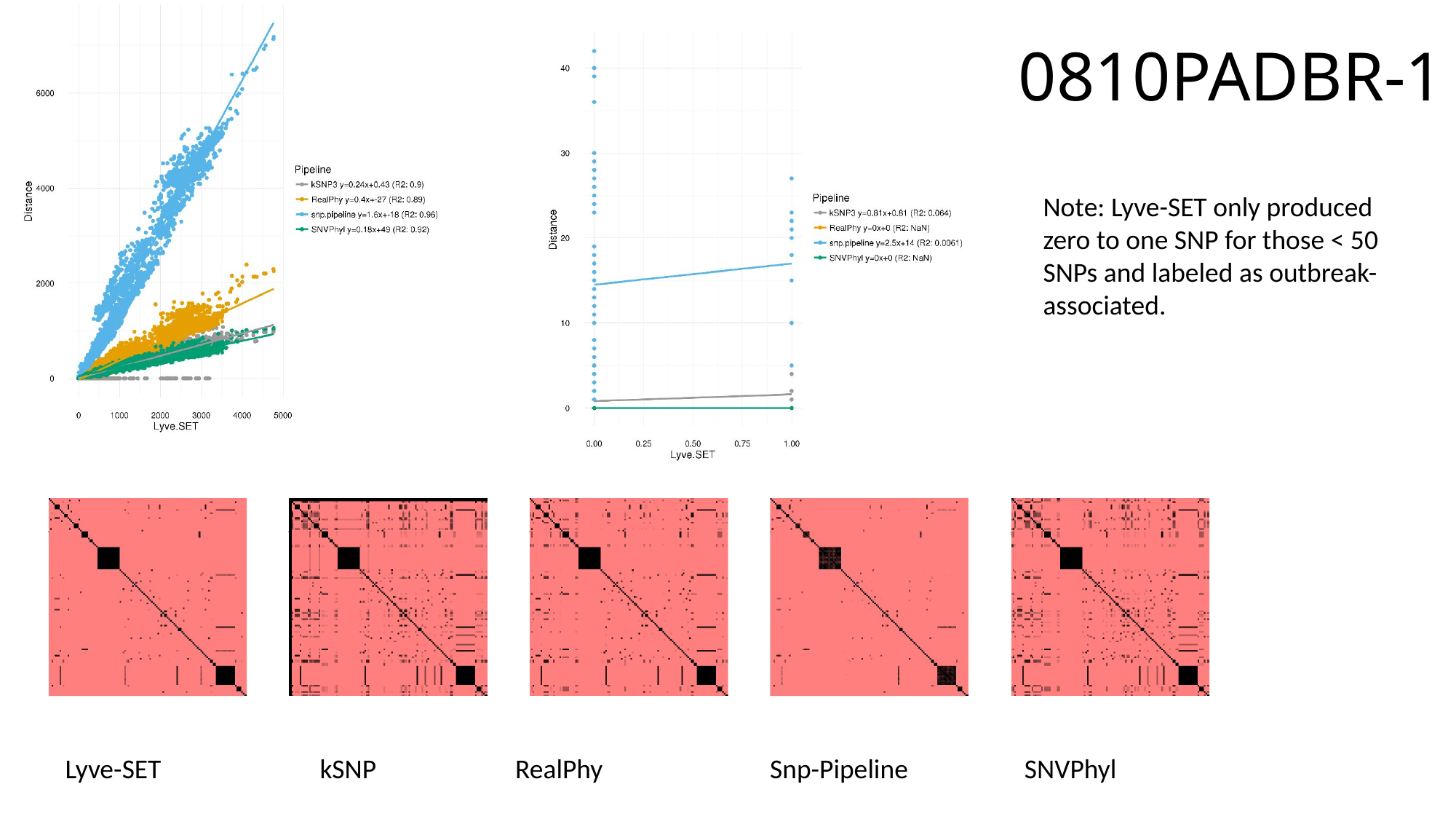

# 0810PADBR-1
Note: Lyve-SET only produced zero to one SNP for those < 50 SNPs and labeled as outbreak-associated.
Lyve-SET
kSNP
RealPhy
Snp-Pipeline
SNVPhyl

## Slide 27
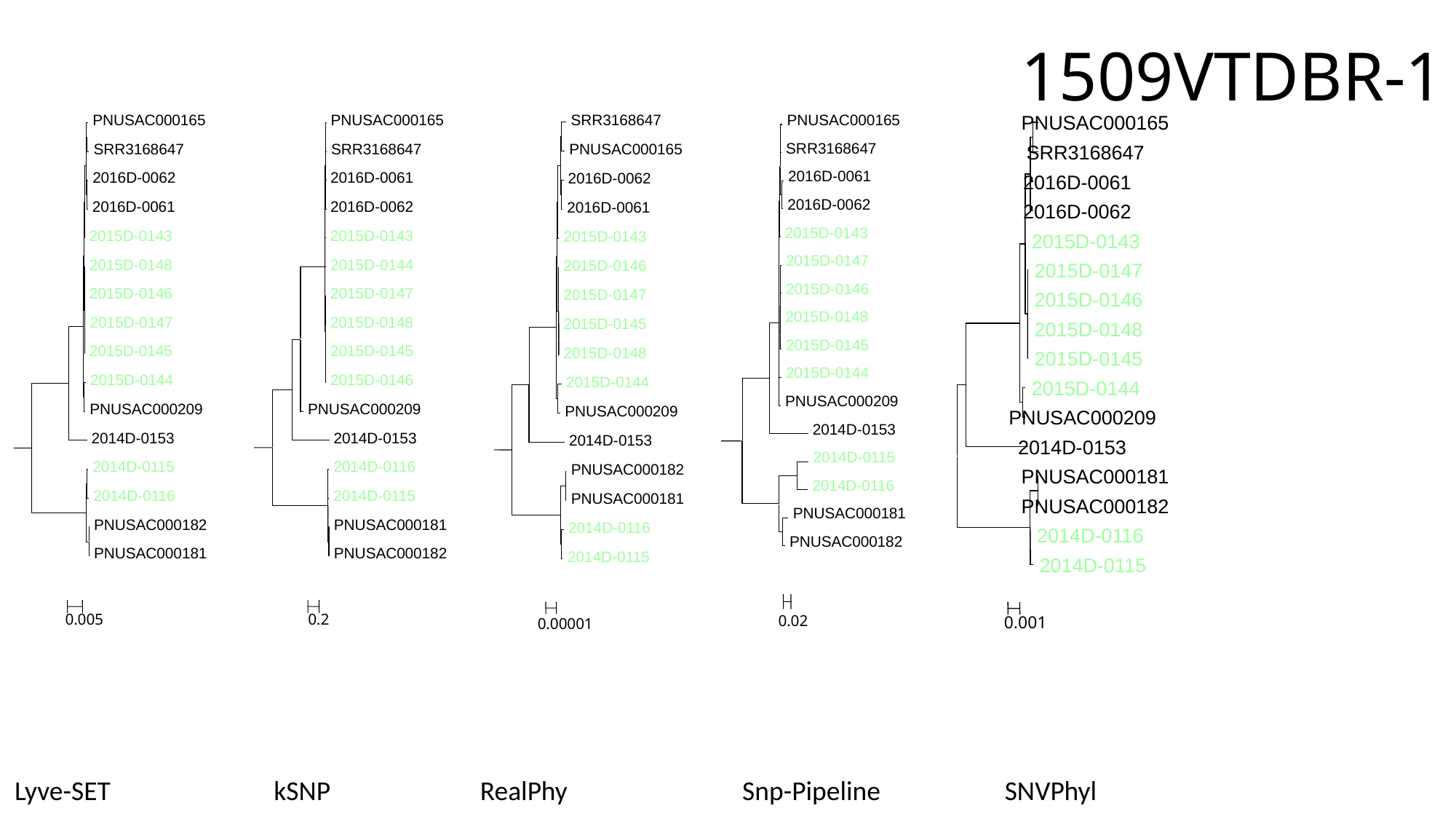

# 1509VTDBR-1
 PNUSAC000165
 SRR3168647
 2016D-0062
 2016D-0061
 2015D-0143
 2015D-0148
 2015D-0146
 2015D-0147
 2015D-0145
 2015D-0144
 PNUSAC000209
 2014D-0153
 2014D-0115
 2014D-0116
 PNUSAC000182
 PNUSAC000181
0.005
 PNUSAC000165
 SRR3168647
 2016D-0061
 2016D-0062
 2015D-0143
 2015D-0144
 2015D-0147
 2015D-0148
 2015D-0145
 2015D-0146
 PNUSAC000209
 2014D-0153
 2014D-0116
 2014D-0115
 PNUSAC000181
 PNUSAC000182
0.2
 SRR3168647
 PNUSAC000165
 2016D-0062
 2016D-0061
 2015D-0143
 2015D-0146
 2015D-0147
 2015D-0145
 2015D-0148
 2015D-0144
 PNUSAC000209
 2014D-0153
 PNUSAC000182
 PNUSAC000181
 2014D-0116
 2014D-0115
0.00001
 PNUSAC000165
 SRR3168647
 2016D-0061
 2016D-0062
 2015D-0143
 2015D-0147
 2015D-0146
 2015D-0148
 2015D-0145
 2015D-0144
 PNUSAC000209
 2014D-0153
 2014D-0115
 2014D-0116
 PNUSAC000181
 PNUSAC000182
0.02
 PNUSAC000165
 SRR3168647
 2016D-0061
 2016D-0062
 2015D-0143
 2015D-0147
 2015D-0146
 2015D-0148
 2015D-0145
 2015D-0144
 PNUSAC000209
 2014D-0153
 PNUSAC000181
 PNUSAC000182
 2014D-0116
 2014D-0115
0.001
Lyve-SET
kSNP
RealPhy
Snp-Pipeline
SNVPhyl

## Slide 28
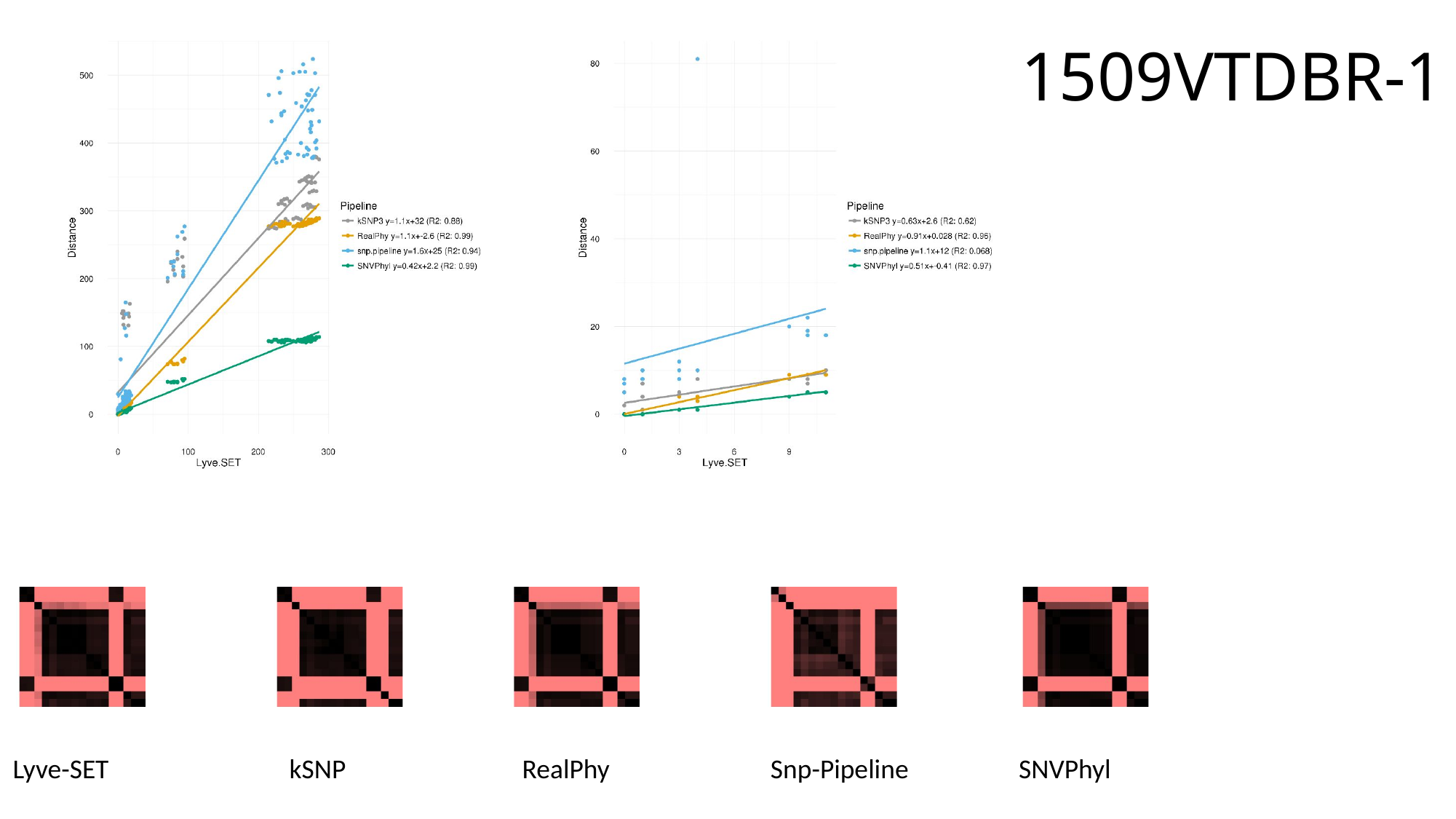

# 1509VTDBR-1
Lyve-SET
kSNP
RealPhy
Snp-Pipeline
SNVPhyl

## Slide 29
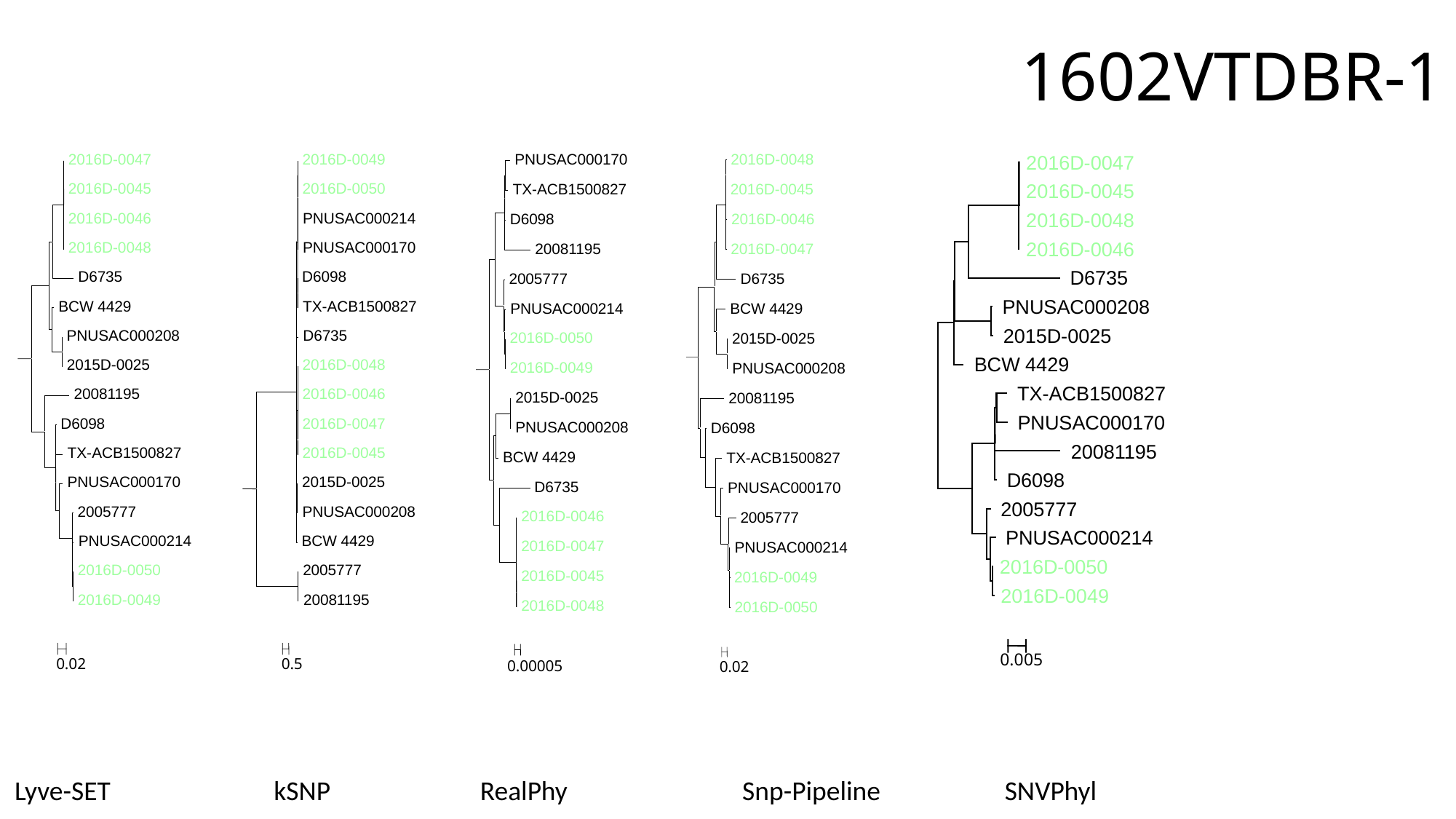

# 1602VTDBR-1
 2016D-0047
 2016D-0045
 2016D-0046
 2016D-0048
 D6735
 BCW 4429
 PNUSAC000208
 2015D-0025
 20081195
 D6098
 TX-ACB1500827
 PNUSAC000170
 2005777
 PNUSAC000214
 2016D-0050
 2016D-0049
0.02
 2016D-0049
 2016D-0050
 PNUSAC000214
 PNUSAC000170
 D6098
 TX-ACB1500827
 D6735
 2016D-0048
 2016D-0046
 2016D-0047
 2016D-0045
 2015D-0025
 PNUSAC000208
 BCW 4429
 2005777
 20081195
0.5
 PNUSAC000170
 TX-ACB1500827
 D6098
 20081195
 2005777
 PNUSAC000214
 2016D-0050
 2016D-0049
 2015D-0025
 PNUSAC000208
 BCW 4429
 D6735
 2016D-0046
 2016D-0047
 2016D-0045
 2016D-0048
0.00005
 2016D-0048
 2016D-0045
 2016D-0046
 2016D-0047
 D6735
 BCW 4429
 2015D-0025
 PNUSAC000208
 20081195
 D6098
 TX-ACB1500827
 PNUSAC000170
 2005777
 PNUSAC000214
 2016D-0049
 2016D-0050
0.02
 2016D-0047
 2016D-0045
 2016D-0048
 2016D-0046
 D6735
 PNUSAC000208
 2015D-0025
 BCW 4429
 TX-ACB1500827
 PNUSAC000170
 20081195
 D6098
 2005777
 PNUSAC000214
 2016D-0050
 2016D-0049
0.005
Lyve-SET
kSNP
RealPhy
Snp-Pipeline
SNVPhyl

## Slide 30
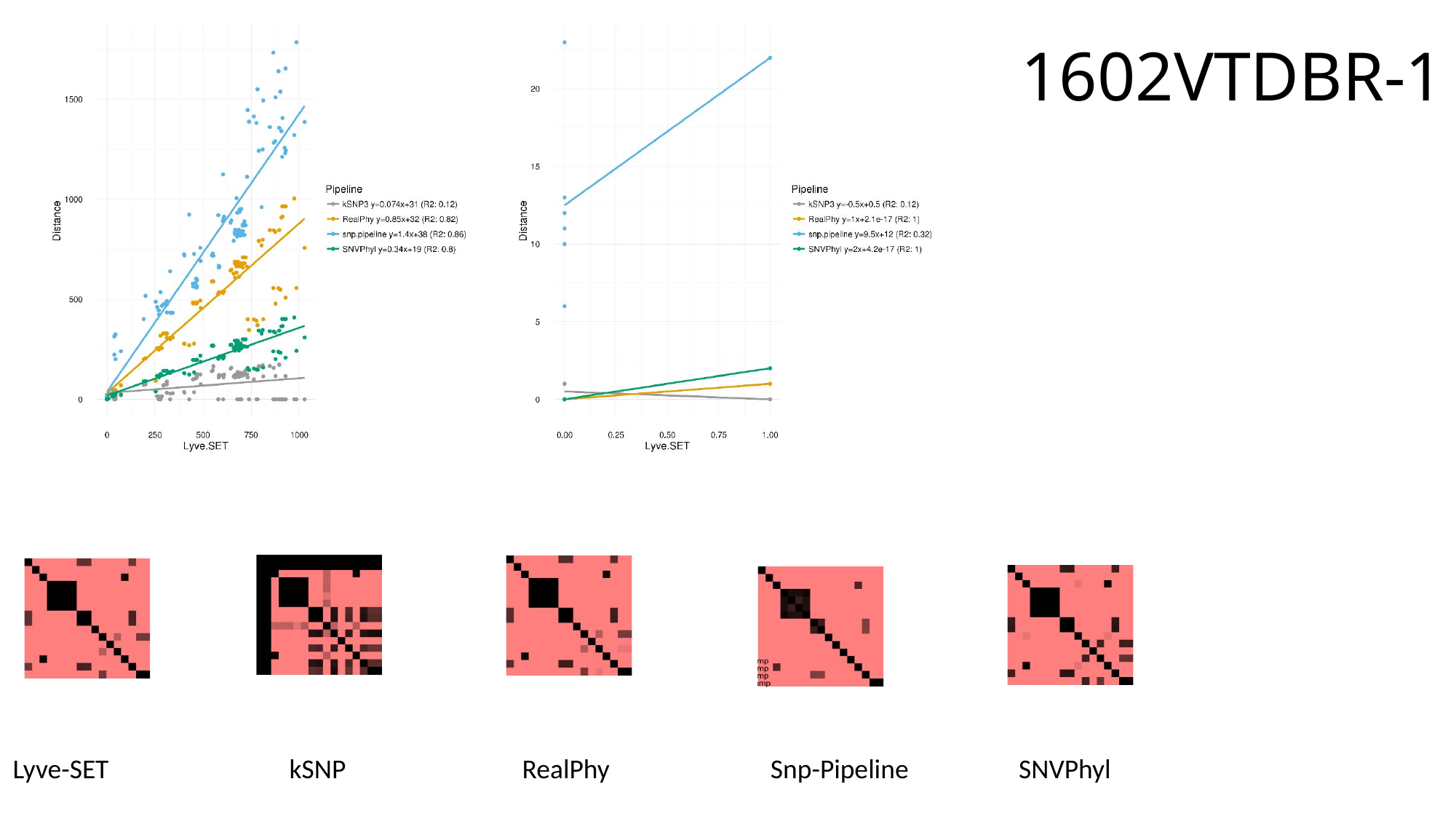

# 1602VTDBR-1
Lyve-SET
kSNP
RealPhy
Snp-Pipeline
SNVPhyl
